# Endemism of subterranean *Diacyclops* in Korea and Japan, with descriptions of seven new species of the *languidoides*-group and redescriptions of *D. brevifurcus* Ishida, 2006 and *D. suoensis* Ito, 1954 (Crustacea, Copepoda, Cyclopoida)

**DOI:** 10.3897/zookeys.267.3935

**Published:** 2013-02-08

**Authors:** Tomislav Karanovic, Mark J. Grygier, Wonchoel Lee

**Affiliations:** 1 Hanyang University, Department of Life Sciences, Seoul 133-791, Korea; 2 University of Tasmania, IMAS, Hobart, Tasmania 7001, Australia; 3 Lake Biwa Museum, Oroshimo 1091, Kusatsu, Shiga 525-0001, Japan

**Keywords:** Taxonomy, stygofauna, interstitial, copepods, zoogeography, Cyclopidae

## Abstract

Copepods have been poorly studied in subterranean habitats in Korea. Previous records have indicated mostly the presence of species already described from Japan, with very few endemic elements. This commonality has usually been explained by repeated dispersal across the land bridges that connected the two countries several times during the Pleistocene glacial cycles. However, the Korean Peninsula is known for pockets of Cambrian and Ordovician carbonate rocks, with more than 1,000 caves already having been explored. The relative isolation of these carbonate pockets makes for an enormous speciation potential, and the development of a high level of short-range endemism of subterranean copepods should be expected. Representatives of the genus *Diacyclops* Kiefer, 1927 are here investigated from a range of subterranean habitats in South Korea, with comparative material sampled from central Honshu in Japan. Morphological analyses of microcharacters, many of which are used in cyclopoid taxonomy for the first time herein, reveal high diversity in both countries. No subterranean species is found in common, although the existence of four sibling species pairs in Korea and Japan may be indicative of relatively recent speciation. We describe seven new stygobiotic species, including three from Korea (*Diacyclops hanguk* **sp. n.**, *Diacyclops leeae* **sp. n.**, and *Diacyclops parasuoensis* **sp. n.**) and four from Japan (*Diacyclops hisuta* **sp. n.**, *Diacyclops ishidai* **sp. n.**, *Diacyclops parahanguk* **sp. n.**, and *Diacyclops pseudosuoensis* **sp. n.**). *Diacyclops hanguk*, *Diacyclops parasuoensis*, *Diacyclops ishidai*, and *Diacyclops parahanguk* are described from newly collected material, while the other three new species are proposed for specimens previously identified as other, widely distributed species. *Diacyclops brevifurcus* Ishida, 2006 is redescribed from the holotype female, and *Diacyclops suoensis* Ito, 1954 is redescribed from material newly collected near the ancient Lake Biwa in Japan. This research provides evidence for the importance of subterranean habitats as reservoirs of biodiversity, and also demonstrates the inadequacy of current morphological methods of identifying closely related species of copepods. The disproportionately high diversity discovered around Lake Biwa provides further evidence in support of the hypothesis about the role of ancient lakes as biodiversity pumps for subterranean habitats. A key to the East Asian species of the *languidoides*-group is provided.

## Introduction

Until recently, freshwater cyclopoids in Korea were studied predominantly in surface water habitats, with 50 species recorded so far (Chang 2009). Only one species, *Acanthocyclops orientalis* Borutzky, 1966, has been reported as a stygobiont, and about six others as stygophiles. In the course of a research project aimed at uncovering Korean invertebrate diversity, and led by the National Institute of Biological Resources (NIBR), subterranean waters were sampled throughout South Korea and identification of copepods was entrusted to the senior author (see Karanovic and Lee 2012; Karanovic et al. 2012b). Two different and independent research projects were conducted in Japan, with partial goals of exploring the groundwater fauna around Lake Biwa and clarifying the role of ancient lakes in the diversity of the subterranean fauna in their vicinity. The latter projects were led by the Lake Biwa Museum (LBM), and identification of copepods was also entrusted to the senior author (see Karanovic and Abe 2010).

Here we present the results for *Diacyclops* Kiefer, 1927, which is the largest genus of the family Cyclopidae Rafinesque, 1815 (see Boxshall and Halsey 2004; Dussart and Defaye 2006; Walter and Boxshall 2012). *Diacyclops* is an ideal group for zoogeographical studies, with different species in surface and subterranean habitats, and some that can exploit both. This genus has a long history of taxonomic problems though (Stoch 2001), and it is recognised to be polyphyletic or at least paraphyletic by many researchers (Monchenko and von Vaupel Klein 1999; Monchenko 2000; Karanovic 2005, 2006). Although the genus name is valid, and its type species is surrounded by a large flock of closely related congeners, ever since its initial erection by Kiefer (1927) as a subgenus of *Cyclops* Müller, 1785 it has had the misfortune of having assigned to it all cyclopoids with even superficially similar fifth legs. Even Kiefer (1927) recognised two distinct groups of species within *Diacyclops*: one containing *Diacyclops bicuspidatus* (Claus, 1857) [type species], *Diacyclops bisetosus* (Rehberg, 1880), *Diacyclops crassicaudis* (Sars, 1863), and some others; and the other group including species with a higher degree of appendage oligomerization, such as *Diacyclops languidus* (Sars, 1863), *Diacyclops languidoides* (Lilljeborg, 1901), and *Diacyclops stygius* (Chappuis, 1924). That Kiefer (1928) was aware of the polyphyletic nature of *Diacyclops* is obvious from his proposed phylogenetic tree (p. 547). Reid and Strayer (1994) noted that the diagnosis of this genus is so broad that it is effectively based solely on the structure of the fifth leg, a character considered probably plesiomorphic by Karanovic (2005, 2006).

Another part of the troubled taxonomic history of *Diacyclops* is the similarity of its fifth leg to that typical for *Acanthocyclops* Kiefer, 1927; many species have been transferred back and forth between these two genera multiple times (see Morton 1985; Pandourski 1997; Monchenko and von Vaupel Klein 1999; Dussart and Defaye 2006; Karanovic et al. 2012a). Ferrari (1991) was probably wrong when he suggested that *Diacyclops* and *Acanthocyclops* belong to two different monophyletic groups of cyclopoids, as he grouped *Acanthocyclops* with *Eucyclops* Claus, 1893 and *Ectocylops* Brady, 1904, which belong to a completely different subfamily. The subfamiliar division of Cyclopidae is well supported both by morphological and molecular evidence (see, for example, Karanovic and Krajicek 2012a).

A proper revision of the *Diacyclops*/*Acanthocyclops* group would require redescriptions of almost 200 nominal species. In addition, evidence for cryptic speciation in some of the more widely distributed taxa (Monchenko 2000; Karanovic and Krajicek 2012b), a common phenomenon in freshwater cyclopoids (Bláha et al. 2010; Karanovic and Krajicek 2012a), must be taken into account. Recent molecular work has suggested polyphyly even of a morphologically well-defined group of *Diacyclops* species inhabiting the subterranean waters of a well-defined region of Australia (Karanovic and Krajicek 2012b). Unfortunately, most of the species of *Diacyclops* have been described from subterranean habitats, from very few specimens, and for some the types are no longer extant or are impossible to trace. All of this makes a comprehensive revision more difficult to accomplish. Some initial attempts have been made, however, to separate obviously unrelated species into newly established genera (Lescher-Moutoué 1976; Reid et al. 1999; Reid and Ishida 2000; Karanovic 2000, 2005; Karanovic et al. 2012a). The general agreement among taxonomists seems to be that the genus must be split into several monophyletic lineages, many of which are recognised as species groups today (Reid and Strayer 1994; Pesce 1996), but also that it must be revised together with the closely related genus *Acanthocyclops*.

In this paper we deal with one such species-group from Korea and Japan, which is characterized by an 11-segmented antennula and a swimming legs segmentation formula of 2/2, 3/2, 3/3, 3/3 (exopod/endopod). This group is usually referred to by the name of its oldest described species, *Diacyclops languidoides*. The *languidoides*-group contains today about one half of all the nominal species and subspecies of *Diacyclops* (see Monchenko and von Vaupel Klein 1999; Dussart and Defaye 2006). These copepods are distributed all over the Holarctic region and are especially diverse in subterranean habitats. Only four species were known previously from Japan: the widely distributed (Palearctic) *Diacyclops languidoides* and the supposedly endemic *Diacyclops japonicus* Ito, 1952, *Diacyclops suoensis* Ito, 1954, and *Diacyclops brevifurcus* Ishida, 2006 (see Ito 1952, 1954; Ueda et al. 1996; Ishida 2006). Ishida (2002) did not cover subterranean taxa in his illustrated fauna of the freshwater cyclopoids of Japan, and thus did not mention either *Diacyclops japonicus* or *Diacyclops suoensis*. He provided a drawing of *Diacylops* sp. B, which he subsequently (Ishida 2006) described as *Diacyclops brevifurcus*, and also some drawings of specimens that he assigned to *Diacyclops nanus* (Sars, 1863), which undoubtedly belong to *Diacyclops languidoides*. Lee et al. (2007) reported two females of *Diacyclops suoensis* from Korea (repeated in Chang 2009), but noted quite a few morphological differences from the Japanese population. Finally, Chang (2009) reported the widely distributed *Diacyclops languidoides* from numerous localities in Korea, as the only other member of the *languidoides*-group here.

The Korean Peninsula is a terrain of low mountains, built mostly of Precambrian rocks, although Paleozoic, Mesozoic, and Cenozoic deposits can be found in many isolated pockets (Lee 1999). Most of the carbonate rocks in Korea are lower Paleozoic (Cambrian to Ordovician) in age and formed in shallow marine environments (Choi and Chough 2005). These sediments are suitable for the formation of subterranean voids, representing an ideal habitat for stygofauna (aquatic subterranean fauna), and more than 1000 caves have been explored so far in Korea, some being as much as 10 km long (Woo et al. 2005). Until recently, copepods in general have been poorly studied in these habitats, and the few available records indicate mostly the presence of species already described from Japan, with very few endemic elements (Chang 2009). Connections between the Korean and Japanese freshwater faunas are indeed very strong (Okazaki et al. 1999; Park et al. 2006), since the two countries were connected by land bridges several times during the Pleistocene glacial cycles (Lihova et al. 2010). However, relative isolation of carbonate pockets makes for an enormous speciation potential, and we expected a high level of short-range endemism in the *languidoides*-group, similar to what is seen in harpacticoid copepods there (Karanovic and Lee 2012; Karanovic et al. 2012b). Surface-water *Diacyclops* are more widely distributed, with no endemic species in Korea (Chang 2009).

## Material and methods

Specimens of the Korean new species were collected by staff of the National Institute of Biological Resources either by the Karaman-Chappuis method, i.e. digging a pit in sandy sediment and decanting through a plankton hand-net (mesh size 38 μm) the water that drains in, or using a phreatic pump. All specimens from Japan collected by senior author were collected by the Karaman-Chappuis method. Animals were fixed in 99% ethanol. Locality data and number of specimens are given in the type material section of the new species below. All Korean types are deposited at the National Institute of Biological Resources, Incheon, while all Japanese types are deposited at the Lake Biwa Museum, Kusatsu.

Specimens were dissected and mounted on microscope slides in Faure’s medium, which was prepared following the procedure discussed by Stock and von Vaupel Klein (1996), and dissected appendages were then covered by a coverslip. For the urosome or the entire animal, two human hairs were mounted between the slide and coverslip, so the parts would not be compressed. By manipulating the coverslip carefully by hand, the whole animal or a particular appendage could be positioned in different aspects, making possible the observation of morphological details. During the examination the water slowly evaporates and the appendages eventually remain in a completely dry Faure’s medium, ready for long-term storage. All line drawings were prepared using a drawing tube attached to a Leica MB2500 phase-interference compound microscope, equipped with N-PLAN (5×, 10×, 20×, 40× and 63× dry) or PL FLUOTAR (100x oil) objectives. Specimens that were not drawn were examined in propylene glycol and, after examination, were again preserved in 99.9% ethanol. Specimens for scanning electron micrography (SEM) were dehydrated in progressive ethanol concentrations, transferred into pure isoamyl-acetate, critical-point dried, mounted on stubs, coated in gold, and observed under a HITACHI S-2380N microscope on the in-lens detector, with an accelerating voltage of 8 kV; black and white photographs were taken on film (Kodak Tri-X 400 pro) and subsequently scanned.

Morphological terminology follows Huys and Boxshall (1991), except for the numbering of the setae of the caudal rami and small differences in the spelling of some appendages (antennula, mandibula, maxillula instead of antennule, mandible, maxillule), as an attempt to standardise the terminology for homologous appendages in different crustacean groups. Biospeleological terminology follows Humphreys (2000). Sensilla and pores on all somites (body segments) were numbered consecutively from the anterior to posterior part of the body and from the dorsal to ventral side, to aid in the recognition of serially homologous structures and future comparisons with other species; they are not intended as a novel terminology. Only the first presented species is described in full, while all subsequent descriptions are shortened by making them comparative.

## Results

### Order Cyclopoida Rafinesque, 1815

Family Cyclopidae Rafinesque, 1815

Subfamily Cyclopinae Rafinesque, 1815

Genus *Diacyclops* Kiefer, 1927

#### Diacyclops ishidai sp. n.

urn:lsid:zoobank.org:act:EFA9BC85-BF1D-44DB-920A-29E39B29A9D9

http://species-id.net/wiki/Diacyclops_ishidai

**Figure 1.**
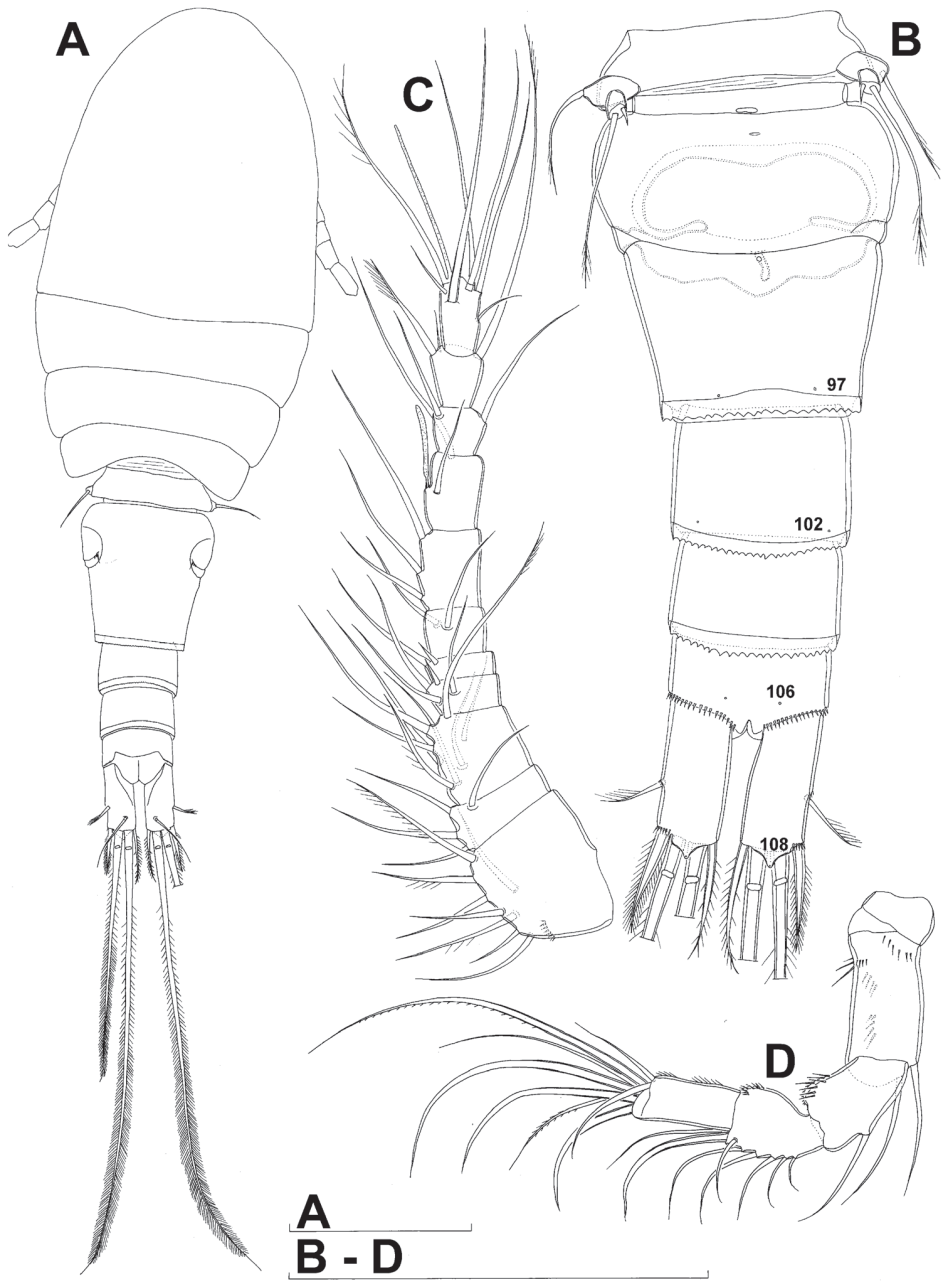
*Diacyclops ishidai* sp. n., holotype female: **A** habitus, dorsal view **B** urosome, ventral view **C** antennula, dorsal view **D** antenna, dorsal view. Arabic numerals numbering sensilla and pores consecutively from anterior to posterior end of body, and from dorsal to ventral side (excluding appendages). Scale bars 100 μm.

Figs 1 [Fig F2] [Fig F3] [Fig F4] [Fig F5] 6 26A, B, C

##### Type locality.

Japan, Shiga prefecture, Otsu city, border of Kamitanakami-Nakano-cho township and Shinme 2-chome district, Kisshoji River about 0.7 km upstream from outflow into Daido River, 34°56.732'N, 135°57.331'E, interstitial water from coarse sand and gravel.

##### Type material.

Holotype female dissected on two slides (LBM 1430005377). Allotype male from type locality also dissected on two slides (LBM 1430005378). Other paratypes from type locality: three females on one SEM stub (LBM1430005379), two females dissected on one slide each (LBM 1430005380, LBM 1430005381), and one male and four females together in ethanol (LBM 1430005382), all collected 27 September 2009, leg. T. Karanovic.

Additional paratypes: eight females in ethanol (LBM 1430005383) from Japan, Shiga prefecture, Otsu city, Nakano 3-chome township, Daido River, 34°57.043'N, 135°57.044'E, interstitial water from medium to coarse sand, 27 September 2009, leg. T. Karanovic.

##### Etymology.

The new species is named in honour of the late Dr. Teruo Ishida, in recognition of his contribution to our knowledge of freshwater copepods in Japan. The name is a noun in the genitive singular.

##### Description.

**Figure 2. F2:**
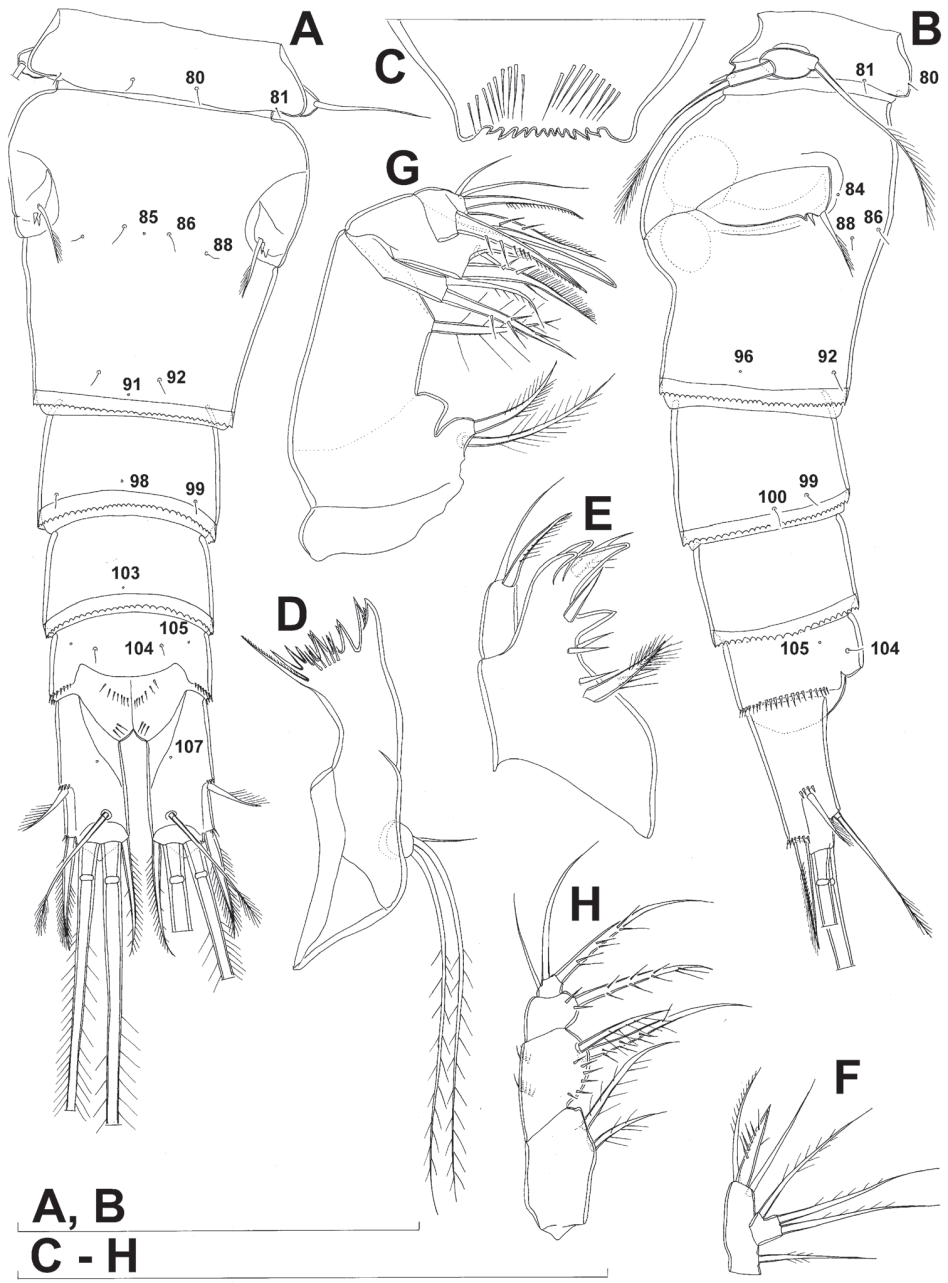
*Diacyclops ishidai* sp. n., holotype female: **A** urosome, dorsal view **B** urosome, lateral view **C** labrum, anterior view **D** mandibula, anterior view **E** maxillula, posterior view (palp armature omitted) **F** maxillular palp, anterior view **G** maxilla, posterior view **H** maxilliped, anterior view. Arabic numerals numbering sensilla and pores consecutively from anterior to posterior end of body, and from dorsal to ventral side (excluding appendages). Scale bars 100 μm.

**Figure 3. F3:**
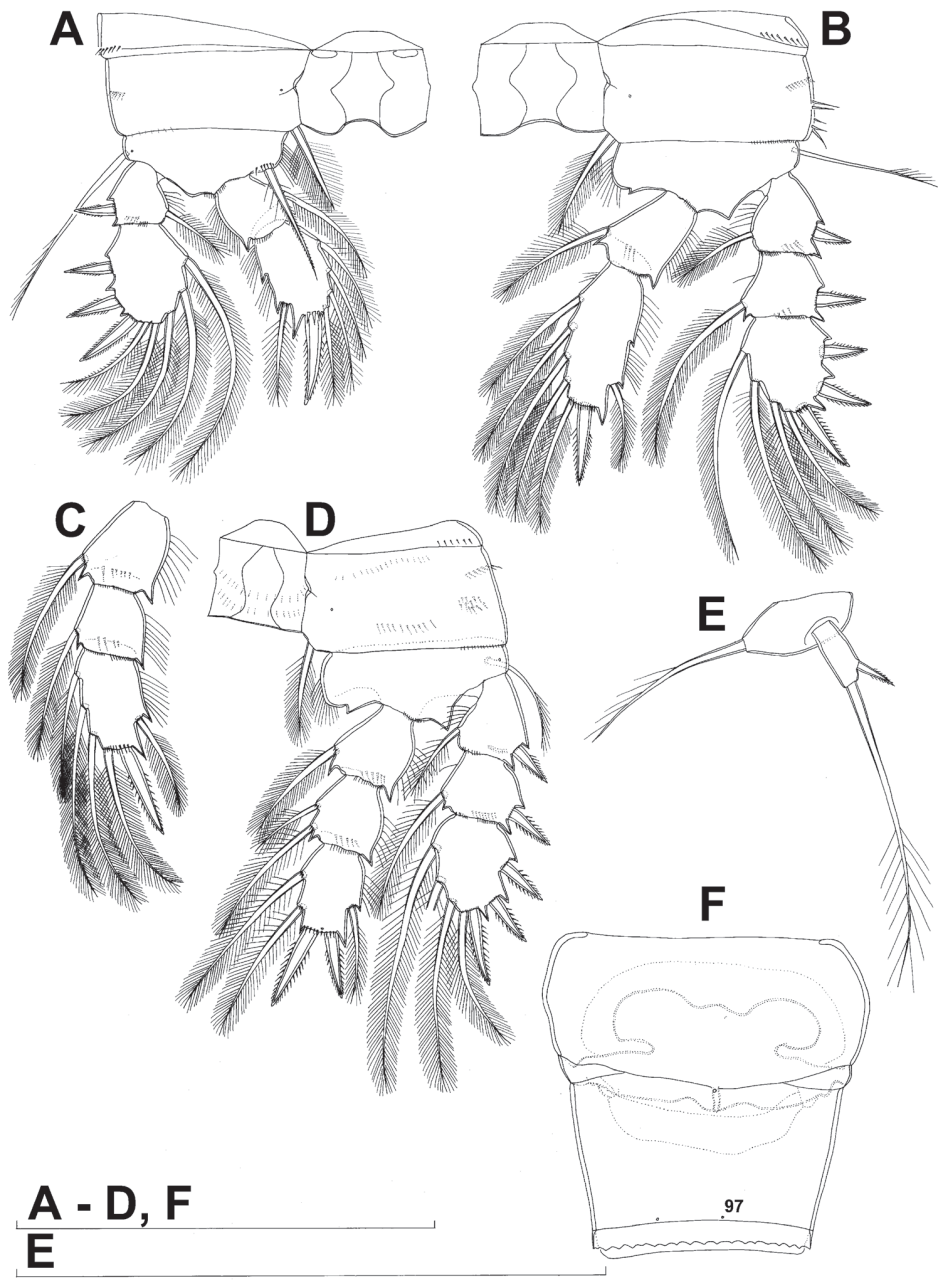
*Diacyclops ishidai* sp. n., **A–E** holotype female **F** paratype female **A** first swimming leg, anterior view **B** second swimming leg, anterior view **C** endopod of third swimming leg, anterior view **D** fourth swimming leg, anterior view **E** fifth leg, anterior view **F** genital double-somite, ventral view. Arabic numerals numbering sensilla and pores consecutively from anterior to posterior end of body, and from dorsal to ventral side (excluding appendages). Scale bars 100 μm.

**Figure 4. F4:**
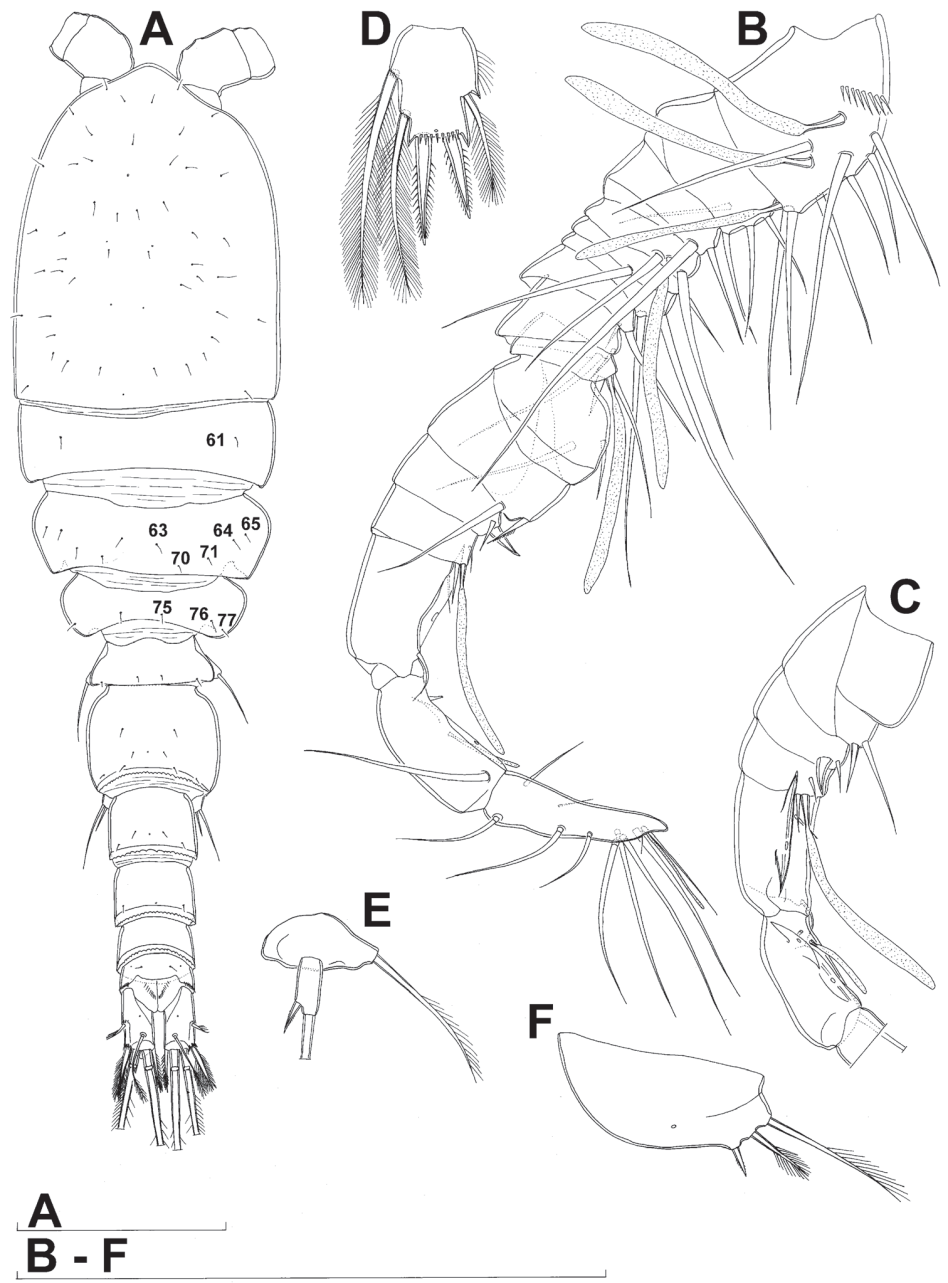
*Diacyclops ishidai* sp. n., allotype male: **A** habitus, dorsal view **B** antennula, flattened and slightly uncoiled, ventral view **C** middle part of antennula, flattened and uncoiled, dorsal view **D** third endopodal segment of fourth swimming leg, anterior view **E** fifth leg, anterior view **F** sixth leg, ventro-lateral view. Arabic numerals numbering sensilla and pores consecutively from anterior to posterior end of body, and from dorsal to ventral side (excluding appendages). Scale bars 100 μm.

**Figure 5. F5:**
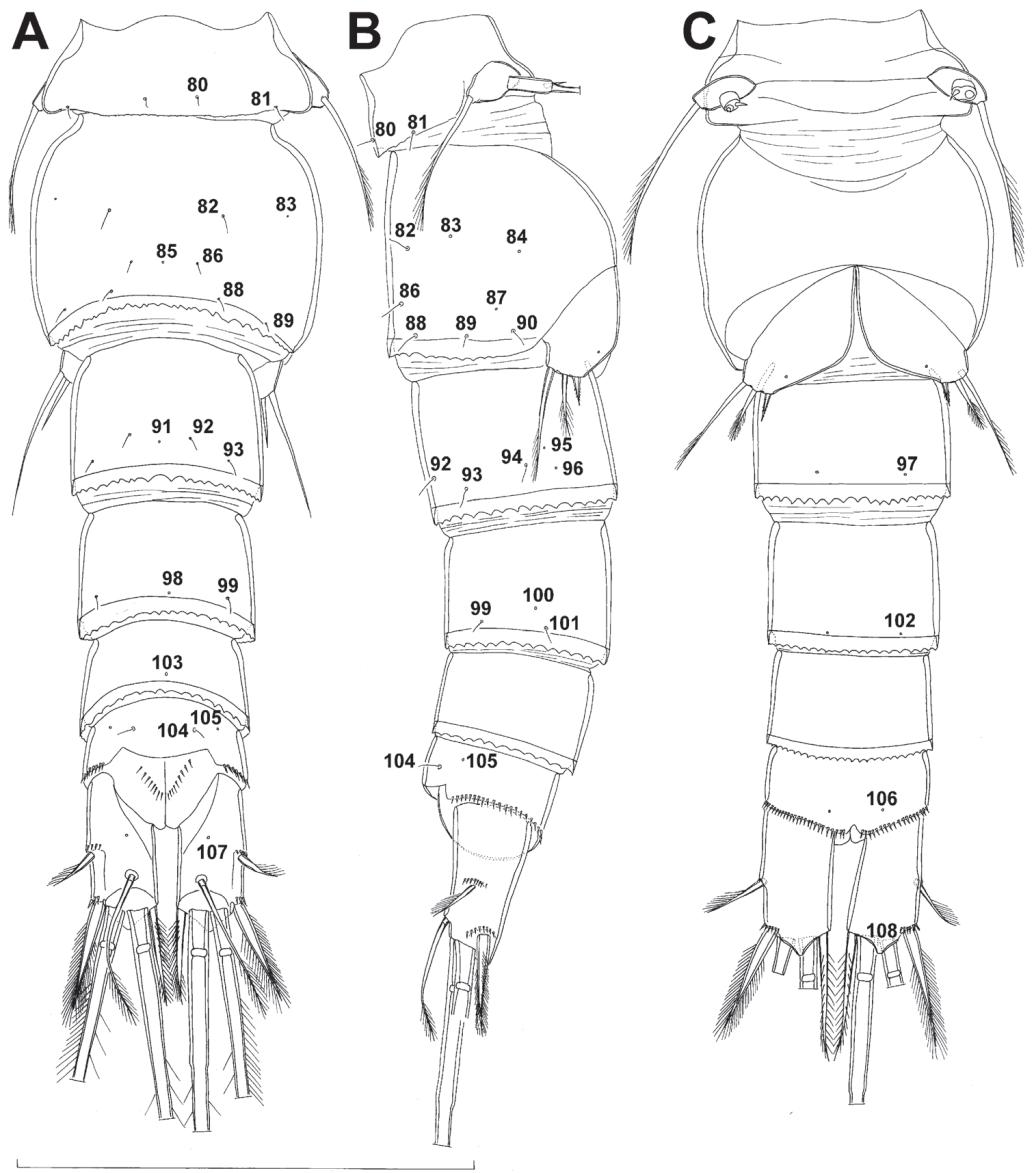
*Diacyclops ishidai* sp. n., allotype male: **A** urosome, dorsal view **B** urosome, lateral view **C** urosome, ventral view. Arabic numerals numbering sensilla and pores consecutively from anterior to posterior end of body, and from dorsal to ventral side (excluding appendages). Scale bar 100 μm.

**Figure 6.**
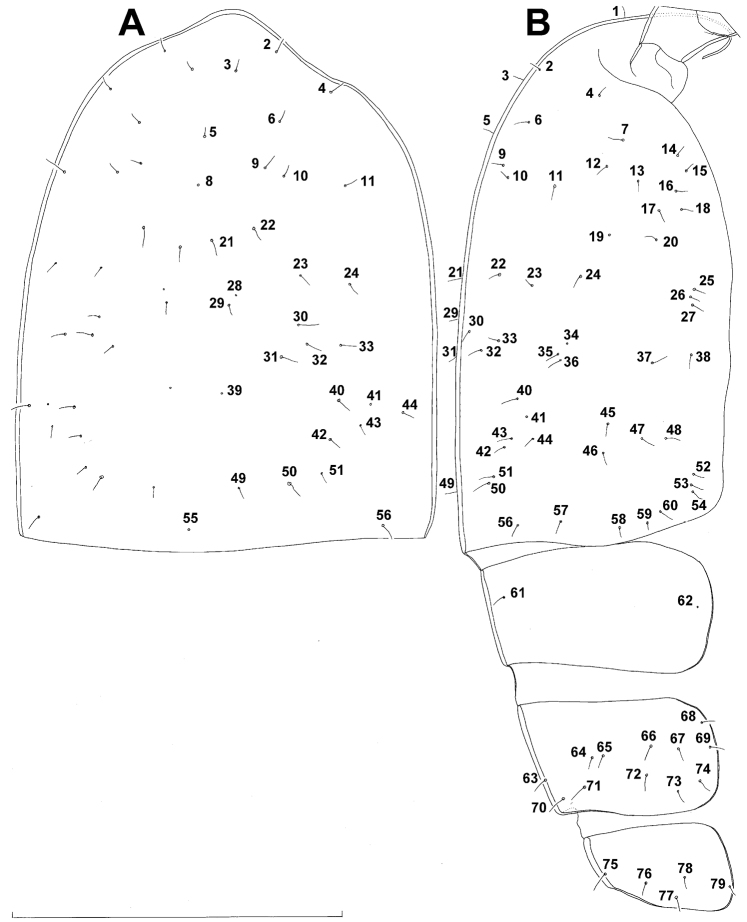
*Diacyclops ishidai* sp. n., allotype male: **A** cephalothorax, dorsal view **B** cephalothoracic shield and pleurons of free prosomites, lateral view. Arabic numerals numbering sensilla and pores consecutively from anterior to posterior end of body, and from dorsal to ventral side (excluding appendages). Scale bar 100 μm.

Female (based on holotype and five paratypes from type locality). Total body length, measured from tip of rostrum to posterior margin of caudal rami (excluding caudal setae), from 450 to 482 µm (453 µm in holotype). Preserved specimens colourless; no live specimens observed. Integument relatively weakly sclerotised, smooth, without cuticular pits or cuticular windows. Surface ornamentation of somites consisting of 92 pairs and seven unpaired (mid-dorsal) pores and sensilla (numbered with Arabic numerals consecutively from anterior to posterior end of body, and from dorsal to ventral side in Figs 1B, 2A, B, 3F; but illustrated in more detail for male specimens, see Figs 4, 5, 6); no spinules except on anal somite, caudal rami, and appendages. Habitus (Fig. 1A) relatively robust, not dorso-ventrally compressed, with prosome/urosome length ratio 1.3 and greatest width in dorsal view at posterior end of cephalothorax. Body length/width ratio about 3 (dorsal view); cephalothorax 2.1 times as wide as genital double-somite. Free pedigerous somites without lateral or dorsal expansions, all connected by well developed arthrodial membranes and all with narrow and smooth hyaline fringes. Pleural areas of cephalothorax and free pedigerous somites relatively well developed, covering insertions of cephalic appendages and praecoxae and partly covering coxae of swimming legs in lateral view.

Rostrum well developed, membranous, not demarcated at base, broadly rounded and furnished with single frontal pair of sensilla (no. 1).

Cephalothorax (Fig. 1A) large, 1.1 times as long as its greatest width (dorsal view), narrower at anterior part and perfectly oval; representing 38% of total body length. Surface of cephalic shield ornamented with three unpaired mid-dorsal sensilla and pores (nos. 5, 8, 55) and 56 pairs of long sensilla and small cuticular pores (nos. 2-5, 6, 7, 9-54, 56-60); pores and sensilla 39-60 belonging to first pedigerous somite, latter being incorporated into cephalothorax.

Second pedigerous (first free) somite (Figs 1A) relatively short, tapering posteriorly, ornamented with just one pair of dorsal sensilla (no. 61) and one pair of lateral pores (no. 62); serially homologous pairs impossible to establish.

Third pedigerous somite (Fig. 1A) slightly longer than second and significantly narrower in dorsal view, ornamented with 12 pairs of large sensilla (nos. 63-74); recognition of serially homologous pairs not easy, but probably dorsolateral pair of sensilla no. 64 serially homologous to pair no. 61 on second pedigerous somite.

Fourth pedigerous somite (Figs 1A) significantly shorter and narrower than third, with slightly flared latero-posterior corners and only five pairs of large sensilla (nos. 75–77); recognition of serially homologous pairs slightly easier than for two previous prosomites (probably 75=63, 76=71, 77=72, 78=73, 79=74).

Fifth pedigerous (first urosomal) somite (Figs 1B, 2A, B) short, significantly narrower than fourth pedigerous somite and also narrower than genital double-somite in dorsal view, ornamented with two pairs of large dorsal sensilla (nos. 80, 81); recognition of serially homologous pairs easy, i.e. 80=75 and 81=77; hyaline fringe very narrow, smooth or barely visibly serrated.

Genital double-somite (Figs 1B, 2A, B, 3F) large, swollen antero-ventrally with deep lateral recesses at level of sixth legs, widest at first quarter of its length and gradually tapering posteriorly, only slightly longer than its greatest width (dorsal view), ornamented with one unpaired central dorsal pore (no. 85), two pairs of central dorsal sensilla (nos. 86, 88), one pair of lateral central pores (no. 84), one unpaired posterior dorsal pore (no. 91), one pair of posterior sensilla (no. 92), and two pairs of ventro-lateral posterior pores (nos. 96, 97); central dorsal sensilla probably serially homologous to those on fifth pedigerous somite (i.e. 86=80, 88=81), but recognition of serial homologies of posterior sensilla and pores much harder (perhaps 91=85, 92=86); hyaline fringe deeply and irregularly serrated. Copulatory pore very small, oval, situated ventrally at about midlength of double-somite; copulatory duct narrow, siphon-shaped, weakly sclerotised. Seminal receptacle characteristically shaped, with relatively large anterior expansion, constriction at midlength, and shorter but broader posterior expansion, altogether representing 49% of double-somite’s length. Ovipores situated dorso-laterally at 1/3 length of double-somite, covered by reduced sixth legs.

Third (ancestral fourth) urosomite (Figs 1B, 2A, B) relatively short, about 1.8 times as wide as long and less than 0.4 times as long as genital double-somite in dorsal view, also with deeply and irregularly serrated hyaline fringe, ornamented with unpaired dorsal posterior pore (no. 98), two pairs of dorso-lateral posterior sensilla (nos. 99, 100), and one pair of ventro-lateral posterior pores (nos. 102); serially homologous pores and sensilla not easy to recognize on genital double-somite, except 98=91.

Fourth (preanal) urosomite (Figs 1B, 2A, B, 26A, B) narrower and shorter than third, also with deeply and irregularly serrated hyaline fringe; ornamented only with unpaired dorsal pore (no. 103), serially homologous to pore no. 98 on third urosomite.

Anal somite (Figs 1B, 2A, B, 26A, B) slightly narrower and significantly shorter than preanal somite, with short medial cleft; ornamented with one pair of large dorsal sensilla (no. 104), one pair of small dorsal pores (no. 105), one pair of small ventral pores (no. 106), continous posterior row of small spinules, and two diagonal parallel rows of somewhat larger spinules on both sides of anal sinus. Anal operculum wide, short, slightly convex, not reaching posterior margin of anal somite, representing 59% of anal somite’s width.

Caudal rami (Figs 1A, B, 2A, B, 26A) cylindrical, parallel, inserted close to each other, with deep dorso-median anterior depression (as continuation of anal sinus), approximately twice as long as wide (ventral view) and twice as long as anal somite; armed with six setae (one dorsal, one lateral, and four terminal); ornamented with one dorsal pore (no. 107), one pore on tip of large protuberance on distal margin ventrally between two terminal setae (no. 108), and rows of small spinules at base of lateral setae. Dorsal seta slender, about as long as ramus, inserted at 5/6 of ramus length, biarticulate at base (inserted on small pseudo-joint), and pinnate distally. Lateral seta inserted at 2/3 of ramus length, 0.4 times as long as dorsal seta, unipinnate laterally and uniarticulate at base. Outermost terminal seta stout, spiniform, 0.7 times as long as ramus, densely bipinnate. Innermost terminal (accessory) seta slightly longer and more slender than outermost terminal seta, sparsely pinnate along outer margin and densely pinnate along inner margin. Principal terminal setae with breaking planes, bipinnate; inner principal terminal seta about 1.8 times as long as outer one and 6.6 times as long as caudal rami.

Antennula (Fig. 1C) 11-segmented, slightly curved along caudal margin, directed postero-laterally, not reaching posterior margin of cephalothoracic shield, ornamented only with proximo-ventral arc of spinules on first segment (no pits or other integumental structures), with armature formula as follows (ae = aesthetasc): 8.4.8.4.2.2.3.2+ae.2.3.7+ae. Only one of terminal seta on ultimate segment biarticulate basally and most of longer setae sparsely pinnate distally; both aesthetascs very slender, that on eighth segment reaching distal margin of ninth segment. One seta on fourth and one on fifth segment spiniform and short, all other setae slender; one apical seta on 11th segment fused basally to aesthetasc. Length ratio of antennular segments, from proximal end and along caudal margin, 1 : 0.3 : 0.9 : 0.4 : 0.3 : 0.6 : 1 : 0.9 : 0.6 : 0.7 : 1.

Antenna (Fig. 1D) five-segmented, strongly curved along caudal margin, comprising very short coxa, much longer basis, and three-segmented endopod. Coxa without armature or ornamentation, about half as long as wide. Basis cylindrical, 1.8 times as long as wide, ornamented with two short diagonal rows of spinules on ventral surface, two transverse rows of small spinules on dorsal surface, and three large spinules along caudal margin, also armed with two subequal smooth setae on distal inner corner (exopodal seta absent). First endopodal segment narrowed basally but generally cylindrical, 1.6 times as long as wide and 0.8 times as long as basis, with smooth inner seta at 2/3 length and patch of large spinules along caudal margin. Second endopodal segment also with narrowed basal part, 1.4 times as long as wide, about 0.8 times as long as first segment, bearing eight smooth setae along inner margin (these progressively longer from proximal to distal), ornamented with one row of spinules along caudal margin. Third endopodal segment cylindrical, 2.3 times as long as wide and slightly longer than second endopodal segment, ornamented with two rows of slender spinules along caudal margin, armed with eight apical setae (four of them strong and geniculate; two pinnate).

Labrum (Fig. 2C) a relatively large trapezoidal plate, with muscular base and strongly sclerotised distal margin (cutting edge), ornamented with two short diagonal rows of nine long and slender spinules each on anterior surface. Cutting edge almost straight, with 11 sharp teeth between produced and rounded lateral corners.

Mandibula (Fig. 2D) composed of coxa and small palp. Cutting edge of coxal gnathobase with five slender spinules on anterior surface, eight apical teeth, and dorsalmost unipinnate seta; ventralmost tooth strongest and quadricuspidate, second and fourth teeth from ventral side bicuspidate, all other teeth unicuspidate; three dorsalmost simple teeth partly fused basally and progressively longer from ventral to dorsal. Palp twice as wide as long, unornamented, armed with three apical setae, two of them long and bipinnate and one short and smooth; pinnate setae subequal in length, directed posteriorly, not reaching posterior margin of cephalic shield.

Maxillula (Fig. 2E, F) composed of praecoxa and two-segmented palp, unornamented. Praecoxal arthrite bearing four very strong distal spines (three of them smooth, blunt, and fused at base; one distinct at base, sharp and with single proximal spinule) and six medial elements (proximalmost one longest and plumose, two most distal ones large and strong, three in between small and slender). Palp composed of coxobasis and one-segmented endopod. Coxobasis with slender proximal seta (probably representing exopod) and three medial setae (two slender, one strong). Endopod with three slender pinnate setae.

Maxilla (Figs 2G) 5-segmented but praecoxa partly fused to coxa on anterior surface, unornamented. Proximal endite of praecoxa robust, armed with two subequal, sparsely bipinnate setae; distal endite small, unarmed. Proximal endite of coxa with one bipinnate seta; distal endite highly mobile, elongated and armed apically with two pinnate setae, proximal one of which considerably longer and stronger. Basis expanded into robust claw; claw furnished with longitudinal row of four spinules at midlength and armed with two setae: strong seta about as long as claw and pinnate, small seta smooth and slender. Endopod two-segmented, but segmentation not easily discernable; proximal segment armed with two robust, unipinnate setae; distal segment with one robust, unipinnate apical seta and two slender and much shorter subapical setae. Longest seta on distal endopodal segment 0.8 times as long as longer seta on proximal endopodal segment. All strong setae on basis and endopod, as well as basal claw, unguiculate.

Maxilliped (Fig. 2H) four-segmented, composed of syncoxa, basis, and two-segmented endopod. Ornamentation consisting of three rows of long, slender spinules on basis (two transverse rows on posterior surface close to outer margin and one longitudinal row on anterior surface close to inner margin), as well as two spinules on anterior surface of first endopodal segment. Armature formula: 2.2.1.3. All inner setae pinnate, very strong, and unguiculate.

All swimming legs (Figs 3A, B, C, D, 26C) relatively small, composed of minute, triangular praecoxa, large, rectangular coxa, short basis, and slender exopod and endopod. Exopods and endopods approximately equally long in all legs, their segmentation formula (exopod/endopod): 2/2.3/2.3/3.3/3. Ultimate exopodal segment spine formula 3.3.3.3 and setal formula 5.4.4.4. All setae on endopods and exopods slender and plumose, except apical seta on exopod of first leg pinnate along outer margin and plumose along inner (Fig. 3A); no modified setae observed. All spines strong and bipinnate. Intercoxal sclerite of all swimming legs with slightly concave distal margin and lacking surface ornamentation, except on posterior surface of fourth leg.

First swimming leg (Fig. 3A) shorter than other swimming legs; praecoxa unarmed, ornamented with distal row of small spinules on anterior surface; coxa 2.3 times as wide as long, ornamented with short, transverse row of spinules on posterior surface close to outer margin, distal row of minute spinules on anterior surface, and small pore on anterior surface close to inner margin, armed with long, plumose seta on inner-distal corner; basis almost pentagonal, 0.8 times as long as coxa, armed with long, slender outer seta and strong, bipinnate inner-distal element (latter reaching to 2/3 length of second endopodal segment), ornamented with row of slender spinules along inner margin, two posterior rows of shorter and stronger spinules on anterior surface (one at base of inner seta, other at base of endopod), and one cuticular pore on anterior surface close to outer margin; exopod with single outer spine and single inner seta on first segment, with three outer spines and five setae (three inner, two apical) on second segment, ornamented with distal rows of spinules on both anterior and posterior surfaces of first segment, row of slender inner spinules on first segment, and extremely minute spinules at base of almost all setae and spines on anterior surface; endopod armed only with inner seta on first segment, second segment with four inner setae, one apical spine, and one outer seta, ornamented with slender spinules along inner margins of both segments, with shorter and stronger spinules along distal margin of first segment on anterior surface, and minute spinules at base of most setae on anterior surface (those at base of apical spine larger); apical spine on second endopodal segment slightly outwardly unguiculate, about as long as segment and only slightly shorter than inner setae; second endopodal segment about 1.4 times as long as wide and also 1.4 times as long as first endopodal segment, with small inner notch showing ancestral segmentation.

Second swimming leg (Fig. 3B) longer than first leg; coxa 2.2 times as wide as long, armed with plumose inner seta (slightly longer than in first leg), ornamented with five large spinules along outer margin, in addition to short row of small spinules on posterior surface and distal row of minute spinules and small pore on anterior surface; basis with somewhat shorter outer seta than in first leg, and without inner seta, with very small spiniform process instead; exopod three-segmented and longer than in first leg, with outer spine and inner seta on first and second segments, and with three outer spines and four setae on third segment (three inner, one apical), ornamented with distal rows of spinules on first and second segments, minute spinules at base of all setae and spines (Fig. 26C), as well as pores at distal outer corners of all three segments, these pores being situated on anterior surface but opening laterally and thus invisible by light microscopy (Fig. 26C); endopod with second segment longer than in first leg and armed with five inner setae; apical spine on second endopodal segment proportionally shorter than on first leg, 0.6 times as long as segment or distal inner seta; second endopodal segment about 2.1 times as long as wide and 1.6 times as long as first endopodal segment.

Third swimming leg (Fig. 3C) similar to second leg in shape, size, and armature, except endopod three-segmented; apical spine on third endopodal segment slightly shorter than segment and 0.6 times as long as apical seta; and third endopodal segment about 1.5 times as long as wide and 1.4 times as long as second endopodal segment.

Fourth swimming leg (Fig. 3D) generally similar to third swimming leg, but slightly shorter and more slender, with longer and more plumose setae, two parallel transverse rows of long spinules on posterior surface of intercoxal sclerite and coxa, two inner setae on second endopodal segment, and two inner setae, two apical spines, and one outer seta on third endopodal segment; third endopodal segment with pore on anterior surface, about 1.3 times as long as wide, and 1.2 times as long as second endopodal segment; inner apical spine on third endopodal segment 1.3 times as long as outer apical spine, as long as segment, and less than 0.6 times as long as distal inner seta; apical spines diverging at about 30° angle.

Fifth leg (Figs 1B, 2B, 3E) inserted ventro-laterally, relatively small, two-segmented. First segment (possibly protopod) broad and short, almost rhomboidal, half as long as greatest width, unornamented, armed with single slender outer seta (probably ancestral outer basal seta), this being inserted on extremely long setophore and unipinnate distally. Second segment (probably exopod) much narrower, cylindrical, 1.2 times as long as first segment and 2.3 times as long as wide, unornamented, armed with apical long seta and subapical inner spine; apical exopodal seta bipinnate distally, 1.7 times as long as basal seta, 4.6 times as long as exopod, and more than seven times as long as subapical spine, but only reaching midlength of genital double-somite; subapical exopodal spine small but strong, bipinnate, 0.64 times as long as exopod and 1.5 times as long as exopod’s greatest width.

Sixth leg (Fig. 2A, B) small, short and broad semicircular cuticular plate armed with two short, smooth spines and one longer and distally unipinnate outer seta; inner spine fused to plate, outer articulated basally; outermost seta directed postero-dorsally.

Male (based on allotype and one paratype from type locality). Total body length 448–473 µm (473 µm in allotype). Urosome with free genital somite. Habitus (Fig. 4A) more slender than in female, with prosome/urosome length ratio about 1.4 and greatest width in dorsal view at posterior end of cephalothorax. Body length/width ratio 3.7; cephalothorax about twice as wide as genital somite. Cephalothorax 1.3 times as long as wide (dorsal view); representing 35% of total body length. Ornamentation of cephalothorax (Figs 4A, 6A, B), free prosomites (Figs 4A, 6B), and first and last two urosomites (Fig. 5A, B, C) with same number and distribution of sensilla and pores as in female.

Genital somite (Fig. 5A, B, C) 1.5 times as wide as long in dorsal view, with serrated hyaline fringe dorsally, ornamented with one unpaired dorsal pore (no. 85), six pairs of dorsal and lateral sensilla (nos. 82, 86, 88-90), and three pairs of lateral pores (nos. 83, 84, 87); pores and sensilla nos. 82, 83, 87, 89 not present in female; no spermatophores visible inside. Third urosomite (Fig. 5A, B, C) homologous to posterior part of female genital double-somite, ornamented with dorsal unpaired pore (no. 91) and pores and sensilla nos. 92, 96, 97, but additionally with two pairs of lateral sensilla (nos. 93, 94) and one pair of lateral pores (no. 95). Fourth urosomite (Fig. 5A, B, C) similar to that of female, but ornamented with one additional pair of lateral sensilla (no. 101). Fifth urosomite (Fig. 5A, B, C) as in female. Anal somite (Fig. 5A, B, C) slightly shorter than in female, and with only one diagonal row of spinules on each side of anal sinus, but with other ornamentation and proportions of anal operculum as in female.

Caudal rami (Fig. 5A, B, C) slightly shorter than in female and with proportionally shorter innermost terminal seta, but with very similar ornamentation and armature to those of female; inner principal terminal seta with small constriction at start of pinnation.

Antennula (Figs 6F, 8B, C, D) strongly prehensile and digeniculate, 16-segmented (with ancestral 16th and 17th segments completely fused), ornamented with spinules only on first segment (as in female), with anvil-shaped cuticular ridges on anterior margin of 14th and 15th segments (distal geniculation). Armature formula as follows: 8+3ae.4.2.2+ae.2.2.2.2.2+ae.2.2.2.2 + ae.2.1+ae.11+ae. All aesthetascs linguiform and most relatively long and broad, apical one on 16th segment fused basally to one seta; most setae slender and smooth; short smooth setae on seventh (one) eighth (one), ninth (one), tenth (one), 12th (two), 13th (two), and 14th segments; short pinnate seta on 11th segment; six setae on 16th segment biarticulate distally or with breaking plane.

Antenna, labrum, mandibula, maxillula, maxilla, first swimming leg, second swimming leg, and third swimming leg as in female.

Fourth swimming leg (Fig. 4D) also with similar armature and ornamentation to that of female; third endopodal segment 1.8 times as long as wide; inner apical spine on third endopodal segment 1.3 times as long as outer apical spine and nearly as long as segment.

Fifth leg (Fig. 4E) similar to that of female, but with slightly shorter subapical exopodal spine.

Sixth leg (Fig. 4F) a large cuticular plate ornamented with single pore on anterior surface, armed with inner spine and two setae on outer distal corner; outermost seta unipinnate and 2.2 times as long as inner bipinnate seta, as well as 5.2 times as long as innermost spine.

##### Remarks.

**Figure 7.**
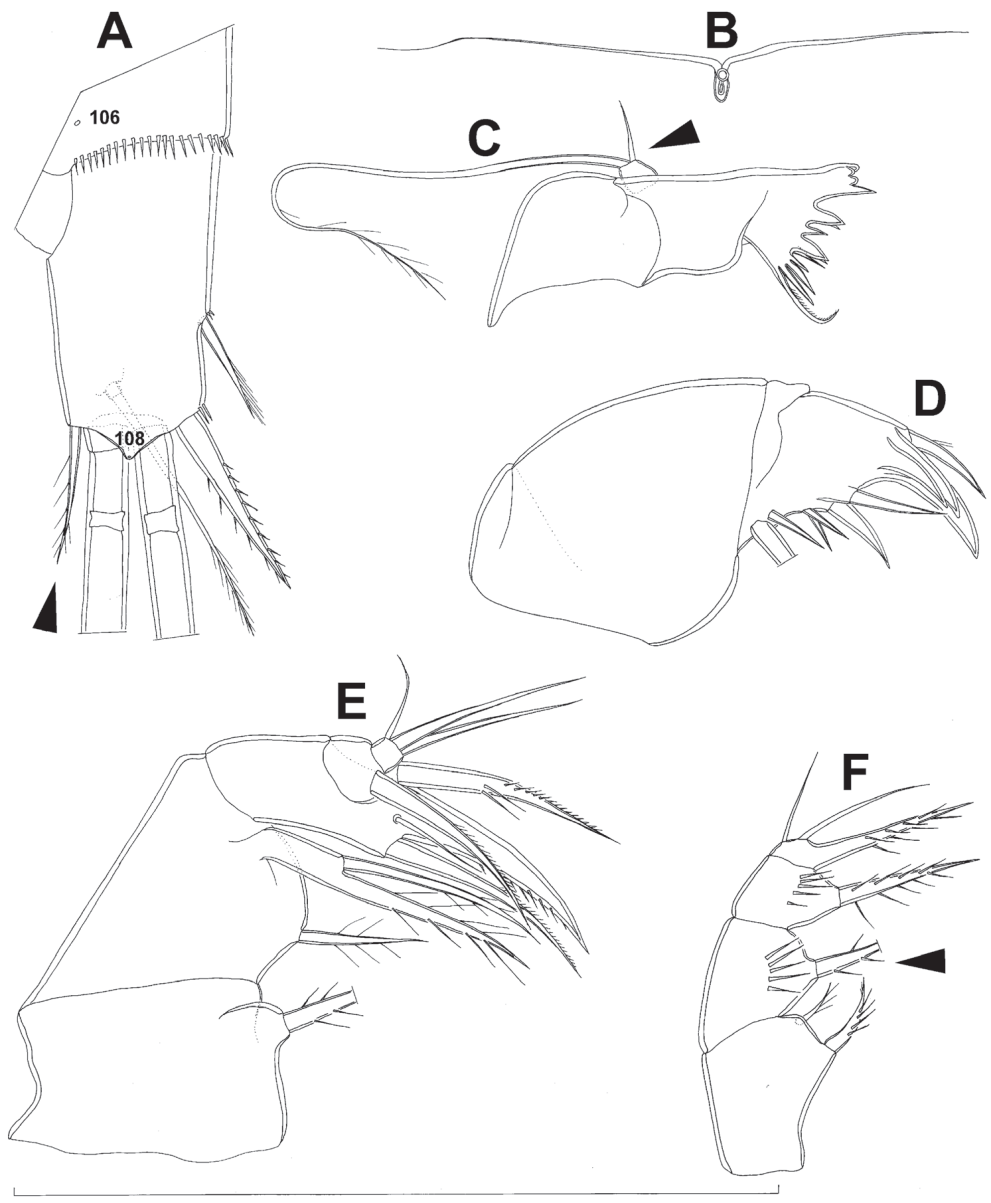
*Diacyclops brevifurcus* Ishida, 2006, holotype female: **A** left caudal ramus, ventral view **B** copulatory pore, ventral view **C** mandibula, posterior view **D** maxillula, posterior view (palp broken off) **E** maxilla, anterior view **F** maxilliped, anterior view. Arabic numerals indicating sensilla and pores presumably homologous to those in *Diacyclops ishidai* sp. n. Arrows pointing most prominent specific features. Scale bar 100 μm.

**Figure 8.**
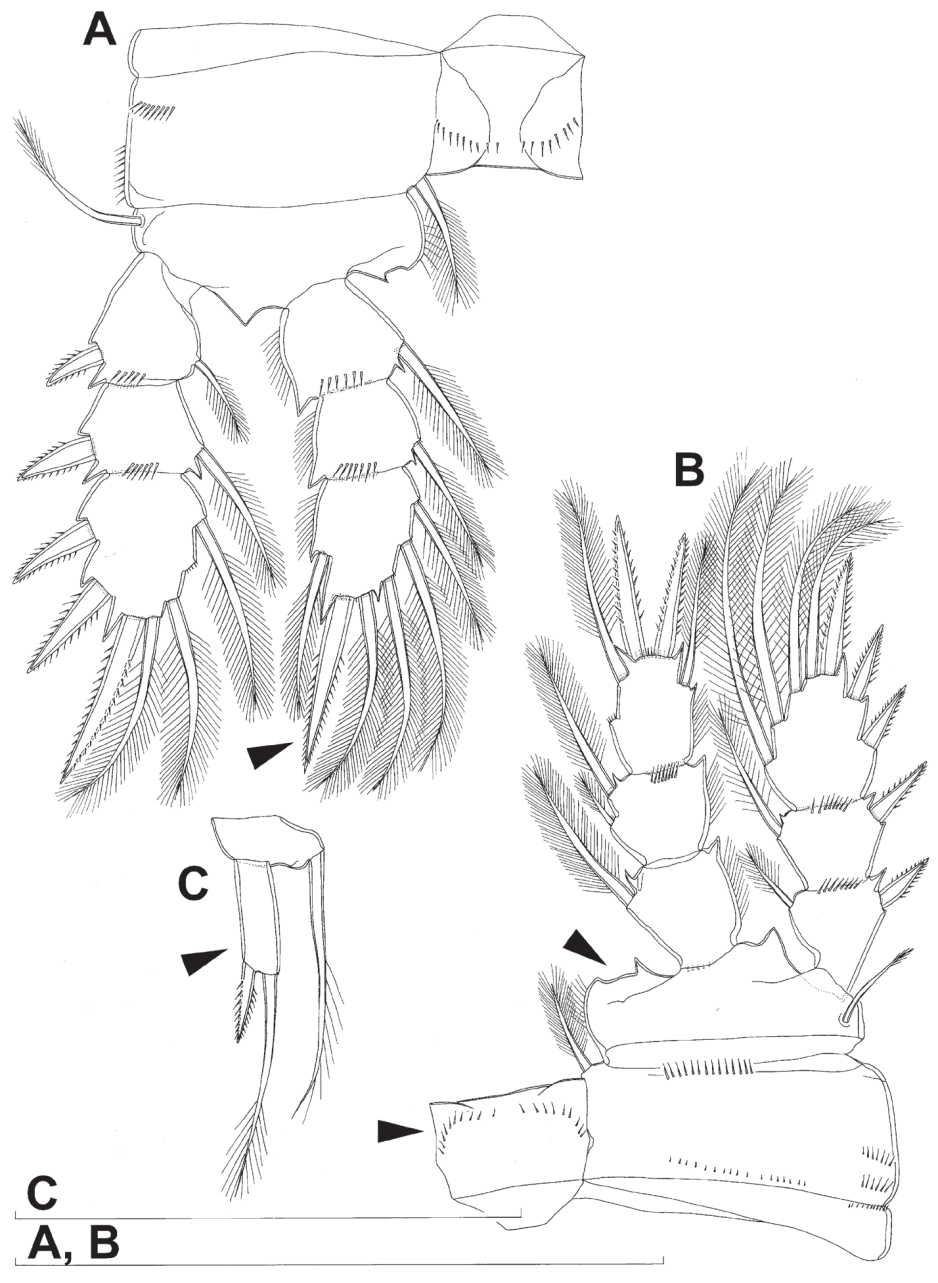
*Diacyclops brevifurcus* Ishida, 2006, holotype female: **A** third swimming leg, posterior view **B** fourth swimming leg, posterior view **C** fifth leg, anterior view. Arrows pointing most prominent specific features. Scale bars 100 μm.

This species is probably most closely related to *Diacyclops brevifurcus* Ishida, 2006, which was described from Kyoto on the other side of Mount Hiei, in a parallel drainage basin to that of Lake Biwa (Ishida 2006), and from Lake Biwa itself. The description of *Diacyclops brevifurcus* was quite lacking in detail, so we redecribed it after examining the holotype specimen dissected on one slide and four specimens from Lake Biwa (see below). *Diacyclops brevifurcus* shares with *Diacyclops ishidai* sp. n. the shape of the caudal rami, which are unusually short for the *languidoides*-group, and have an extremely large ventral protuberance on the distal margin with a pore on the tip. Both species also share exactly the same armature formula of all swimming legs as well as an identical pattern of cuticular pores and sensilla on all somites. Differences between the two include: relative length of the innermost terminal caudal seta, number of setae on the mandibular palp, relative length of the apical spine on the third leg endopod, number of rows of spinules on the intercoxal sclerite of the fourth leg, shape of the inner-distal margin of the fourth leg basis, and proportions of the distal segment of the fifth leg (all these arrowed in Figs 7, 8), as well as the armature of the antenna (with an exopodal seta present and only seven setae each on the second and third exopodal segments in *Diacyclops brevifurcus*). Ishida (2006) illustrated two setae on the basis of the maxilliped in *Diacyclops brevifurcus*, but only one is present in the holotype (arrowed in Fig. 7F). Ishida (2002) partly illustrated a specimen from Lake Biwa under the name *Diacyclops* sp. B, which he later (2006) attributed to *Diacyclops brevifurcus* (see the synonymy section below). We examined the four extant specimens from this series and agree that they are conspecific with *Diacyclops brevifurcus* (see below). Ishida erroneously illustrated the antenna of *Diacyclops* sp. B with only six setae on the second endopodal segment, and the antennula with only ten segments, but he later corrected himself in the description of *Diacyclops brevifurcus*.

Only four other species of the *languidoides*-group have similarly short caudal rami: *Diacyclops ichnusae* Pesce & Galassi, 1985 from Sardinia; *Diacyclops ichnusoides* Petkovski & Karanovic, 1997 from the ancient Lake Ohrid; *Diacyclops improcerus* (Mazepova, 1950) and *Diacyclops versutus* (Mazepova, 1962) from the ancient Lake Baikal (see Mazepova 1950, 1962; Pesce and Galassi 1985; Petkovski and Karanovic 1997). Unfortunately, all four were described based on a limited set of morphological characters, and many features cannot be compared with those of *Diacyclops ishidai* and *Diacyclops brevifurcus*. Five out of these six species come from or near ancient lakes, a distribution pattern commonly associated with Tertiary relics. *Diacyclops ishidai* has a shorter dorsal caudal seta and a more rounded seminal receptacle than *Diacyclops ichnusae*, longer caudal rami and third endopodal segment of the fourth leg than *Diacyclops improcerus*, a more slender habitus, longer female antennula, and shorter inner spine on the male sixth leg than *Diacyclops versutus*, and a shorter dorsal caudal seta, no exopodal seta on the antenna, and a longer third endopodal segment of the fourth leg than *Diacyclops ichnusoides*.

#### Diacyclops brevifurcus

Ishida, 2006

http://species-id.net/wiki/Diacyclops_brevifurcus

Figs 7 8

Diacyclops sp. B – Ishida 2006): p. 57, fig. 28a–j.
Diacyclops brevifurcus sp. n. – Ishida 2006): p. 41, figs 1, 2.

##### Type locality.

Japan, Kyoto prefecture, Kyoto city, Kita ward, Mizoro-ga-ike pond, approximately 35°03.43'N, 135°46.11'E, floating bog bed.

##### Material examined.

Holotype female dissected on one slide (LBM 1430000887), 1 February 2005, leg. A. Ohtaka.

Other material: three females in ethanol (LBM 1430000888) and one female dissected on one slide (LBM 1430005384) from Japan, Shiga prefecture, Takashima city, Makinocho-Kaizu district, Lake Biwa on western side of Kaizu Ohsaki point, between near-shore boulders, 07 March 1994, leg. T. Ishida. Note: Consultation of original field data maintained at the Lake Biwa Museum showed that the the municipality (town) mentioned by Ishida (2006), viz., Nishiazai, was erroneous and that the collection site is actually in what was then the Kaizu district of Makino town.

##### Partial redescription.

Female (holotype). Cephalothoracic shield and pleurons of free prosomites missing from slide (only one piece of cephalothorax present), so pattern of cuticular pores and sensilla not observed. Preserved specimen yellowish. Integument relatively weakly sclerotised, smooth, without cuticular pits or cuticular windows.Urosome squashed and badly deformed, but similar to original drawing by Ishida (2006), with posterior ventral pair of pores observed on each of genital double-somite, third urosomite, and anal somite. Copulatory pore (Fig. 7B) small, oval, situated ventrally at about midlength of genital double-somite; copulatory duct narrow, siphon-shaped, well sclerotised, supported by pronounced transverse internal sclerotised ridge.

Anal somite (Fig. 7A) with short and broad anal operculum, ornamented with one pair of large dorsal sensilla (no. 104), one pair of small dorsal pores (no. 105), one pair of small ventral pores (no. 106), continous posterior row of small spinules, and two diagonal parallel rows of somewhat larger spinules on both sides of anal sinus. Anal operculum short, wide, slightly convex, reaching to midlength of anal somite, representing 50% of anal somite’s width.

Caudal rami (Fig. 7A) somewhat squashed on slide, but cylindrical in shape, parallel, inserted close to each other, with deep dorso-median anterior depression (as continuation of anal sinus), approximately twice as long as wide (ventral view) and twice as long as anal somite; ornamentation and armature as in *Diacyclops ishidai* sp. n., but innermost terminal seta much shorter and more slender (arrowed in Fig. 7A), only about 0.7 times as long as outermost terminal seta.

Antennula 11-segmented, generally as illustrated by Ishida (2006), but with additional seta on each of third, fourth, and sixth segments, giving same armature formula as in *Diacyclops ishidai*.

Antenna five-segmented, generally as illustrated by Ishida (2006), i.e. basis with strong exopodal seta, second endopodal segment with seven setae along inner margin, and third endopodal segment with seven terminal setae.

Labrum completely deformed on slide, impossible to illustrate.

Mandibula (Fig. 7C) composed of coxa and small palp. Cutting edge of coxal gnathobase without spinules on anterior surface, furnished with eight apical teeth and dorsalmost unipinnate seta; ventralmost tooth strongest and quadricuspidate, second and fourth teeth from ventral side bicuspidate, all other teeth unicuspidate; three dorsalmost simple teeth partly fused basally and progressively longer from ventral to dorsal. Palp about as wide as long, unornamented, armed with only two apical setae, one long and distally bipinnate, the other short and smooth; pinnate seta about 1.5 times as long as whole mandibula.

Maxillula (Fig. D) composed of praecoxa and palp (but palp broken off and missing from slide), unornamented. Praecoxal arthrite bearing four very strong distal spines (three of them smooth, blunt, and fused at base; one distinct at base, sharp and with two proximal spinules) and six medial elements (proximalmost one broken off, two most distal ones large and strong, three in between smaller and slender).

Maxilla (Fig. 7E) 5-segmented but praecoxa partly fused to coxa on anterior surface, unornamented. Proximal endite of praecoxa robust, armed with one sparsely bipinnate seta; distal endite very small, unarmed. Proximal endite of coxa with one bipinnate seta; distal endite highly mobile, elongated and armed apically with two pinnate setae, proximal one of which slightly longer but considerably stronger and basally fused to endite. Basis expanded into robust claw, and claw furnished with longitudinal row of spinules along concave (dorsal) margin, armed with two setae; strong seta slightly stronger than claw, unipinnate along convex (ventral) margin; small seta smooth and slender. Endopod two-segmented, with segmentation easily discernable; proximal segment armed with two robust, unipinnate setae; distal segment with one robust and two slender subapical setae, all smooth. Longest seta on distal endopodal segment 0.8 times as long as longer seta on proximal endopodal segment. All strong setae on basis and endopod, as well as basal claw, gently unguiculate.

Maxilliped (Fig. 7F) four-segmented, composed of syncoxa, basis, and two-segmented endopod. Ornamentation consisting of longitudinal rows of five large spinules on anterior surface of basis and first endopodal segment. Armature formula: 2.1.1.3. All inner setae pinnate, relatively strong but short and not unguiculate; inner seta on second endopodal segment basally fused to segment.

All swimming legs relatively small, composed of minute, triangular praecoxa, large, rectangular coxa, short basis, and slender exopod and endopod. Exopods and endopods approximately equally long on all legs, their segmentation formula (exopod/endopod): 2/2.3/2.3/3.3/3. Ultimate exopodal segment spine formula 3.3.3.3 and setal formula 5.4.4.4. All setae on endopods and exopods slender and plumose, except apical seta of exopod of first leg pinnate along outer margin and plumose along inner; no modified setae observed. All spines strong and bipinnate. Intercoxal sclerite of all swimming legs with slightly concave distal margin and without any surface ornamentation on first and second leg, but with arc of spinules on posterior margin of third (Fig. 8A) and fourth (Fig. 4B) legs.

First and second swimming legs as in *Diacyclops ishidai*.

Third swimming leg (Fig. 8A) also generally similar to that of *Diacyclops ishidai* in shape, size and armature; apical spine on third endopodal segment proportionately much longer, about 1.5 times as long as segment and 0.9 times as long as apical seta; third endopodal segment about 1.2 times as long as wide and 1.4 times as long as second endopodal segment.

Fourth swimming leg (Fig. 8B) generally similar to that of *Diacyclops ishidai* in shape, but intercoxal sclerite with only one row of spinules (arrowed in Fig. 8B), and inner distal corner of basis with two spiniform processes separated by shallow notch (arrowed in Fig. 8B); proximal inner seta on second endopodal segment proportionally shorter than that of *Diacyclops ishidai* and apical spines longer and more robust; third endopodal segment about 1.4 times as long as wide, and 1.4 times as long as second endopodal segment; inner apical spine on third endopodal segment less than1.2 times as long as outer apical spine, 1.2 times as long as segment, and more than 0.7 times as long as distal inner seta.

Fifth leg (Fig. 8C) with much shorter protopod than in *Diacyclops ishidai*, but with longer and more robust exopod (arrowed in Fig. 8C) and subapical exopodal spine, and with shorter apical exopodal seta. Exopod cylindrical, 2.2 times as long as protopod and 2.9 times as long as wide; apical exopodal seta bipinnate distally, 0.85 times as long as basal seta, twice as long as exopod, and three times as long as subapical spine, but reaching beyond midlength of genital double-somite; subapical exopodal spine small but strong, bipinnate, 0.7 times as long as exopod and nearly twice as long as exopod’s greatest width.

Sixth leg completely deformed on slide, but generally similar to that of *Diacyclops ishidai*, consisting of a small, short, and broad semicircular cuticular plate armed with two short, smooth spines and one longer and distally unipinnate outermost seta; inner spine fused to plate, outer one articulated basally; outermost seta directed postero-dorsally.

Male. Unknown.

##### Variability.

The specimens from Lake Biwa show several features in the mouthparts that differ from those observed in the holotype. They have two long setae on the mandibular palp, two setae on the proximal endite of the maxilla, two setae on the basis of the maxilliped, and three setae on the syncoxa of the maxilliped. All other features agree well with those of the holotype, including the length of the innermost terminal caudal seta, armature of the antenna, shape of the notch on the fourth leg basis, and shape of the fifth leg exopod. The specimens from Lake Biwa have ten teeth and six or seven spinules on the labrum, and the same pattern of cuticular sensilla and pores on the somites as that in *Diacyclops ishidai*, but most of these characters cannot be compared with the holotype.

##### Remarks.

This is probably a surface-water species, but its morphological similarity to the newly described *Diacyclops ishidai* (see above) warranted its re-examination and inclusion in this paper. Ishida (2006) described it from a single female from Mizoro-ga-ike pond in Kyoto, and we redescribe it here based on an examination of the same holotype, mounted on one slide. In the same work, Ishida reported another three damaged females from Lake Biwa, but as other material examined, not as paratypes. We had a chance to examine this material as well and found four females in the vial, only one of which was in poor condition (with the posterior part of the usorome missing). We dissected one of them on one slide, while the others were examined undissected. All four specimens are indeed conspecific with *Diacyclops brevifurcus* Ishida, 2006, but with several differences in the mouth appendages (see variability above). It is hard to assess whether these differences are a result of geographical variation, or are just an abnormality in the holotype, because only one specimen from Mizoro-ga-ike pond was collected. *Diacyclops brevifurcus* is probably most closely related to *Diacyclops ishidai*, which we collected in the interstitial of two localities on the banks of the Daido River, which joins the Seta River, Lake Biwa’s outflow, several kilometers below the outflow point and is then part of the same water system as the lake. The morphological similarities and differences between these two species are outlined in the Remarks section for *Diacyclops ishidai* (see above).

#### Diacyclops parasuoensis sp. n.

urn:lsid:zoobank.org:act:AD07B77C-E244-4358-8B2E-ABF063271B75

http://species-id.net/wiki/Diacyclops_parasuoensis

**Figure 9.**
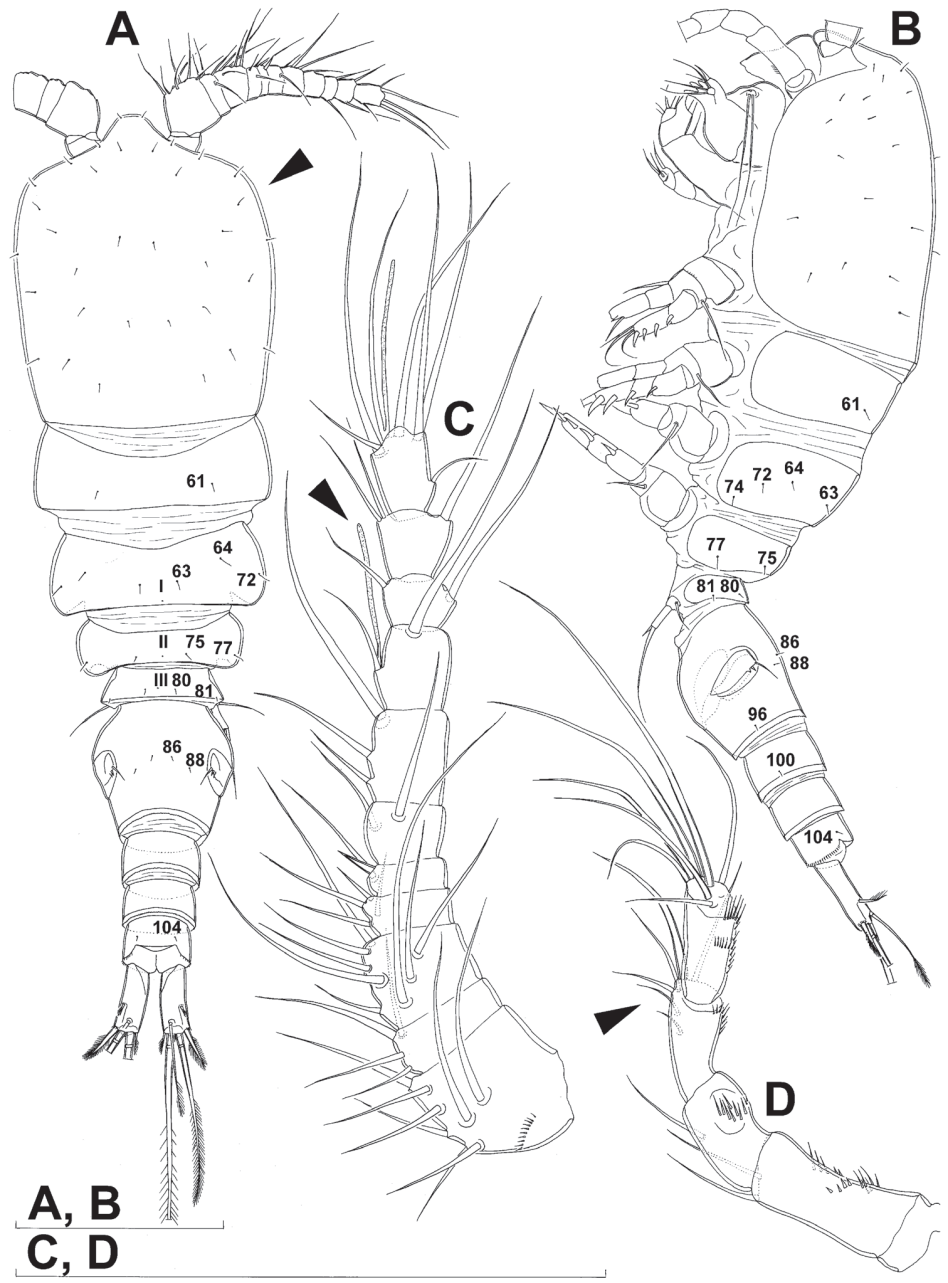
*Diacyclops parasuoensis* sp. n., holotype female: **A** habitus, dorsal view **B** habitus, lateral view **C** antennula, ventral view **D** antenna, dorsal view. Arabic numerals indicating sensilla and pores presumably homologous to those in *Diacyclops ishidai* sp. n. Roman numerals indicating pores not present in *Diacyclops ishidai* sp. n. Arrows pointing most prominent specific features. Scale bars 100 μm.

Figs 9 [Fig F10] [Fig F11] [Fig F12] 13 26D, E 27C

##### Type locality.

South Korea, Jeollanamdo, Gurye city, Yangcheon, Seomjin River, 35°12'04.7"N, 127°35'29.3"E, interstitial water from coarse sand and gravel.

**Type material.** Holotype female dissected on two slides (NIBRIV0000232650). Allotype male from type locality dissected on two slides (NIBRIV0000232651). Other paratypes from type locality: four males, 15 females and 19 copepodids together in ethanol (NIBRIV0000232652), two males and eigth females on one SEM stub (NIBRIV0000232653); two males and three females on one SEM stub (NIBRIV0000232648), two females dissected on one slide each (NIBRIV0000232670 & NIBRIV0000232670), and two males dissected on one slide each (NIBRIV0000232654 & NIBRIV0000232655); all collected 19 June 2010, leg. J.-L. Cho.

Additional paratypes: seven males, three females and five copepodids together in alcohol (NIBRIV0000232656), from South Korea, Jeollanamdo, Gurye city, Yangcheon, Seomjin River (different locality), 35°11'25.4"N, 127°23'00.7"E, interstitial water from sandy banks, 19 June 2010, leg. J.-L. Cho.

Additional paratypes: two males, two females, and one copepodid together in alcohol (NIBRIV0000232657), from South Korea, Gangwondo, Wonju city, Jijeong, Seom River, 37°23'30.16"N, 127°51'08.39"E, interstitial water from sandy banks, 24 June 2010, leg. J.-L. Cho.

Additional paratypes: one female and seven copepodids together in alcohol (NIBRIV0000232658), from South Korea, Gangwondo, Wonju city, Buron River, 37°14'01.23”N, 127°44'58.78”E, interstitial water from sandy banks, 24 June 2010, leg. J.-L. Cho.

Additional paratypes: two males, one female, and seven copepodids together in alcohol (NIBRIV0000232659), from South Korea, Gyungsangbukdo, Sangju city, Young River, 36°31'42.8"N, 128°14'02.7"E, interstitial water from sandy banks, 1 July 2010, leg. J.-L. Cho.

Additional paratypes: two males and seven females together in alcohol (NIBRIV0000232660), from South Korea, Gyungsangbukdo, Uljin city, Geunnam, Wangpi stream, 36°57'41.4"N, 129°22'46.4"E, interstitial water from sandy banks, 18 May 2010, leg. J.-L. Cho.

##### Etymology.

The species name is an adjective composed of the existing specific name *suoensis* and the Greek prefix *para* (= near, beside), and refers to the relatively close apparent relationship between these two congeners.

##### Description.

**Figure 10. F10:**
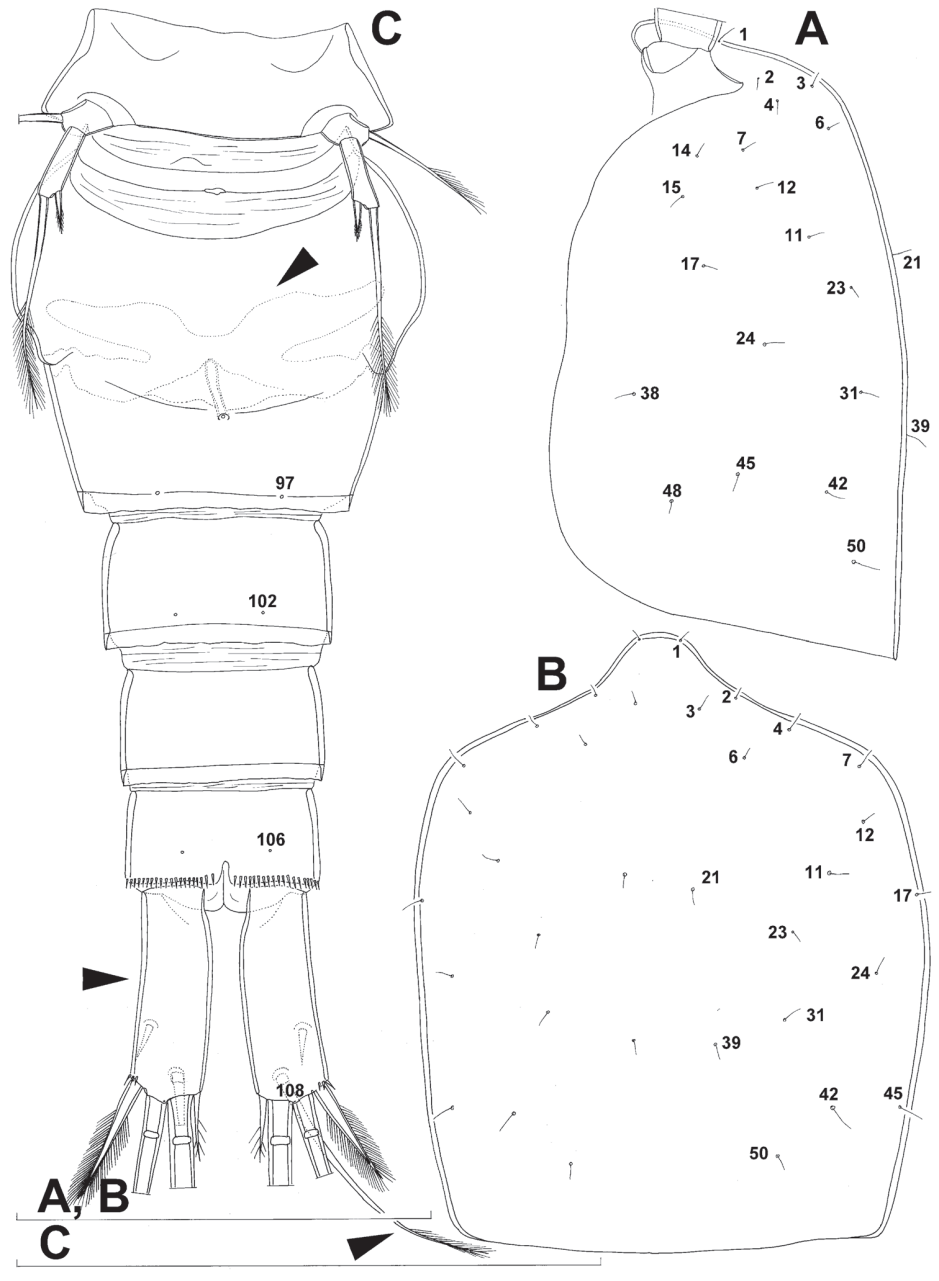
*Diacyclops parasuoensis* sp. n., holotype female: **A** cephalothoracic shield, lateral view **B** cephalothorax, dorsal view **C** urosome, ventral view. Arabic numerals indicating sensilla and pores presumably homologous to those in *Diacyclops ishidai* sp. n. Arrows pointing most prominent specific features. Scale bars 100 μm.

Female (based on holotype and five paratypes from type locality). Total body length, measured from tip of rostrum to posterior margin of caudal rami (excluding caudal setae), from 437 to 462 µm (444 µm in holotype). Preserved specimens colourless; no live specimens observed. Integument relatively weakly sclerotised, smooth, without cuticular pits or cuticular windows. Surface ornamentation of somites consisting of 40 pairs and three unpaired (mid-dorsal) pores and sensilla (those probably homologous with those of *Diacyclops ishidai* sp. n.indicated with same Arabic numerals; presumably novel pairs indicated with Roman numbers and numbered consecutively from anterior to posterior end of body, and from dorsal to ventral side in Figs 9A, B, 10A, B, C); no spinules except on anal somite, caudal rami, and appendages. Habitus (Fig. 9A, B) relatively slender, not dorso-ventrally compressed, with prosome/urosome length ratio 1.5 and greatest width in dorsal view at first third of cephalothorax, body prominently arched backwards between prosome and urosome. Body length/width ratio about 3.5 (dorsal view); cephalothorax 1.86 times as wide as genital double-somite. Free pedigerous somites without lateral or dorsal expansions, all connected by well developed arthrodial membranes, and all with narrow and smooth hyaline fringes. Pleural areas of cephalothorax and free pedigerous somites very short, not covering insertions of cephalic appendages or praecoxae of swimming legs in lateral view.

Rostrum (Fig. 10A, B) well developed, membranous, not demarcated at base, broadly rounded and furnished with one pair of frontal sensilla (no. 1).

Cephalothorax (Figs 9A, B, 10A, B) relatively small, 1.2 times as long as its greatest width (dorsal view), narrower at posterior end in dorsal view, with widest part at first third, only slightly oval; representing 34% of total body length (together with rostrum). Surface of cephalic shield ornamented with 20 pairs of long sensilla (nos. 2-4, 6, 7, 11, 12, 14, 15, 17, 21, 23, 24, 31, 38, 39, 42, 45, 48, 50); no pores visible; sensilla 39–50 belonging to first pedigerous somite, latter being incorporated into cephalothorax.

Second pedigerous somite (Fig. 9A, B) relatively short, tapering anteriorly, ornamented with just one pair of dorsal sensilla (no. 61), serially homologous to pair no. 50 on first pedigerous somite.

Third pedigerous somite (Fig. 9A, B) slightly longer than second and significantly narrower in dorsal view, also widest at posterior margin in dorsal view and with slightly flared latero-posterior corners, ornamented with one unpaired dorsal pore (no. I) and four pairs of large sensilla (nos. 63, 64, 72, 74); recognition of serially homologous pairs not easy, but probably dorsolateral pair of sensilla no. 64 serially homologous to pair no. 61 on second pedigerous somite.

Fourth pedigerous somite (Fig. 9A, B) significantly shorter and narrower than third, with slightly flared latero-posterior corners, ornamented with only one unpaired dorsal pore (no. II) and two pairs of large sensilla (nos. 75, 77); recognition of serially homologous pairs slightly easier than for two previous prosomites (probably II=I, 75=63, 77=72).

Fifth pedigerous somite (Figs 9A, B, 10C) short, significantly narrower than fourth pedigerous somite or genital double-somite in dorsal view, ornamented with one unpaired dorsa pore (no. III) and two pairs of large dorsal sensilla (nos. 80, 81); recognising serially homologous pairs relatively easy, i.e. III=II, 80=75 and 81=77; hyaline fringe very narrow and smooth.

Genital double-somite (Figs 9A, B, 10C, 26E) large, with deep lateral recesses at level of sixth legs and only slightly swollen antero-ventrally, widest at first third of its length and gradually tapering posteriorly, only slightly longer than its greatest width (dorsal view); ornamented with two pairs of central dorsal sensilla (nos. 86, 88), one pair of posterior lateral sensilla (no. 96), and one pair of ventral posterior pores (no. 97); central dorsal sensilla probably serially homologous to those on fifth pedigerous somite (i.e. 86=80, 88=81), but posterior sensilla and pores without homologous pairs; three minute lateral pores posterior to sixth legs visible only under highest magnification of SEM (Fig. 26E). Copulatory pore small, ovoid, situated ventrally at about two-thirds of genital double-somite length; copulatory duct narrow, directed anteriorly, well sclerotised. Hyaline fringe wavy, not serrated. Seminal receptacle butterfly-shaped, with relatively short anterior expansion and long lateral arms, constricted at middle, and with even shorter and slightly narrower posterior expansion, together representing 30% of double-somite’s length; Ovipores situated dorso-laterally at first third of double-somite length, covered by reduced sixth legs.

Third urosomite (Figs 9A, B, 10C) relatively short, about 1.8 times as wide as long and 0.35 times as long as genital double-somite in dorsal view, also with wavy hyaline fringe, ornamented with one pair of lateral posterior sensilla (no. 100) and one pair of ventral posterior pores (no. 102); serially homologous pores and sensilla easy to recognize, i.e. 100=96 and 102=97.

Preanal urosomite (Figs 9A, B, 10C, 26D) slightly narrower and shorter than third, also with wavy hyaline fringe, unornamented.

Anal somite (Figs 9A, B, 10C, 26D) slightly narrower and significantly shorter than preanal somite, with short medial cleft; ornamented with one pair of large dorsal sensilla (no. 104), one pair of small ventral pores (no. 106), and continous posterior row of small spinules. Anal sinus smooth. Anal operculum wide, short, convex, not reaching posterior margin of anal somite, representing 57% of anal somite’s width.

Caudal rami (Figs 9A, B, 10C, 26D) almost cylindrical, slightly divergent, inserted close to each other (space between them about half as wide as one ramus), with deep dorso-median anterior depression (as continuation of anal sinus and narrowed ventral part of base); approximately 2.9 times as long as wide (ventral view) and 2.3 times as long as anal somite, with long dorsal seta (arrowed in Fig. 10C) and longer rami than in *Diacyclops ishidai* or *Diacyclops brevifurcus* (arrowed in Fig. 10C); armed with six setae (one dorsal, one lateral, and four terminal); ornamented with one pore on tip of small protuberance on distal margin ventrally between two principal terminal setae (no. 108), and rows of small spinules at base of lateral setae. Dorsal seta slender, about 1.3 times as long as ramus, inserted at 5/6 of ramus length, biarticulate at base (inserted on small pseudo-joint) and pinnate distally. Lateral seta inserted dorso-laterally at 2/3 of ramus length, about as long as ramus width, sparsely unipinnate laterally and uniarticulate at base. Outermost terminal seta stout, spiniform, 0.6 times as long as ramus, densely bipinnate. Innermost terminal seta small and slender, sparsely pinnate, half as long as outermost terminal seta. Principal terminal setae with breaking planes, bipinnate; inner principal terminal seta about 1.9 times as long as outer one and 4.7 times as long as caudal rami.

Antennula (Fig. 9C) 11-segmented, slightly curved along caudal margin, directed laterally, not reaching posterior margin of cephalothoracic shield, ornamented just with arched proximo-ventral row of spinules on first segment (no pits or other integumental structures), with armature formula as in *Diacyclops ishidai*. Only one terminal seta on ultimate segment biarticulate basally, and most larger setae sparsely pinnate at distal end; both aesthetascs very slender, that on eighth segment reaching posterior margin of tenth segment (arrowed in Fig. 9C). One seta on fourth and one on fifth segment spiniform and short; all other setae slender; one apical seta on eleventh segment fused basally to aesthetasc. Length ratio of antennular segments, from proximal end and along caudal margin, 1 : 0.5 : 0.9 : 0.5 : 0.4 : 0.7 : 1.3 : 1.1 : 0.7 : 0.9 : 1.1.

Antenna (Fig. 9D) five-segmented, strongly curved along caudal margin, comprising very short coxa, much longer basis, and three-segmented endopod. Coxa without armature or ornamentation, about half as long as wide. Basis cylindrical, 2.1 times as long as wide, ornamented with four short, diagonal rows of spinules (two on ventral side, two on dorsal surface) close to caudal margin, armed with two subequal smooth setae on distal inner corner (exopodal seta absent). First endopodal segment narrowed basally but generally cylindrical, 1.6 times as long as wide and 0.7 times as long as basis, with smooth inner seta at 2/3 length and patch of large spinules on caudal margin. Second endopodal segment also with narrowed basal part, 2.3 times as long as wide, about 1.1 times as long as first endopodal segment, bearing only six smooth setae along inner margin (arrowed in Fig. 9D), ornamented with one row of spinules along caudal margin. Third endopodal segment cylindrical, 2.2 times as long as wide and slightly shorter than second endopodal segment, ornamented with two rows of slender spinules along caudal margin, armed with seven smooth apical setae (four of them strong and geniculate).

**Figure 11. F11:**
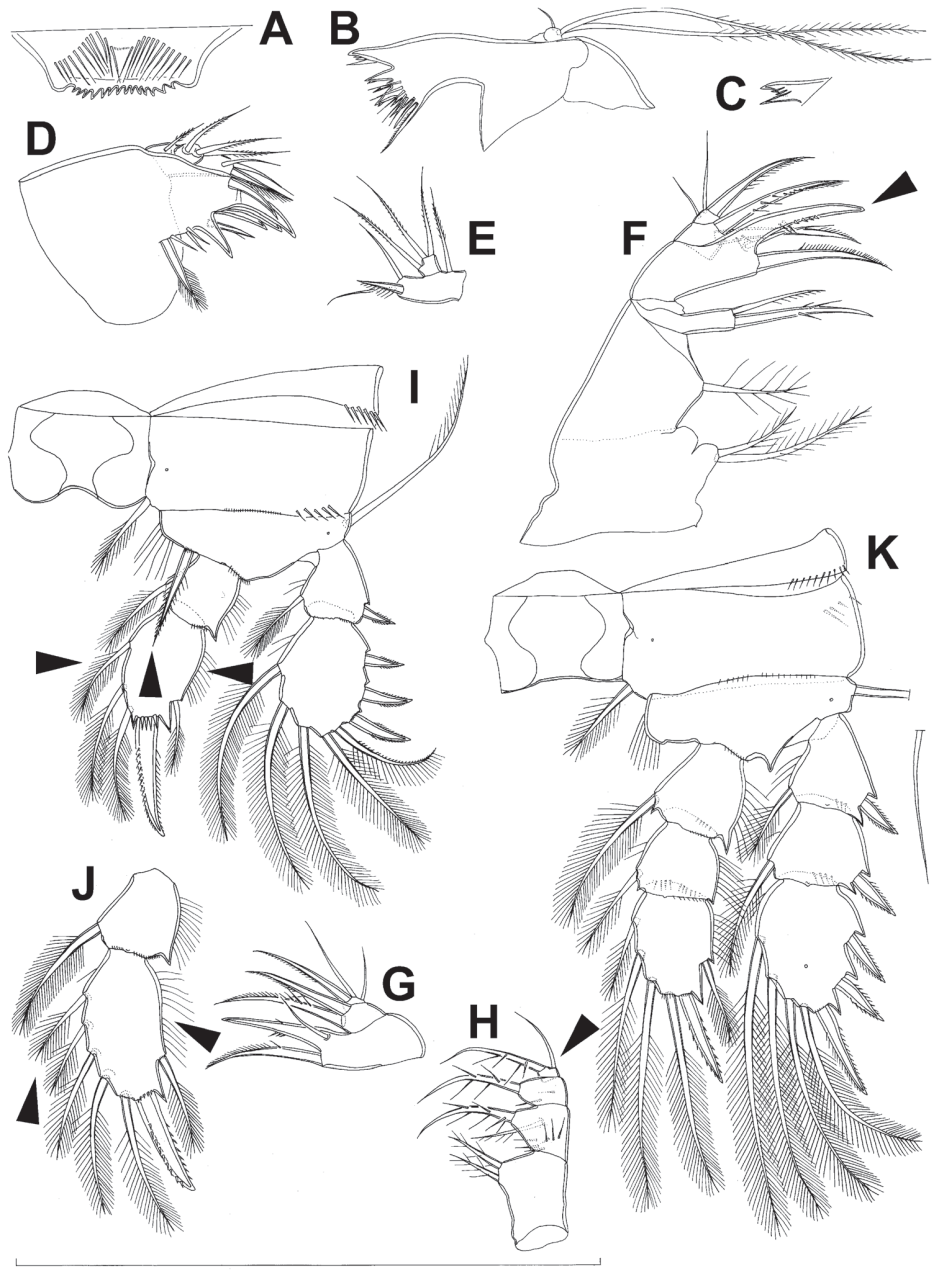
*Diacyclops parasuoensis* sp. n., holotype female: **A** labrum, anterior view **B** mandibula, anterior view **C** quadricuspidate ventralmost tooth of mandibula, posterior view **D** maxillula, posterior view **E** maxillular palp, anterior view **F** maxilla, anterior view **G** basis and endopod of maxilla, posterior view **H** maxilliped, posterior view **I** first swimming leg, anterior view **J** endopod of second swimming leg, anterior view **K** third swimming leg, anterior view. Arrows pointing most prominent specific features. Scale bar 100 μm.

Labrum (Fig. 11A) relatively large trapezoidal plate, with muscular base and strongly sclerotised distal margin (cutting edge), ornamented with two diagonal rows of 12 long and slender spinules each on anterior surface, and central transverse row of minute spinules between them. Cutting edge almost straight, with 14 sharp teeth between produced and rounded lateral corners.

Mandibula (Fig. 11B, C) composed of coxa and small palp. Coxal gnathobase cutting edge with five slender spinules on anterior surface, seven apical teeth, and dorsalmost unipinnate seta; ventralmost tooth strongest and quadricuspidate (Fig. 11C), second and fourth teeth from ventral side bicuspidate, all other teeth unicuspidate; two dorsalmost simple teeth partly fused basally. Palp twice as wide as long, unornamented, armed with three apical setae: two long and bipinnate and one short and smooth; pinnate setae subequal in length, directed posteriorly, not reaching posterior margin of cephalic shield (see also Fig. 9B).

Maxillula (Fig. 11D, E) composed of praecoxa and two-segmented palp, unornamented. Praecoxal arthrite bearing four very strong distal spines (three of them smooth, blunt, and fused at base; one distinct at base, sharp, and with two proximal spinules) and six medial elements (proximalmost one longest and plumose, two distalmost ones large and strong, three in between small and slender). Palp composed of coxobasis and one-segmented endopod. Coxobasis with slender, bipinnate proximal seta (probably representing exopod) and three medial setae (two slender and smooth, one strong and pinnate). Endopod with three slender, bipinnate setae.

Maxilla (Fig. 11F, G) 5-segmented but praecoxa partly fused to coxa on anterior surface, unornamented. Proximal endite of praecoxa robust, armed with two sparsely bipinnate setae; distal endite slightly smaller than proximal and unarmed. Proximal endite of coxa with one bipinnate seta; distal endite highly mobile, elongated and armed apically with two pinnate setae, proximal one considerably longer and stronger. Basis armed with two setae, expanded into robust claw. Claw shorter than larger seta and furnished with longitudinal row of six spinules at midlength along concave (dorsal) margin (arrowed in Fig. 11F); larger seta with convex ventral margin, robust and spiniform; smaller seta smooth and slender, inserted on posterior surface. Endopod two-segmented with segmentation easily discernable; proximal segment armed with two robust, unipinnate setae; distal segment with one robust, unipinnate apical seta and two slender and much shorter subapical setae. Longest seta on distal endopodal segment 0.8 times as long as longer seta on proximal endopodal segment. All strong setae on basis and endopod, as well as basal claw, unguiculate.

Maxilliped (Fig. 11H) four-segmented, composed of syncoxa, basis, and two-segmented endopod; second endopodal segment minute, armed with only two setae (arrowed in Fig. 11H). Ornamentation consisting of two rows of long, slender spinules on basis (one on posterior surface, other on anterior surface), as well as two spinules on anterior surface of first endopodal segment. Armature formula: 2.2.1.2. All inner setae pinnate, very strong, and those on basis and endopod also unguiculate.

**Figure 12. F12:**
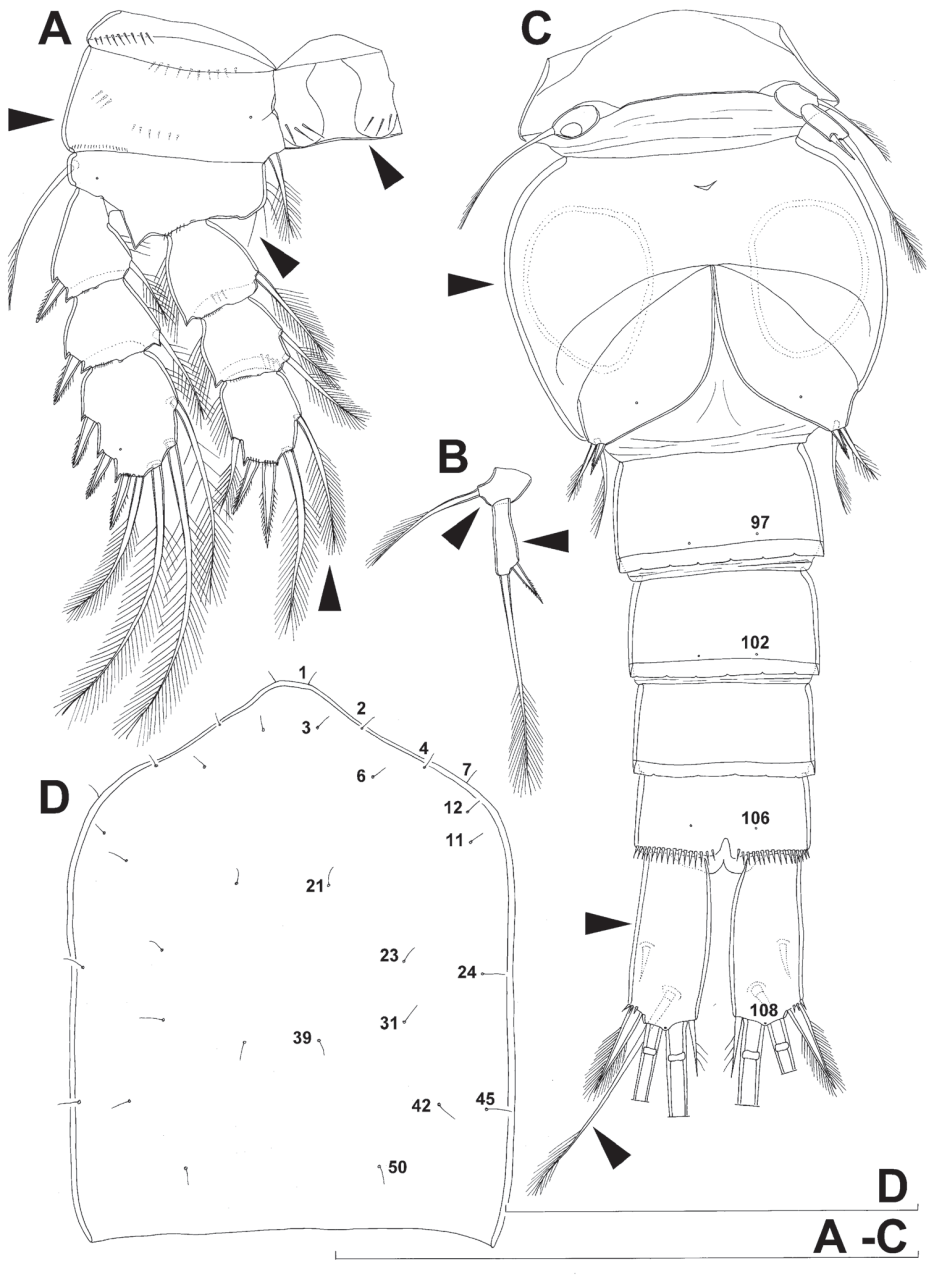
*Diacyclops parasuoensis* sp. n., **A–B** holotype female **C–D** allotype male. **A** fourth swimming leg, anterior view **B** fifth leg, anterior view **C** urosome, ventral view **D** cephalothorax, dorsal view. Arabic numerals indicating sensilla and pores presumably homologous to those in *Diacyclops ishidai* sp. n. Arrows pointing most prominent specific features. Scale bars 100 μm.

All swimming legs (Figs 11I, J, K, 12A) relatively small, composed of minute, triangular praecoxa, large, rectangular coxa, short basis, and slender exopod and endopod. Exopods and endopods approximately equally long on all legs, their segmentation formula (exopod/endopod): 2/2.3/2.3/3.3/3. Ultimate exopodal segment spine formula 3.3.3.3 and setal formula 5.4.4.4. All setae on endopods and exopods slender and plumose, except apical seta on exopod of first leg pinnate along outer margin and plumose along inner (Fig. 11I); no modified setae observed. All spines strong and bipinnate. Intercoxal sclerite of all swimming legs with slightly concave distal margin and without any surface ornamentation except on anterior surface of fourth leg.

First swimming leg (Fig. 11I) shorter than other swimming legs; praecoxa unarmed, ornamented with distal row of large spinules on anterior surface; coxa 2.3 times as wide as long, ornamented with distal row of spinules on anterior surface (outer half of row composed of large spinules, inner half of minute spinules), and small pore on anterior surface close to inner margin, armed with slender and sparsely plumose seta on inner-distal corner; basis almost pentagonal, 0.7 times as long as coxa, armed with long and slender outer seta and strong and bipinnate inner-distal element (latter reaching to 1/3 length of second endopodal segment, i.e. much shorter than in *Diacyclops ishidai*; arrowed in Fig. 11I), ornamented with row of extremely slender spinules along inner margin, two posterior rows of minute spinules on anterior surface (one at base of inner seta, other at base of endopod), and one cuticular pore on anterior surface close to outer margin; exopod with single outer spine and single inner seta on first segment, with three outer spines and five setae (three inner, two apical) on second segment, ornamented with distal rows of spinules on anterior surface of first segment, row of slender inner spinules on both segments, and extremely minute spinules at base of almost all setae and spines on anterior surface; endopod armed only with inner seta on first segment, second segment with only three inner setae (arrowed in Fig. 11I), one apical spine, and one outer seta, ornamented with slender spinules along inner margins of both segments, with shorter and stronger spinules along distal margin of first segment on anterior surface, and with minute spinules at base of most setae (those at base of apical spine larger) on anterior surface; apical spine on second endopodal segment slightly outwardly unguiculate, and only slightly longer than segment or inner setae; second endopodal segment about 1.5 times as long as wide and also 1.7 times as long as first endopodal segment, lacking inner notch (arrowed in Fig. 11I).

Second swimming leg (Fig. 11J) similar to that of *Diacyclops ishidai*, but second endopodal segment without inner notch showing ancestral segmentation (arrowed in Fig. 11J) and with proximal two setae shorter than others (arrowed in Fig. 1J); apical spine on second endopodal segment proportionally longer than in *Diacyclops ishidai*, 0.7 times as long as segment or distal inner seta; second endopodal segment about 1.9 times as long as wide and 1.8 times as long as first endopodal segment.

Third swimming leg (Fig. 11K) similar to that of *Diacyclops ishidai*, but third endopodal segment proportionally shorter, and third exopodal segment with pore on anterior surface; apical spine on third endopodal segment as long as segment and half as long as apical seta; third endopodal segment about 1.2 times as long as wide and 1.4 times as long as second endopodal segment.

Fourth swimming leg (Fig. 12A) generally similar to that of *Diacyclops ishidai*, but coxa with fewer spinules on posterior surface (arrowed in Fig. 12A), intercoxal sclerite without spinules on posterior surface but instead with six large spinules on anterior surface (arrowed in Fig. 12A), inner process of basis much smaller (arrowed in Fig. 12A), and proximal inner seta on third endopodal segment much shorter (Fig. 12A); third endopodal segment without pore on anterior surface, about 1.2 times as long as wide, and 1.2 times as long as second endopodal segment; inner apical spine on third endopodal segment 1.5 times as long as outer apical spine, slightly shorter than segment, and less than half as long as distal inner seta; apical spines diverging at about 20° angle.

Fifth leg (Figs 10C, 12B) inserted ventrally, relatively small, two-segmented, with same armature as in *Diacyclops ishidai* and *Diacyclops brevifurcus*, but with very different shape and proportions. Protopod very small and narrow (arrowed in Fig. 12B), almost trapezoidal, about as long as greatest width, unornamented, armed with single slender outer seta inserted on short setophore and unipinnate distally. Exopod slightly narrower than in these congeners, almost cylindrical but with narrower proximal part, twice as long as protopod and 3.2 times as long as wide, unornamented, armed with long apical seta and subapical inner spine; apical seta bipinnate distally, 1.7 times as long as basal seta, 2.9 times as long as exopod, and 4.7 times as long as subapical spine, reaching to 2/3 length of genital double-somite; subapical exopodal spine small but strong, bipinnate, 0.63 times as long as exopod and twice as long as exopod’s greatest width.

Sixth leg (Figs 9A, B, 26E) small, short, and broad semicircular cuticular plate with single pore on anterior surface, two short and smooth spines, and one longer and distally unipinnate outermost seta; inner spine fused to plate, outer one articulated basally; outermost seta directed postero-dorsally.

**Figure 13.**
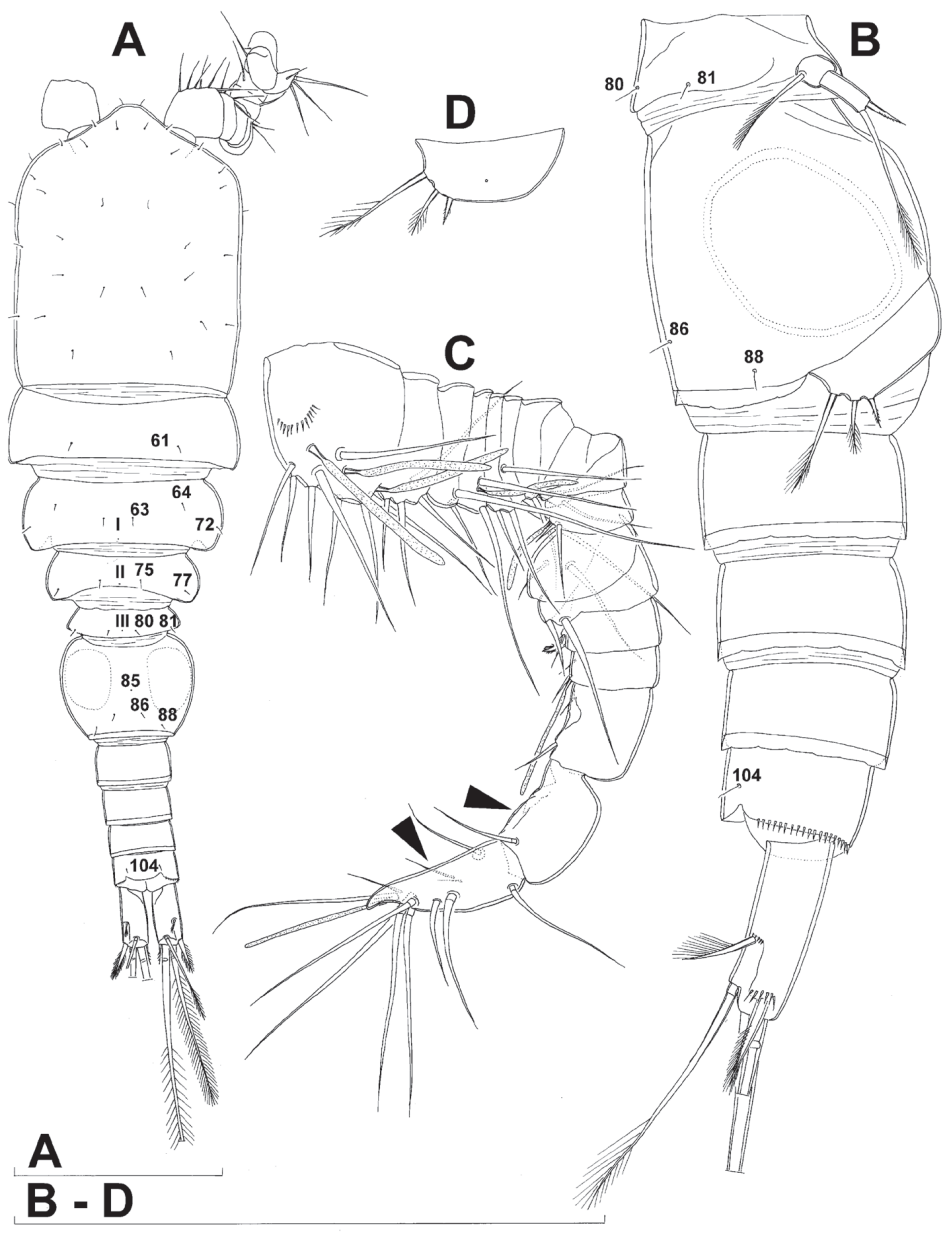
*Diacyclops parasuoensis* sp. n., allotype male: **A** habitus, dorsal view **B** urosome, lateral view **C** antennula, flattened and slightly uncoiled, ventral view **D** sixth leg, ventro-lateral view. Arabic numerals indicating sensilla and pores presumably homologous to those in *Diacyclops ishidai* sp. n. Roman numerals indicating pores not present in *Diacyclops ishidai* sp. n. Arrows pointing most prominent specific features. Scale bars 100 μm.

Male (based on allotype and four paratypes from type locality). Total body length 402–437 µm (406 µm in allotype). Urosome with free genital somite. Habitus (Fig. 13A) even more slender than in female, with prosome/urosome length ratio about 1.5 and greatest width in dorsal view at second pedigerous somite. Body length/width ratio 3.6; cephalothorax about 1.7 times as wide as genital somite. Cephalothorax 1.2 times as long as wide and nearly cylindrical in dorsal view, representing 33% of total body length. Ornamentation of cephalothorax (Fig. 12D), free prosomites (Fig. 13A), and first and last two urosomites (Figs 12C, 13A, B) with same number and distribution of sensilla and pores as in female.

Genital somite (Figs 12C, 13A, B) 1.3 times as wide as long in dorsal view, with wavy hyaline fringe dorsally, ornamented with one unpaired dorsal pore (no. 85; N.B., this pore absent in female) and two pairs of dorsal and lateral sensilla (nos. 86, 88); two small circular spermatophores visible inside. Third urosomite (Figs 12C, 13A, B) homologous to posterior part of female genital double-somite, also ornamented with ventral pair of posterior pores (no. 97), but without lateral pair of sensilla (no. 96). Fourth urosomite (Figs 12C, 13A, B) also lacking lateral pair of sensilla present in female (no. 100), only ornamented with ventral posterior pair of pores (no. 102).

Caudal rami (Figs 12C, 13A, B) slightly shorter than in female and less divergent, but nonetheless with long dorsal seta (arrowed in Fig. 12C) and longer rami than in *Diacyclops ishidai* or *Diacyclops brevifurcus* (arrowed in Fig. 12C), with proportionally shorter outermost terminal seta than in female, but with very similar ornamentation and armature.

Antennula (Fig. 13C) strongly prehensile and digeniculate, 16-segmented (with ancestral sixteenth and seventeenth segments completely fused), ornamented with spinules only on first segment (as in female), with anvil-shaped cuticular ridges on anterior margin of fourteenth and fifteenth segments (distal geniculation), with much shorter fifteenth and sixteenth segments than in *Diacyclops ishidai* (both arrowed in Fig. 13C), and also fifteenth segment without aesthetasc and sixteenth segment with one additional minute seta. Armature formula as follows: 8+3ae.4.2.2+ae.2.2.2.2.2+ae.2.2.2.2 + ae.2.1.12+ae. All aesthetascs linguiform and most relatively slender, apical one on sixteenth segment fused basally to one seta; distribution of small setae as in *Diacyclops ishidai*.

Antenna, labrum, mandibula, maxillula, maxilla, swimming legs, and fifth leg as in female.

Sixth leg (Fig. 13B, D) large cuticular plate with single minute pore on anterior surface, armed with small inner spine and two bipinnate setae on outer distal corner; outermost seta 2.3 times as long as inner bipinnate seta, 4.3 times as long as innermost spine.

##### Remarks.

**Figure 14.**
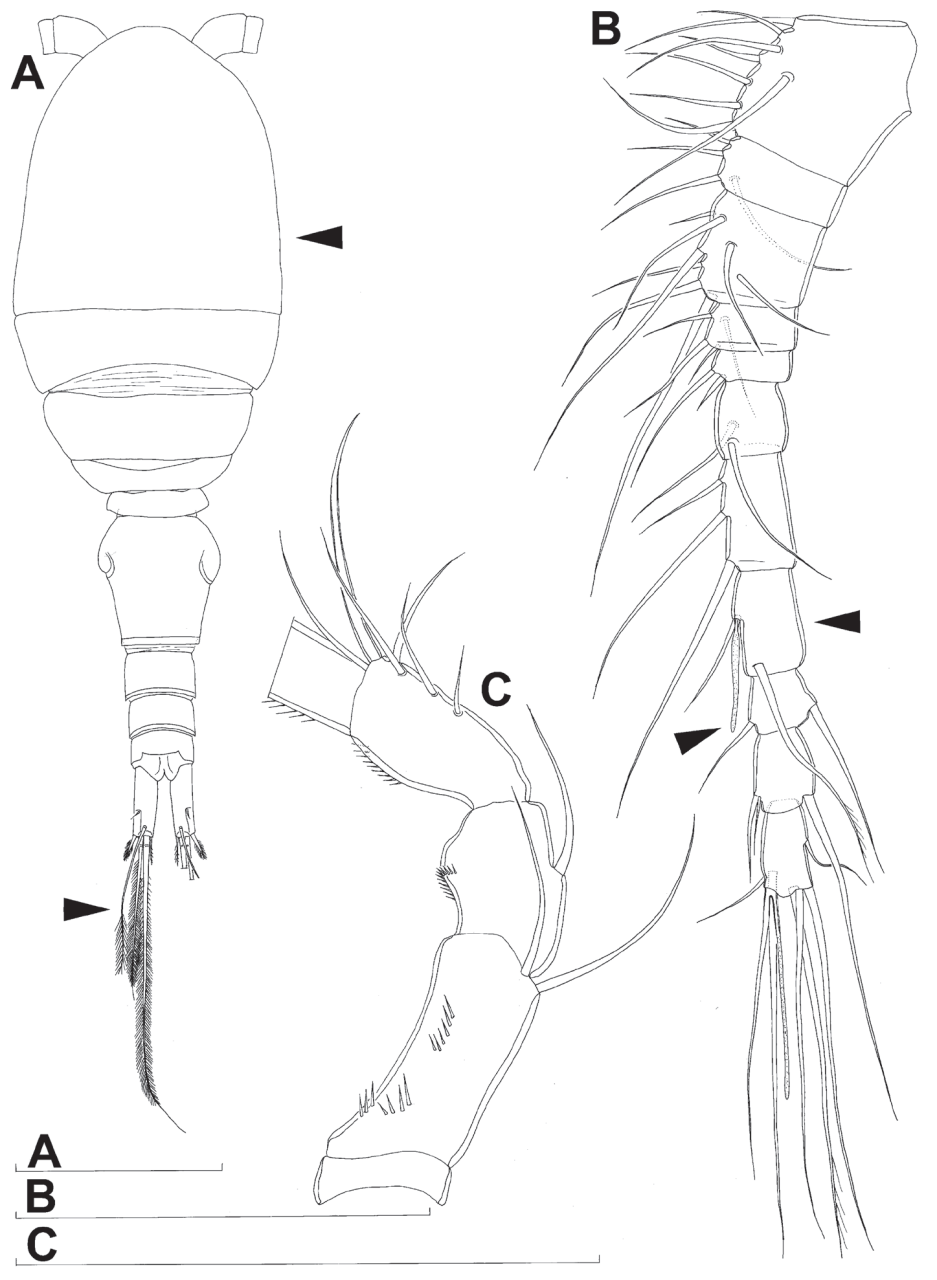
*Diacyclops suoensis* Ito, 1954, female: **A** habitus, dorsal view **B** antennula, ventral view **C** antenna, ventral view. Arrows pointing most prominent specific features. Scale bars 100 μm.

**Figure 15. F15:**
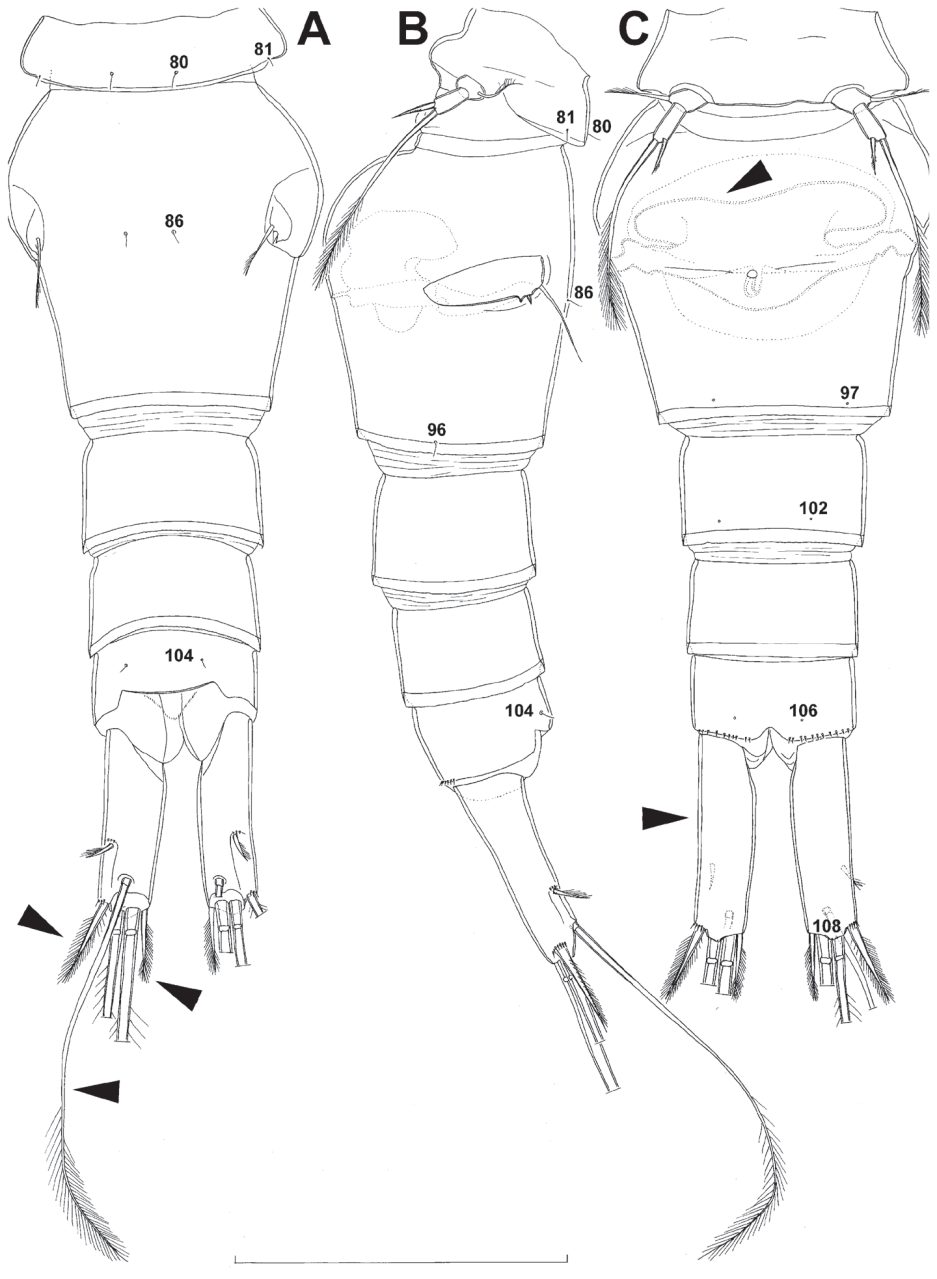
*Diacyclops suoensis* Ito, 1954, female: **A** urosome, dorsal view **B** urosome, lateral view **C** urosome, ventral view. Arabic numerals indicating sensilla and pores presumably homologous to those in *Diacyclops ishidai* sp. n. Arrows pointing most prominent specific features. Scale bar 100 μm.

**Figure 16. F16:**
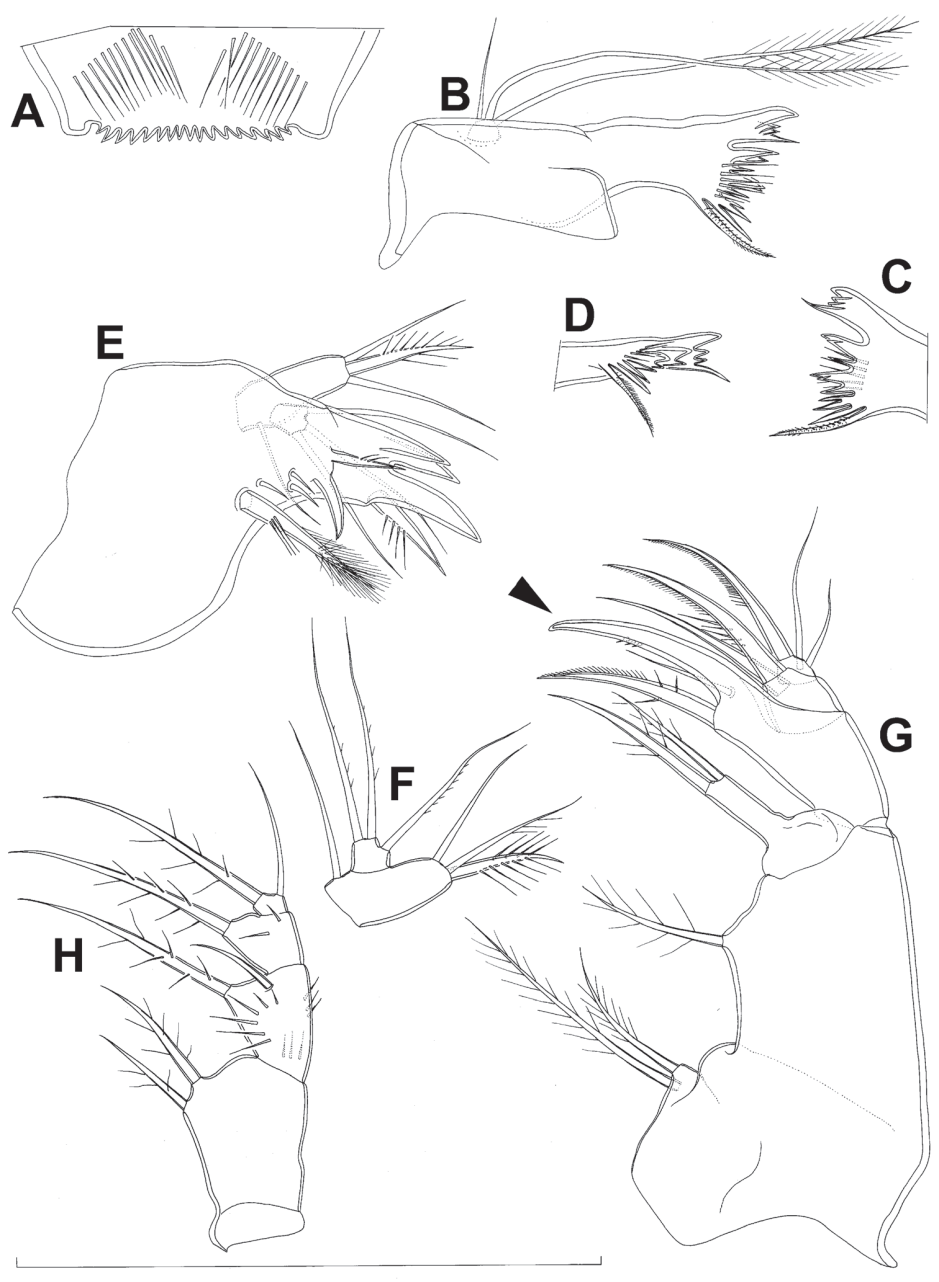
*Diacyclops suoensis* Ito, 1954, female: **A** labrum, anterior view **B** mandibula, anterior view **C** cutting edge of mandibula, posterior view **D** cutting edge of mandibula, dorsal view **E** maxillula, posterior view **F** maxillular palp, posterior view **G** maxilla, anterior view **H** maxilliped, anterior view. Arrows pointing most prominent specific features. Scale bar 100 μm.

**Figure 17.**
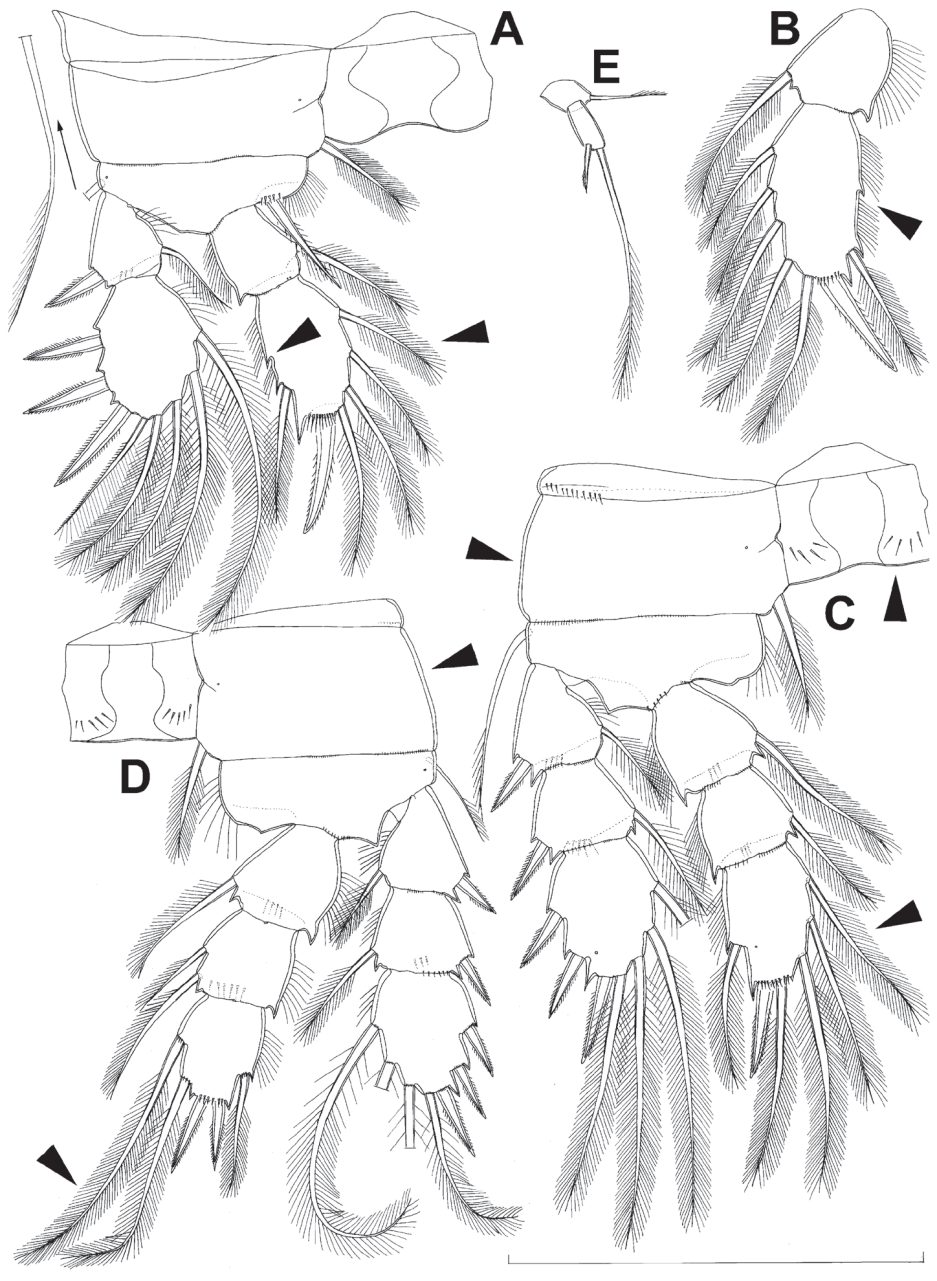
*Diacyclops suoensis* Ito, 1954, female: **A** first swimming leg, anterior view **B** endopod of second swimming leg, anterior view **C** third swimming leg, anterior view **D** fourth swimming leg, anterior view **E** fifth leg, anterior view. Arrows pointing most prominent specific features. Scale bar 100 μm.

*Diacyclops parasuoensis* sp. n. can be easily distinguished from the Japanese *Diacyclops suoensis* and *Diacyclops pseudosuoensis* sp. n. by the size of the dorsal caudal seta, as well as by the proportions of many armature elements and the ornamentation of most appendages. Most of these differences are arrowed in Figs 14-17. However, very few differences in the pattern of pores and sensilla and the similar armature of the antenna in *Diacyclops parasuoensis* and *Diacyclops suoensis* probably indicate that these two species are not distantly related, and may form a monophyletic group together with *Diacyclops pseudosuoensis* and *Diacyclops hisuta* sp. n. They are all only remotely related to *Diacyclops ishidai* sp. n., *Diacyclops brevifurcus*, *Diacyclops leeae* sp. n., *Diacyclops hanguk* sp. n., and *Diacyclops parahanguk* sp. n.

It is quite clear that *Diacyclops parasuoensis* forms a sibling species pair with the Japanese *Diacyclops hisuta* (see below), and they can only be distinguished at this stage by the habitus shape (much more slender in *Diacyclops parasuoensis*). We described *Diacyclops hisuta* for a population reported and partly described by Ueda et al. (1996) under the name *Diacyclops suoensis* (see synonymy below). Unfortunately, the description and illustrations they provide do not show details of any mouth appendages or the armature of the first three swimming legs, so many characters cannot be compared. If we assume these features are very similar to those of *Diacyclops suoensis* as illustrated by Ito (1957), who provided drawings of all swimming legs, *Diacyclops hisuta* would differ from *Diacyclops parasuoensis* additionally by the number of inner setae on the second endopodal segment of the first leg. This, however, remains speculative until *Diacyclops hisuta* is properly redescribed.

A great number of species (and subspecies) in the *languidoides*-group have been described based on a very limited set of characters, giving us no opportunity to compare fine details of the somite sensilla and pores pattern or the appendages ornamentation. This species-group was for a long time considered a single widely-distributed species, although some authors noticed a high level of morphological variability even in a small geographic area (Ito 1954; Kiefer 1968). Even today, some authors consider as valid no fewer than 14 subspecies (see Dussart and Defaye 2006), many of them with overlapping ranges. It was Petkovski (1984) who first realized that most of these subspecies must be distinct biological species, after he found three of them occurring sympatrically in a single interstitial sample from Slovenia. He illustrated their major differences in caudal rami shape and ornamentation, shape of the genital double-somite and its seminal receptacle, and proportions of the third endopodal segment of the fourth leg and its apical spines. Since then these particular characters have normally been illustrated for new, presumably closely related taxa, while other features have not been studied in detail. Thus, most comparisons between different taxa in this species-group have to be inferred today from these three characters, especially the shape and armature of the caudal rami. Somewhat similar caudal rami to those of *Diacyclops parasuoensis* are found in the following European species: *Diacyclops clandestinus* (Kiefer, 1926), *Diacyclops cristinae* Pesce & Galassi, 1987, *Diacyclops eriophori* (Gurney, 1927), *Diacyclops hypnicola* (Gurney, 1927), *Diacyclops insularis* Monchenko, 1982, and *Diacyclops paolae* Pesce & Galassi, 1987 (see Gurney 1927; Herbst 1951; Kiefer 1968; Pesce and Maggi 1981; Monchenko 1982; Petkovski 1984; Pesce and Galassi 1987a, b; Galassi 1991). In all of these species, some small differences from *Diacyclops parasuoensis* can, however, be discerned in the proportions and armature of the caudal rami. Also, none of them has such elongated lateral arms of the anterior part of seminal receptacle, and most also differ from *Diacyclops parasuoensis* in some other significant morphological characters. For example, *Diacyclops insularis* has eight setae on the second endopodal segment of the antenna, and *Diacyclops cristinae* and *Diacyclops hypnicola* have an exopodal seta on the antenna.

#### Diacyclops suoensis

Ito, 1954

http://species-id.net/wiki/Diacyclops_suoensis

Figs 14 [Fig F15] [Fig F16] 17 26F

Diacyclops languidoides suoensis n. subsp. – Ito 1954: p. 399, Figs 114–148. Synonymy.
Diacyclops languidoides suoensis Ito – Ito 1957: p. 15, fig. 45. [partim.]
Diacyclops languidoides suoensis Ito – Ito 1957: p. 15, Figs 35–44, 46–48. [non]
Diacyclops suoensis Ito – Ueda et al. 1996: p. 309, fig. 4.; Lee et al. 2007: p. 162, Figs 7–8; Chang 2009: p. 478, Figs 263–264. [non]

##### Type locality.

Japan, Yamaguchi prefecture, Tsunoshima island, Tsuno city, approximately 34°21'N, 130°52'E, well with a pump.

##### Material examined.

One female dissected on two slides (LBM1430005385), two females dissected on one slide each (LBM1430005386 & LBM1430005387), two females on one slide in toto (LBM1430005388), and three females on one SEM stub (LBM1430005389); all collected from Japan, Shiga prefecture, Otsu city, Nakano 3-chome district, Daido River, 34°57.043'N, 135°57.044'E, interstitial water from medium to coarse sand, 27 September 2009, leg. T. Karanovic.

Additional two females in ethanol (LBM1430005390) from Japan, Shiga prefecture, Lake Biwa, Otsu city, Arakawa district, Matsunoura Beach, lake beach next to mouth of swift-flowing irrigation runoff canal, 35°12.319'N, 135°55.768'E, interstitial water from medium to coarse sand, 4 October 2009, leg. T. Karanovic.

##### Redescription.

Female (based on eight specimens from Daido River). Total body length, measured from tip of rostrum to posterior margin of caudal rami (excluding caudal setae), from 391 to 473 µm (391 µm in holotype). Preserved specimens colourless; no live specimens observed. Integument relatively weakly sclerotised, smooth, without cuticular pits or cuticular windows. Surface ornamentation of somites very similar to that of *Diacyclops parasuoensis*, consisting of 38 pairs of pores and sensilla (those probably homologous with those of *Diacyclops ishidai* indicated with same Arabic numerals in Fig. 15A, B, C); no spinules except on anal somite, caudal rami, and appendages. Habitus (Fig. 14A) relatively robust, not dorso-ventrally compressed, with prosome/urosome ratio 1.3 and greatest width in dorsal view at posterior end of cephalothorax, only slightly arched backwards between prosome and urosome. Body length/width ratio about three (dorsal view); cephalothorax 2.1 times as wide as genital double-somite. Free pedigerous somites without lateral or dorsal expansions, all connected with well developed arthrodial membranes, and with narrow and smooth hyaline fringes, but arthrodial membranes not as exposed as in *Diacyclops parasuoensis*. Pleural areas of cephalothorax and free pedigerous somites short, not covering insertions of cephalic appendages or praecoxae of swimming legs in lateral view.

Rostrum, cephalothorax, and three free pedigerous somites ornamented as in *Diacyclops parasuoensis*, except dorsal unpaired pores (nos. I, II) absent.

Cephalothorax (Fig. 14A) relatively large (arrowed in Fig. 14A), 1.1 times as long as its greatest width (dorsal view), widest at posterior end in dorsal view and tapering towards anteriorly, oval; representing 35% of total body length (together with rostrum). Surface of cephalic shield ornamented as in *Diacyclops parasuoensis* with 20 pairs of long sensilla (nos. 2-4, 6, 7, 11, 12, 14, 15, 17, 21, 23, 24, 31, 38, 39, 42, 45, 48, 50); no pores visible; sensilla 39-50 belonging to first pedigerous somite, incorporated into cephalothorax.

Second pedigerous somite (Figs 14A) relatively short, tapering posteriorly, ornamented with just one pair of dorsal sensilla (no. 61) as in *Diacyclops parasuoensis*.

Third pedigerous somite (Fig. 14A) slightly longer than second and significantly narrower in dorsal view, also tapering posteriorly, ornamented with four pairs of large sensilla (nos. 63, 64, 72, 74); unpaired dorsal pore (no. I) absent.

Fourth pedigerous somite (Figs 14A) significantly shorter and narrower than third, tapering posteriorly, ornamented with only two pairs of large sensilla (nos. 75, 77); unpaired dorsal pore (no. II) absent.

Fifth pedigerous somite (Fig. 15A, B, C) short, significantly narrower than fourth pedigerous somite or genital double-somite in dorsal view, ornamented with two pairs of large dorsal sensilla (nos. 80, 81); unpaired dorsal pore (no. III) absent.

Genital double-somite (Fig. 15A, B, C) large, slightly more slender than in *Diacyclops parasuoensis*, with deep lateral recesses at level of sixth legs and swollen antero-ventrally, widest at first third of its length and gradually tapering posteriorly, as long as its greatest width (dorsal view); ornamented with one pair of central dorsal sensilla (no. 86), one pair of posterior lateral sensilla (no. 96), and one pair of ventral posterior pores (no. 97); central dorsal pair of sensilla probably serially homologous to those on fifth pedigerous somite (i.e. 86=80), but posterior sensilla and pores without homologous pairs; dorsal pair of central sensilla no. 88 absent. Copulatory pore small, oval, situated ventrally at about midlenth of double-somite; copulatory duct narrow, siphon-shaped, well sclerotised. Hyaline fringe wavy, not serrated. Seminal receptacle butterfly-shaped, but with much thicker and shorter lateral arms than in *Diacyclops parasuoensis* (arrowed in Fig. 15C); representing 43% of double-somite’s length; oviducts broad and well sclerotised. Ovipores situated dorso-laterally at 2/5 of double-somite length, covered by reduced sixth legs.

Third urosomite (Fig. 15A, B, C) similar in size and shape to that of *Diacyclops parasuoensis*, but lateral pair of sensilla (no. 100) absent, only ornamentation ventral posterior pair of pores (no. 102); hyaline fringe wavy.

Preanal urosomite (Fig. 15A, B, C) as in *Diacyclops parasuoensis*, unornamented and with wavy hyaline fridge.

Anal somite (Fig. 15A, B, C) also similar to that of *Diacyclops parasuoensis*, but with posterior row of spinules limited to ventral surface and composed of much smaller spinules. Anal sinus ornamented with two diagonal rows of minute spinules. Anal operculum wide, short, only slightly convex, not reaching posterior margin of anal somite, representing 54% of anal somite’s width.

Caudal rami (Fig. 14A, 15A, B, C) proportionately longer than in *Diacyclops parasuoensis* (arrowed in Fig. 15C), and with shorter outermost terminal seta (arrowed in Fig. 15A), longer innermost terminal seta (arrowed in Fig. 15A), and much longer dorsal seta (arrowed in Figs 14A, 15A), approximately 3.4 times as long as wide (ventral view) and 2.5 times as long as anal somite; ornamented with one pore on tip of small protuberance on distal margin ventrally between two principal terminal setae (no. 108), and rows of small spinules at base of lateral setae. Dorsal seta slender, about 2.2 times as long as ramus and almost as long as outer principal terminal seta, inserted at 5/6 of ramus length, biarticulate at base (inserted on small pseudo-joint) and bipinnate distally. Lateral seta minute, inserted dorso-laterally at 2/3 of ramus length, about half as long as ramus width, unipinnate laterally and uniarticulate at base. Outermost terminal seta stout, spiniform, 0.4 times as long as ramus, densely bipinnate. Innermost terminal seta more slender and 0.8 as long as outermost terminal one, also densely bipinnate. Principal terminal setae with breaking planes, bipinnate; inner principal terminal seta about 1.8 times as long as outer one and 4.2 times as long as caudal rami.

Antennula (Fig. 14B) unornamented, segmentation and armature as in *Diacyclops parasuoensis* and *Diacyclops ishidai*, except eighth segment proportionatelly longer (arrowed in Fig. 14B) and aesthetasc on eighth segment proportionatelly shorter (reaching posterior margin of ninth segment; arrowed in Fig. 14B). Length ratio of antennular segments, from proximal end and along caudal margin, 1 : 0.5 : 0.9 : 0.5 : 0.4 : 0.7 : 1.2 : 1 : 0.6 : 0.8 : 1.

Antenna (Fig. 14C) with fewer spinules on basis than in *Diacyclops parasuoensis*, but all other ornamentation, as well as segmentation and armature without any difference.

Labrum (Figs 16A, 26F) relatively large trapezoidal plate with mascular base and strongly sclerotised distal margin (cutting edge), ornamented with two diagonal rows of 11 to 13 long and slender spinules on anterior surface; cutting edge almost straight, with 16 to 18 sharp teeth between produced and rounded lateral corners.

Mandibula (Fig. 16B, C, D) very similar to that of *Diacyclops parasuoensis*, but with additional small unicuspidate tooth on cutting edge (part of dorsalmost, partly fused group of three theeth).

Maxillula (Figs 16E, F, 26F) segmentation, armature, and ornamentation as in *Diacyclops parasuoensis*, but free distal spine on praecoxal arthrite more pinnate and endopodal setae less pinnate.

Maxilla (Fig. 16G) segmentation, armature, and ornamentation as in *Diacyclops parasuoensis*, except basal claw slightly longer (arrowed in Fig. 16G), and with only three small spinules on dorsal margin.

Maxilliped (Fig. 16H) with more spinules in anterior row on basis, but other ornamentation, as well as segmentation and armature, as in *Diacyclops parasuoensis*.

All swimming legs (Fig. 17A, B, C, D) generally similar to those in *Diacyclops parasuoensis*; but second endopodal segment of first leg with one additional seta (arrowed in Fig. 17A) and all legs with small differences in ornamentation of some segments and proportions of some setae. Segmentation formula, as well as ultimate exopodal segment spine and setal formulae, as in *Diacyclops parasuoensis*.

First swimming leg (Fig. 17A) without large spinules on coxa and with inner notch on second endopodal segment (arrowed in Fig. 17A), with additional inner seta on second endopodal segment (arrowed in Fig. 17A); apical spine on second endopodal segment slightly outwardly unguiculate, 0.9 times as long as segment, and 0.7 times as long as distal inner seta; second endopodal segment about 1.7 times as long as wide and 1.6 times as long as first endopodal segment.

Second swimming leg (Fig. 17B) without large spinules on posterior surface of coxa and with inner notch on second endopodal segment (arrowed in Fig. 17B); apical spine on second endopodal segment 0.7 times as long as segment or distal inner seta; second endopodal segment about 1.8 times as long as wide and 1.6 times as long as first endopodal segment.

Third swimming leg (Fig. 17C) also without large spinules on posterior surface of coxa (arrowed in Fig. 17C), with two short rows of large spinules on anterior surface of intercoxal sclerite (arrowed in Fig. 17C), and with normally developed proximal seta on third endopodal segment (arrowed in Fig. 17C); apical spine on third endopodal segment 0.7 times as long as segment and less than half as long as apical seta; third endopodal segment with pore on anterior surface, about 1.5 times as long as wide and 1.6 times as long as second endopodal segment.

Fourth swimming leg (Fig. 17D) without large spinules on posterior margin of coxa (arrowed in Fig. 17D), and with proximal inner seta on third endopodal segment longer than distal inner seta (arrowed in Fig. 17D); third endopodal segment about 1.2 times as long as wide, and 1.2 times as long as second endopodal segment; inner apical spine on third endopodal segment 1.2 times as long as outer apical spine, 0.8 times as long as segment, and less than 0.4 times as long as distal inner seta; apical spines diverging at about 20° angle.

Fifth leg (Fig. 17E) very similar to that of *Diacyclops parasuoensis*, but basal seta and exopod proportionally shorter; protopod small and narrow, rhomboidal in shape and about as long as greatest width, unornamented, armed with single outer slender, short seta, this being inserted on very short setophore and unipinnate distally; exopod slightly narrower than protopod, almost cylindrical but with narrowed proximal part, 1.8 times as long as protopod and 2.2 times as long as wide, unornamented, armed with long apical seta and subapical inner spine; apical seta bipinnate distally, 3.3 times as long as basal seta, 4.8 times as long as exopod, and five times as long as subapical spine, reaching to 2/3 length of genital double-somite; subapical exopodal spine only slightly shorter than exopod and twice as long as exopod’s greatest width.

Sixth leg (Fig. 15A, B) as in *Diacyclops parasuoensis*, but with slightly longer outer seta.

Male. Not collected.

##### Remarks.

This record extends the known distribution of *Diacyclops suoensis* more than 290 km eastward. Ito (1954) described it from Tsunoshima island in the Yamaguchi prefecture, and Ito (1957) later reported it from Yoshida in Hiroshima prefecture, some 170 km east-northeast of the type locality. All three localities are on Honshu, which is the largest island in Japan. Ito (1957) also reported and illustrated a population of *Diacyclops suoensis* from Amami-Oshima island, some 650 km south of the type locality. Here we describe this last population as the new species *Diacyclops pseudosuoensis* (see below). Although they form a sibling species pair, the two can be distinguished easily by the relative length of the innermost terminal caudal seta, as well as by the length of the apical spines on the fourth leg endopod. No other species of the *languidoides-*group has such long dorsal caudal setae as *Diacyclops suoensis* or *Diacyclops pseudosuoensis*, except perhaps *Diacyclops pelagonicus* Petkovski, 1971 from Macedonia (although slightly shorter; see Petkovski 1971). This Balkan species also has a similar general shape of the seminal receptacle and fifth leg, but can be distinguished easily from its two East Asian congeners by the absence of an inner seta on the first exopodal segment of all swimming legs.

*Diacyclops suoensis* can be distinguished from the Korean *Diacyclops parasuoensis* sp. n. by the size of the dorsal caudal seta, as well as by the proportions of many armature elements and the ornamentation of most appendages. Most of these differences are arrowed in Figs 14–17. The very few differences in the patterm of pores and sensilla, and the identical armature of the antenna, however, probably indicate that these two species are not distantly related, and may form a monophyletic group together with *Diacyclops pseudosuoensis* and *Diacyclops hisuta* sp. n. They are all only remotely related to *Diacyclops ishidai* sp. n., *Diacyclops brevifurcus*, *Diacyclops leeae* sp. n., *Diacyclops hanguk* sp. n., and *Diacyclops parahanguk* sp. n.

#### Diacyclops pseudosuoensis sp. n.

urn:lsid:zoobank.org:act:248EC622-AD80-48A8-8650-E774FF0593D5

http://species-id.net/wiki/Diacyclops_pseudosuoensis

Diacyclops languidoides suoensis Ito – Ito 1957: p. 15, Figs 35–44, 46–48. Synonymy. [partim.]
Diacyclops languidoides suoensis Ito – Ito 1957: p. 15, fig. 45. [non]
Diacyclops languidoides suoensis n. subsp. – Ito 1954: p. 399, Figs 114–148. [non]
Diacyclops suoensis Ito – Ueda et al. 1996: p. 309, fig. 4; Lee et al. 2007: p. 162, Figs 7–8; Chang 2009: p. 478, Figs 263–264. [non]

##### Type locality.

Japan, Kagoshima prefecture, Amami-Oshima island, Amami city, Naze High School, approximately 28°22'N, 129°29'E, well with a pump.

##### Type material.

Holotype female (illustrated by Ito (1957): Figs 46-48), allotype male from type locality, and 10 paratype females from type locality originally deposited at the Limnological Laboratory, Faculty of Fisheries, Prefectural University of Mie, Tsu city, Mie Prefecture, Japan (however, many administrative changes since original deposition made current location of types impossible to determin); all collected 12 August 1954, leg. Y. Morimoto. [not examined]

Additional paratype male deposited at the Limnological Laboratory, Faculty of Fisheries, Prefectural University of Mie, Japan; collected from Japan, Kagoshima prefecture, Amami-Oshima island, Amami city, Naze township, approximately 28°22'N, 129°29'E, well with a pump; 19 August 1954, leg. Y. Morimoto. [not examined]

##### Etymology.

The species name is composed of the Greek noun *pseudos* (= lie) prefixed to the existing specific name *suoensis*. The new name refers to the assumed close relationship between these two congeners.

##### Description.

Female and male as described in Ito (1957) from Amami-Oshima, and illustrated in his figures 46-48 as *Diacyclops languidoides suoensis* Ito, 1954.

##### Remarks.

This species is most similar to *Diacyclops suoensis* but can be distinguished by the longer innermost terminal caudal setae, which are slightly longer than the outermost terminal caudal ones, as well as by the longer apical endopodal spines on the fourth leg. Both species share very long dorsal caudal setae, which are about twice as long as the caudal rami and thus unique in the *languidoides*-group. Although the original description of *Diacyclops suoensis* by Ito (1954) was brief, after our redescription of it herein based on the Lake Biwa material (see above), we can confirm that it and *Diacyclops pseudosuoensis* sp. n. share many other morphological features. Among these are the armature formula of all swimming legs, similar proportions of the caudal rami, and similar proportions of the segments and armature of the fifth leg. Unfortunately, Ito (1957) did not describe or illustrate the antenna or mouth appendages, and he illustrated only the segments (without armature elements) of the antennula, so these features cannot be compared. Note that Ito (1957: fig. 45) provided a drawing of the female genital double-somite that is actually taken from his earlier publication (Ito 1954) and does not pertain to the Amami-Oshima population.

As mentioned above, *Diacyclops parasuoensis* and *Diacyclops suoensis* are very similar to *Diacyclops pseudosuoensis* and *Diacyclops hisuta* sp. n., but probably only remotely related to *Diacyclops ishidai* sp. n., *Diacyclops brevifurcus*, *Diacyclops leeae* sp. n., *Diacyclops hanguk* sp. n.,and *Diacyclops parahanguk* sp. n.

#### Diacyclops hisuta sp. n.

urn:lsid:zoobank.org:act:60399FF2-6118-4562-8203-95AD07B09EAE

http://species-id.net/wiki/Diacyclops_hisuta

Diacyclops suoensis Ito – Ueda et al. 1996: p. 309, fig. 4. Synonymy.
Diacyclops languidoides suoensis n. subsp. – Ito 1954: p. 399, Figs 114–148. [non]
Diacyclops languidoides suoensis Ito – Ito 1957: p. 15, Figs 35–48. [non]
Diacyclops suoensis Ito – Lee et al. 2007: p. 162, Figs 7–8; Chang 2009: p. 478, Figs 263–264. [non]

##### Type locality.

Japan, Yamaguchi prefecture, Mine city, Shuho township, Akiyoshi, Akiyoshido cave, approximately 34°13'N, 131°18'E, stream flowing through the cave.

##### Type material.

Holotype female (illustrated by Ueda et al. (1996) in their figure 4A–E) and allotype male (illustrated by Ueda et al. (1996) in their figure 4F–L), originally deposited at the Nakajima Marine Biological Station, Ehime University, Matsuyama city, Ehime Prefecture, Japan (however, specimens probably destroyed during earthquake in 2001; Prof. Hiroshi Ueda pers. comm.); both collected at the type locality, 13 April 1996, leg. T. Kuramoto. [not examined]

##### Etymology.

The species name is dedicated to Professors Hiroshi Ueda (formerly Marine Biological Station, Ehime University; currently Usa Marine Biological Station, Kochi University) and Susumu Ohtsuka (Takehara Station, Setouchi Field Science Center, Hiroshima University), and to Dr. Tadashi Kuramoto (Akiyoshi-dai Museum of Natural History), who discovered this population and described it under the name *Diacyclops suoensis* Ito, 1954 (see Ueda et al. 1996). The name is composed of the first syllables of their given names and should be treated as a Latin noun in apposition.

##### Description.

Female and male as described in Ueda et al. (1996) from Akiyoshido cave, and illustrated in their figure 4 as *Diacyclops suoensis* Ito, 1954.

##### Remarks.

Ueda et al. (1996) stated that they identified the *Diacyclops* population from Akiyoshido cave as *Diacyclops suoensis* mainly based on the shape of its seminal receptacle, but they noted four significant morphological differences between their specimens and the two populations described by Ito (1954, 1957). Their decision could have been motivated by the fact that Akiyoshido cave lies only 40 km east-southeast from the type locality of *Diacyclops suoensis*, and that *Diacyclops suoensis* was also found much further east by Ito (1957). In our view, the differences already noted by Ueda et al. (1996) justify the erection of a new species. Not only does *Diacyclops hisuta* sp. n. differ from *Diacyclops suoensis* in the length of the dorsal caudal setae, proportions of the caudal rami, proportions of the genital double-somite, and size of the innermost terminal caudal setae, but it has all these characters in common with the Korean *Diacyclops parasuoensis* sp. n. (see above), in addition to slender and very elongated lateral arms of the seminal receptacle. It is quite clear that *Diacyclops hisuta* and *Diacyclops parasuoensis* form a sibling species pair, and they can only be distinguished at this stage by the habitus shape (much more slender in *Diacyclops parasuoensis*). It is possible that they also differ in the number of inner setae on the second endopodal segment of the first leg, if we assume that Ueda et al. (1996) found the armature formula of the population from Akiyoshido cave to be the same as that reported by Ito (1957). Unfortunately, the description and illustrations provided by Ueda et al. (1996) do not show details of any mouth appendage or the armature of the first three pairs of swimming legs, so many characters cannot be compared.

#### Diacyclops leeae sp. n.

urn:lsid:zoobank.org:act:74F8CA94-D206-4934-9FA6-5546EE8F15E1

http://species-id.net/wiki/Diacyclops_leeae

Diacyclops suoensis Ito – Lee et al. 2007: p. 162, Figs 7–8; Chang 2009: p. 478, Figs 263–264. Synonymy.
Diacyclops languidoides suoensis n. subsp. – Ito 1954: p. 399, Figs 114–148. [non]
Diacyclops languidoides suoensis Ito – Ito 1957: p. 15, Figs 35–48. [non]
Diacyclops suoensis Ito – Ueda et al. 1996: p. 309, fig. 4. [non]

##### Type locality.

Korea, Chungcheongbukdo, Danyang city, Yeongchun township, Ha village, Ondal-gul cave, 37°03'43"N, 128°28'59"E, puddles in the cave.

##### Type material.

Holotype female (illustrated by Lee et al. (2007) in their figures 7 and 8) deposited at the Department of Biological Science, Daegu University, Korea; collected at the type locality, 13 August 2007, leg. J. Lee, Y.G. Choi and W.R. Kim. [not examined]

##### Etymology.

The species name is dedicated to Dr Jimin Lee (formerly Institute of Basic Science, Daegu University, now Korea Institute of Ocean Science and Technology), who, with co-authors, discovered this population and described it under the name *Diacyclops suoensis* Ito, 1954 (see Lee et al. 2007). The name is a noun in the genitive singular.

##### Description.

Female as described by Lee et al. (2007) from Ondal-gul cave, and illustrated in their figures 7 and 8 as *Diacyclops suoensis* Ito, 1954.

##### Remarks.

Lee et al. (2007) and Chang (2009) stated that they identified the Korean *Diacyclops* populations from Ondal-gul cave and the Youncheon-gul lava tube (the latter represented by an unillustrated female) as *Diacyclops suoensis* Ito, 1954 mainly based on the shape of the seminal receptacle and the elongated dorsal caudal setae. They noted, however, that the dorsal caudal setae are not as elongated as in Japanese populations and also that the caudal rami have somewhat different proportions. Their taxonomic decision possibly reflects the fact that various previous records of this species indicated a wide range in Japan, as well as some variability in the proportions and armature of the caudal rami (Ito 1954, 1957; Ueda et al. 1996).

After the redescription of *Diacyclops suoensis* from Japan in this paper (see above), it is quite clear that the Korean specimens found by Lee et al. (2007) are not conspecific with it. Thus, we describe them as a new species, *Diacyclops leeae* sp. n. The two species are, in fact, only distantly related, which can be judged from their numerous morphological differences in the proportions of the genital double-somite, proportions of the caudal rami, length of the dorsal caudal seta, length of the innermost terminal caudal seta, presence/absence of an exopodal seta on the antenna, number of setae on the second endopodal segment of antenna, etc. The armature of the antenna of *Diacyclops leeae* probably indicates that this species is not even part of the morphological group formed by the Korean *Diacyclops parasuoensis* sp. n. and the Japanese *Diacyclops suoensis, D. pseudosuoensis* sp. n., and *Diacyclops hisuta* sp. n. (see above).

*Diacyclops leeae* differs from *Diacyclops parasuoensis* in the following characters: proportions ot the genital double-somite, proportions of the caudal rami, length of the dorsal caudal seta, length of the innermost terminal caudal seta, presence/absence of exopodal seta on the antenna, number of setae on the second endopodal segment of antenna, length of the proximal seta on the third endopodal segment of the third swimming leg, length of the proximal seta on the third endopodal segment of the fourth swimming leg, relative length of the apical spines on the third endopodal segment of the fourth swimming leg, number of spinule rows on the intercoxal sclerite of the fourth leg, and proportions of the distal segment of the fifth leg. Unfortunately, Lee et al. (2007) and Chang (2009) did not illustrate mouth appendages, so details of these limbs cannot be compared.

*Diacyclops leeae* seems to be more closely related to *Diacyclops languidoides* than to *Diacyclops parasuoensis*. The former species has been recorded from numerous surface-water and subterranean habitats in Korea (see Chang 2009) and is widely distributed in the Palearctic (Dussart and Defaye 2006). *Diacyclops languidoides* and *Diacyclops leeae* share the same armature and ornamentation of the antenna, as well as a similar ornamentation of the fourth leg (especially its coxa and intercoxal sclerite). This may indicate that they shared a recent common ancestor, or even that the stygophilic *Diacyclops languidoides* gave rise (i.e. is directly ancestral) to the stygobiotic *Diacyclops leeae* during the major oscillations of its wide distributional range that probably took place during the Pleistocene glacial cycles. This would explain the presence of a population of the *languidoides*-group in a lava tube on the volcanic Jeju Island, although we cannot be sure if it truly belongs to *Diacyclops leeae* since Lee et al. (2007) did not provide any illustrations of this population. These inter-relationships need to be studied further, using molecular methods in addition to comparative morphology. The two species mainly differ in the proportions of the caudal rami (longer in *Diacyclops languidoides*) and the relative length of the third endopodal segment of the fourth leg (Chang 2009). However, the very wide distribution of *Diacyclops languidoides*, with numerous described subspecies (see Dussart and Defaye 2006) and several reports of its extreme morphological variability both from Asia (i.e. Ito 1954) and Europe (i.e Kiefer 1968), probably indicate that it is a complex of species, as is the case for many other widely distributed freshwater cyclopoids (Monchenko 2000; Blàha et al. 2010; Karanovic and Krajicek 2012a).

Chang (2009) reported records of *Diacyclops suoensis* from four different localities in Korea in addition to Odal-gul cave and the Yoncheon-gul lava tube, but without any additional comments on morphological variability, and he only republished the drawings of Lee et al. (2007). It is thus impossible for us to confirm whether any of these populations is in fact conspecific with *Diacyclops leeae* or perhaps with *Diacyclops parasuoensis*.

#### Diacyclops hanguk sp. n.

urn:lsid:zoobank.org:act:737B001D-E1D9-4965-9661-999F15B67F49

http://species-id.net/wiki/Diacyclops_hanguk

**Figure 18.**
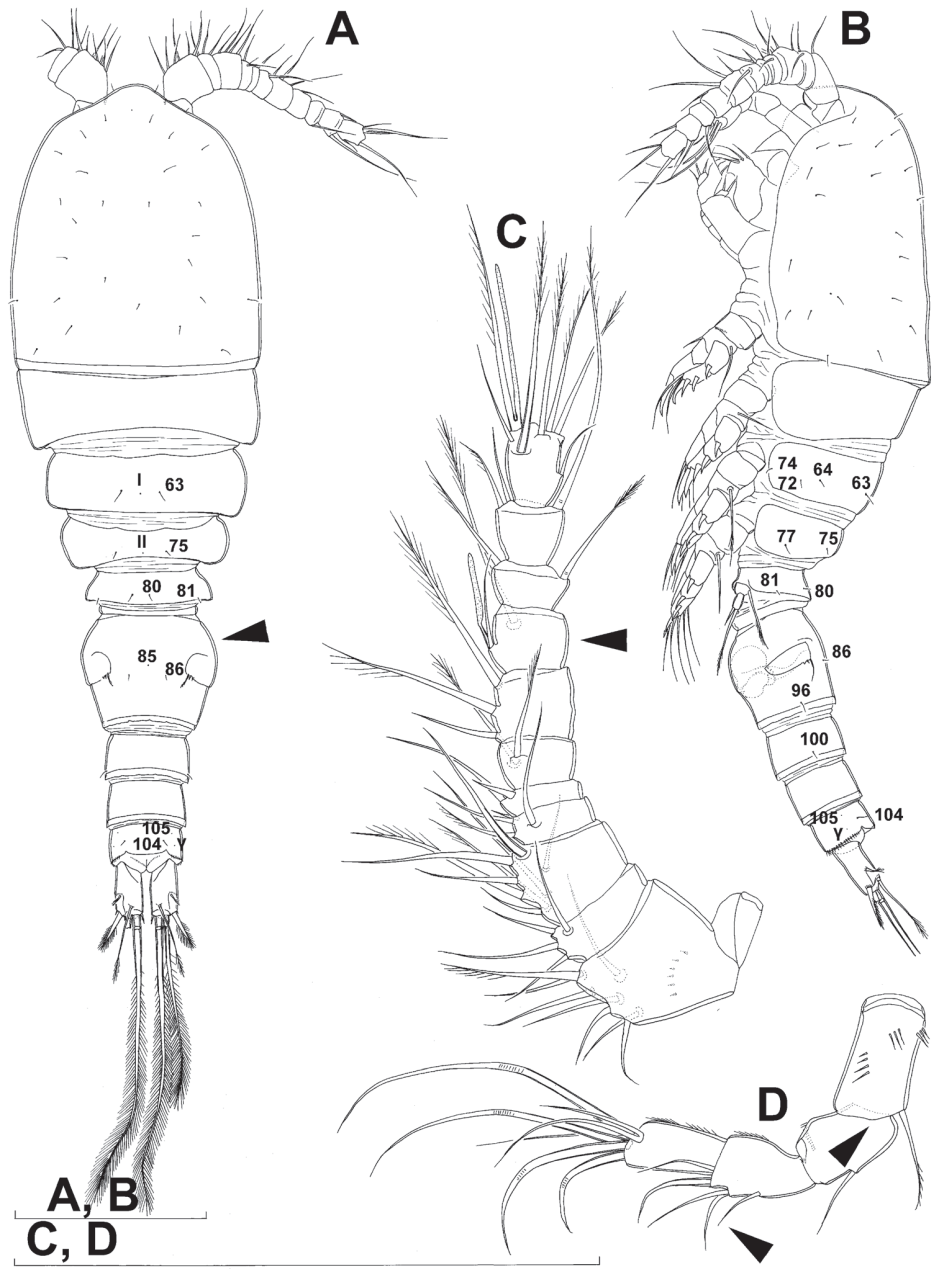
*Diacyclops hanguk* sp. n., holotype female: **A** habitus, dorsal view **B** habitus, lateral view **C** antennula, dorsal view **D** antenna, ventral view. Arabic numerals indicating sensilla and pores presumably homologous to those in *Diacyclops ishidai* sp. n. Roman numerals indicating pores homologous to those in *Diacyclops parasuoensis* sp. n. Arrows pointing most prominent specific features. Scale bars 100 μm.

Figs 18 [Fig F19] [Fig F20] [Fig F21] 22 27D

##### Type locality.

South Korea, Gangwondo, Pyeongchang city, Jinbu, Namhan River, 37°36'56.9"N, 128°32'23.2"E, interstitial water from sandy banks.

##### Type material.

Holotype female dissected on two slides (NIBRIV0000232661). Allotype male from type locality dissected on one slide (NIBRIV0000232662). Other paratypes from type locality: three males and three females on one SEM stub (NIBRIV0000232648), one female dissected on one slide (NIBRIV0000232663), and one male dissected on one slide (NIBRIV0000232664); all collected 12 June 2010, leg. J.-L. Cho.

Additional paratypes: one male and six females together in alcohol (NIBRIV0000232665), from South Korea, Gangwondo, Wonju city, Buron River, 37°14'01.23"N, 127°44'58.78"E, interstitial water from sandy banks, 24 June 2010, leg. J.-L. Cho.

Additional paratypes: five males, two females, and one copepodid together on one SEM stub (NIBRIV0000232653), from South Korea, Gangwondo, Yeongwol city, Namhan River, 37°06'56.9”N, 128°32'23.2”E, interstitial water from sandy banks, 12 June 2010, leg. J.-L. Cho.

Additional paratypes: one male and six females together in alcohol (NIBRIV0000232667), from South Korea, Jeollanamdo, Gurye city, Seomjin River, 35°11'25.4"N, 127°23'00.7"E, interstitial water from sandy banks, 19 June 2010, leg. J.-L. Cho.

Additional paratypes: one male, two females and one copepodid together in alcohol (NIBRIV0000232668), from South Korea, Gangwondo, Wonju city, Jijeong, Seom River, 37°23'10.16"N, 127°51'08.39"E, interstitial water from sandy banks, 24 June 2010, leg. J.-L. Cho.

Additional paratypes: two females and one copepodid together on one SEM stub (NIBRIV0000232653), from South Korea, Jeollanamdo, Gurye city, Yangcheon, Seomjin River, 35°12'04.7"N, 127°35'29.3"E, interstitial water from sandy banks, 19 June 2010, leg. J.-L. Cho.

##### Etymology.

The species name is a phonetic approximation in Latin letters of the country name “Korea” in the Korean language, to be treated as a Latin noun in apposition to the generic name.

##### Description.

**Figure 19. F19:**
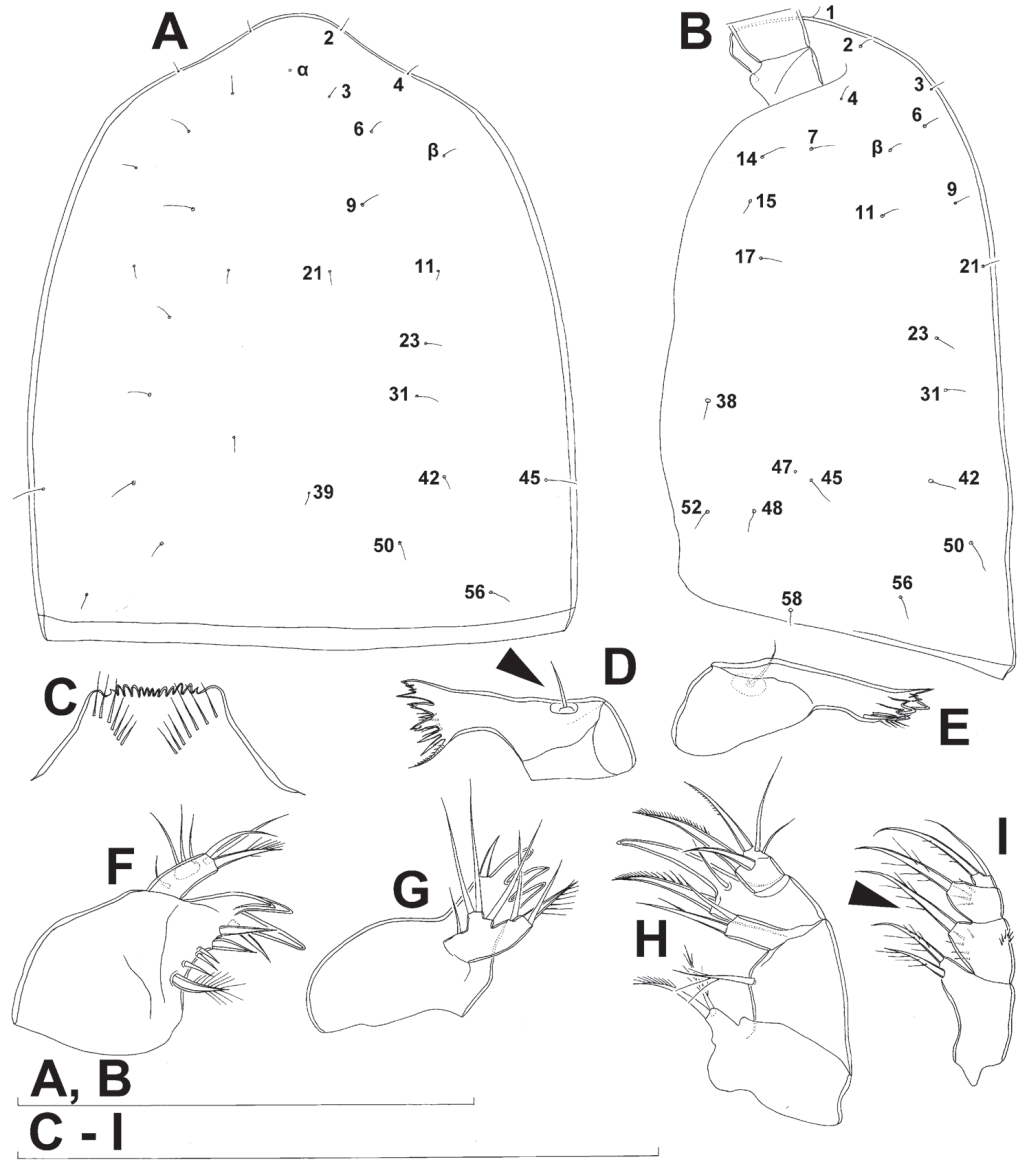
*Diacyclops hanguk* sp. n., holotype female: **A** cephalothorax, dorsal view **B** cephalothoracic shield, lateral view **C** labrum, anterior view **D** mandibula, posterior view **E** mandibula, antero-ventral view **F** maxillula, posterior view **G** maxillula, anterior view **H** maxilla, posterior view **I** maxilliped, posterior view. Arabic numerals indicating sensilla and pores presumably homologous to those in *Diacyclops ishidai* sp. n. Greek letters indicating unique pores and sensilla. Arrows pointing most prominent specific features. Scale bars 100 μm.

**Figure 20. F20:**
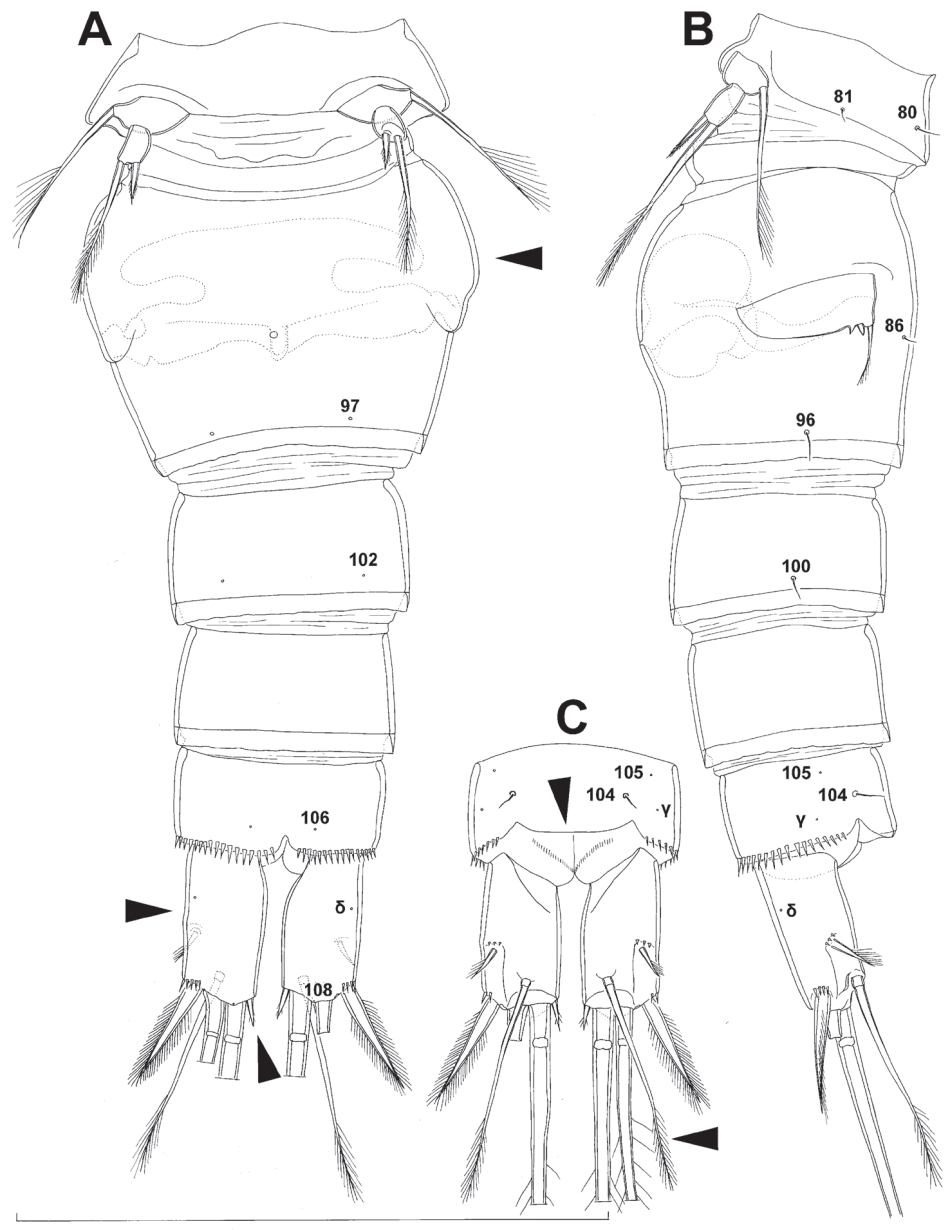
*Diacyclops hanguk* sp. n., holotype female: **A** urosome, ventral view **B** usorome, lateral view **C** anal somite and caudal rami, dorsal view. Arabic numerals indicating sensilla and pores presumably homologous to those in *Diacyclops ishidai* sp. n. Greek letters indicating unique pores and sensilla. Arrows pointing most prominent specific features. Scale bar 100 μm.

Female (based on holotype and four paratypes from type locality). Total body length, measured from tip of rostrum to posterior margin of caudal rami (excluding caudal setae), from 412 to 445 µm (440 µm in holotype). Preserved specimens colourless; no live specimens observed. Integument relatively weakly sclerotised, smooth, without cuticular pits or cuticular windows. Surface ornamentation of somites consisting of 44 pairs of sensilla and pores and four unpaired (mid-dorsal) pores (those pores and sensilla probably homologous with those of *Diacyclops ishidai* indicated with same Arabic numerals; those homologous with those of *Diacyclops parasuoensis* indicated with Roman numerals; presumably novel pores and sensilla indicated with Greek letters consecutively from anterior to posterior end of body, and from dorsal to ventral side in Figs 18A, B, 19A, B, 20A, B, C); no spinules except on anal somite, caudal rami, and appendages. Habitus (Fig. 18A, B) relatively slender, only slightly dorso-ventrally compressed, with prosome/urosome length ratio 1.4 and greatest width in dorsal view at first third of cephalothorax, body prominently arched backwards between prosome and urosome. Body length/width ratio about 3.3 (dorsal view); cephalothorax 1.84 times as wide as genital double-somite. Free pedigerous somites without lateral or dorsal expansions, all connected by well developed arthrodial membranes and having narrow and smooth hyaline fringes. Pleural areas of cephalothorax and free pedigerous somites very short, not covering insertions of cephalic appendages or praecoxae of swimming legs in lateral view.

Rostrum (Fig. 19A, B) well developed, membranous, not demarcated at base, broadly rounded and furnished with one pair of frontal sensilla (no. 1).

Cephalothorax (Figs 18A, B, 19A, B) relatively small, 1.2 times as long as its greatest width (dorsal view), widest at posterior third and gently tapering anteriorly and posteriorly, only slightly oval; representing 35% of total body length (together with rostrum). Surface of cephalic shield ornamented with one unpaired dorsal pore (α) and 25 pairs of long sensilla (nos. 2-4, 6, β, 7, 9, 11, 14, 15, 17, 21, 23, 31, 38, 39, 42, 45, 47, 48, 50, 52, 56, 58); sensilla pair no. 39 highly asymmetrical; sensilla and pores 39-58 belonging to first pedigerous somite, latter being incorporated into cephalothorax.

Second pedigerous somite (Figs 18A, B) well developed, only slightly narrower than cephalothorax and tapering posteriorly, unornamented.

Third pedigerous somite (Fig. 18A, B) shorter and narrower than second in dorsal view, widest at midlength in dorsal view and with slightly flared latero-posterior corners, ornamented with one unpaired dorsal pore (no. I) and four pairs of large sensilla (nos. 63, 64, 72, 74).

Fourth pedigerous somite (Fig. 18A, B) significantly shorter and narrower than third, with slightly flared latero-posterior corners, nicely rounded in dorsal view, ornamented with only one unpaired dorsal pore (no. II) and two pairs of large sensilla (nos. 75, 77); recognition of serially homologous pairs relatively easy (probably II=I, 75=63, 77=72).

Fifth pedigerous somite (Figs 18A, B, 20A, B) short, significantly narrower than fourth pedigerous somite or genital double-somite in dorsal view, with prominently flared latero-posterior corners, ornamented with two pairs of large dorsal sensilla (nos. 80, 81); recognition of serially homologous pairs relatively easy, i.e. 80=75 and 81=77; hyaline fringe very narrow and smooth.

Genital double-somite (Figs 18A, 20A, B) large but proportionately short (arrowed in Fig. 20A), with deep lateral recesses at level of sixth legs and only slightly swollen antero-ventrally, widest at first third of its length in dorsal (or ventral) view and gradually tapering posteriorly, 0.7 times as long as its greatest width (dorsal view); ornamented with one unpaired dorsal central pore (no. 85), one pair of central dorsal sensilla (no. 86), one pair of posterior lateral sensilla (no. 96), and one pair of ventral posterior pores (no. 97); central dorsal sensilla probably serially homologous to those on fifth pedigerous somite (i.e. 86=80), but posterior sensilla and pores without homologous pairs. Copulatory pore small, oval, situated at about 3/5 of genital double-somite ventrally; copulatory duct relatively wide, siphon-shaped and directed anteriorly, weakly sclerotised. Hyaline fringe wavy, not serrated. Seminal receptacle anvil-shaped, with relatively short anterior expansion and long lateral arms, constricted at middle, and with equally long and wide posterior expansion, together representing 52% of double-somite’s length; ovipores situated dorso-laterally at midlength of double-somite, covered by reduced sixth legs.

Third urosomite (Figs 18A, 20A, B) relatively short, about 1.9 times as wide as long and 0.4 times as long as genital double-somite in dorsal view, also with wavy hyaline fringe, ornamented with one pair of lateral posterior sensilla (no. 100) and one pair of ventral posterior pores (no. 102); serially homologous pores and sensilla easy to recognize, i.e. 100=96 and 102=97.

Preanal urosomite (Figs 18A, 20A, B) slightly narrower and shorter than third, also with wavy hyaline fringe, unornamented.

Anal somite (Figs 18A, 20A, B, C) slightly narrower and significantly shorter than preanal, with short medial cleft, ornamented with one pair of large dorsal sensilla (no. 104), two pairs of dorso-lateral pores (nos. 105, γ), one pair of small ventral pores (no. 106), and continous posterior row of large spinules. Anal sinus with two diagonal rows of short, slender spinules. Anal operculum very wide (arrowed in Fig. 20C), slightly convex, reaching posterior margin of anal somite, and representing 54% of anal somite’s width.

Caudal rami (Fig. 20A, B, C) very short (arrowed in Fig. 20A), almost cylindrical and parallel, inserted very close to each other (space between them less than half of width of ramus), with deep dorso-median anterior depression (as continuation of anal sinus), and with narrower base than rest of ramus (particularly in ventral view); rami approximately 1.8 times as long as wide (ventral view) and 1.6 times as long as anal somite, each armed with six setae (one dorsal, one lateral, and four terminal); ornamented with one ventral pore at 1/3 length, one pore on tip of small protuberance on distal ventral margin between two principal terminal setae (no. 108), and rows of small spinules at base of lateral setae. Dorsal seta slender and long (arrowed in Fig. 20C), about 1.4 times as long as ramus, inserted at 5/6 of ramus length, biarticulate at base (inserted on small pseudo-joint) and pinnate distally. Lateral seta small, inserted dorso-laterally at 2/3 of ramus length, about 0.6 times as long as ramus width, unipinnate laterally and uniarticulate at base. Outermost terminal seta stout, spiniform, 0.8 times as long as ramus, densely bipinnate. Innermost terminal seta minute (arrowed in Fig. 20A), sparsely pinnate, 0.2 times as long as outermost terminal seta. Two principal terminal setae with breaking planes, bipinnate; inner one about 1.5 times as long as outer one and 5.3 times as long as caudal rami.

Antennula (Fig. 18C) 11-segmented, with very short eighth segment (arrowed in Fig. 18C), slightly curved along caudal margin, directed laterally, not reaching posterior margin of cephalothoracic shield, ornamented only with proximo-ventral arc of spinules on first segment (no pits or other integumental structures), with armature formula as in *Diacyclops ishidai*. Only one seta on tenth segment with breaking plane, no seta biarticulate basally, and most larger setae sparsely pinnate distally; both aesthetascs very slender, that on eighth segment reaching posterior margin of ninth segment. One seta on fourth and one on fifth segment spiniform and short; all other setae slender; one apical seta on eleventh segment fused basally to aesthetasc. Length ratio of antennular segments, from proximal end and along caudal margin, 1 : 0.4 : 0.6 : 0.3 : 0.2 : 0.5 : 0.9 : 0.7 : 0.5 : 0.7 : 1.

**Figure 21. F21:**
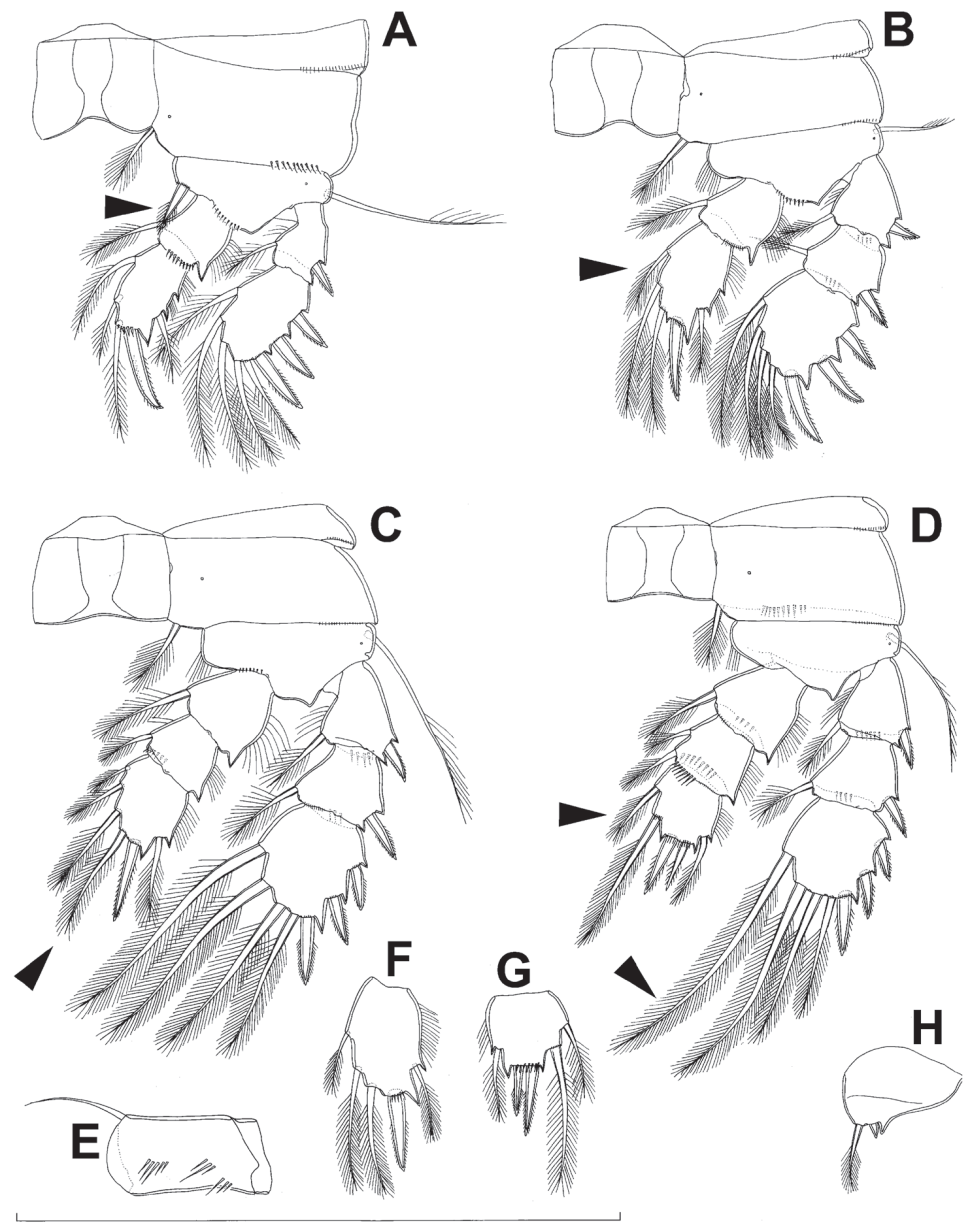
*Diacyclops hanguk* sp. n., **A–D** holotype female **E–H** paratype female **A** first swimming leg, anterior view **B** second swimming leg, anterior view **C** third swimming leg, anterior view **D** fourth swimming leg, anterior view **E** coxa and basis of antenna, ventral view **F** second endopodal segment of second swimming leg, anterior view **G** third endopodal segment of fourth swimming leg, anterior view **H** sixth leg, lateral view. Arrows pointing most prominent specific features. Scale bar 100 μm.

Antenna (Figs 18D, 21E) five-segmented, strongly curved along caudal margin, comprising extremely short coxa, much longer basis, and three-segmented endopod. Coxa without armature or ornamentation, about 0.2 times as long as wide. Basis cylindrical, 1.5 times as long as wide, ornamented with three short rows of three or four spinules each on ventral surface, armed with only one seta on distal inner corner (exopodal seta absent). First endopodal segment slightly narrowed at base and with small expansions on caudal margin but generally cylindrical, 1.4 times as long as wide and 0.9 times as long as basis, with smooth inner seta at 2/3 length and row of minute spinules caudo-dorsally. Second endopodal segment also with narrowed basal part, 1.6 times as long as wide, about as long as first endopodal segment, bearing only five smooth setae along inner margin (arrowed in Fig. 18D), ornamented with one row of spinules along caudal margin. Third endopodal segment cylindrical, 1.9 times as long as wide and slightly shorter than second endopodal segment, ornamented with two rows of slender spinules along caudal margin and armed with seven smooth apical setae (four of them strong and geniculate).

Labrum (Fig. 19C) a relatively large trapezoidal plate, with muscular base and strongly sclerotised distal margin (cutting edge), ornamented with two diagonal rows of seven long and slender spinules each on anterior surface. Cutting edge almost straight, with 13 more or less sharp teeth between produced and rounded lateral corners.

Mandibula (Fig. 19D, E) composed of coxa and minute palp. Cutting edge of coxal gnathobase with four slender spinules on anterior surface, five apical teeth, and dorsalmost unipinnate seta. Ventralmost tooth strongest and quadricuspidate, second and third teeth from ventral side bicuspidate, two dorsalmost teeth unicuspidate and partly fused basally. Palp represented by extremely small but distinct segment, unornamented, armed with single short and smooth apical seta (arrowed in Fig. 19D).

Maxillula (Fig. 19F, G) composed of praecoxa and one-segmented large palp, unornamented. Praecoxal arthrite bearing four very strong and smooth distal spines (three blunt and fused at base, one distinct at base and sharp) and six medial elements (proximalmost one longest and plumose, two distalmost ones large and strong, three in between small and slender). Palp composed of coxobasis and one-segmented endopod, with latter fused basally to coxobasis. Coxobasis with short proximal seta (probably representing exopod) and three medial setae (two slender and smooth, one strong and pinnate). Endopod with three slender, smooth setae.

Maxilla (Fig. 19H) 5-segmented but praecoxa partly fused to coxa on anterior surface, unornamented. Proximal endite of praecoxa robust, armed with two sparsely bipinnate setae; distal endite slightly smaller than proximal one and unarmed. Proximal endite of coxa with one bipinnate seta; distal endite highly mobile, elongated and armed apically with two setae, proximal one considerably longer and stronger than distal one. Basis expanded into robust and smooth claw, armed with two setae; strong seta densely pinnate along convex (ventral) margin, robust and spiniform; small seta smooth and slender, inserted on posterior surface. Endopod two-segmented but segmentation not easily discernable; proximal segment armed with two robust, unipinnate setae; distal segment with one robust, unipinnate apical seta and two slender subapical setae. Longest seta on distal endopodal segment 0.8 times as long as longer seta on proximal segment. All strong setae on basis and endopod, as well as basal claw, unguiculate.

Maxilliped (Fig. 19I) four-segmented, composed of syncoxa, basis, and two-segmented endopod; second endopodal segment minute; basis with only one armature element (arrowed in Fig. 19I). Ornamentation consisting of two rows of long, slender spinules on basis (one row on posterior surface, other on anterior surface), as well as four spinules on anterior surface of first endopodal segment. Armature formula: 2.1.1.2. All inner setae pinnate, very strong, and unguiculate.

All swimming legs (Fig. 21A, B, C, D, F, G) relatively small, with segmentation formula as in *Diacyclops parasuoensis*, as well as spine and setal formulae of ultimate exopodal segment, but with some differences in armature of endopods, ornamentation of different segments, and proportions of some segment and armature elements. Exopods slightly longer than endopods on all legs. All setae on endopods and exopods slender and plumose, except apical seta on exopod of first leg pinnate along outer margin and plumose along inner (Fig. 21A); no modified setae observed. All spines strong and bipinnate. Intercoxal sclerite of all swimming legs with slightly concave distal margin and lacking any surface ornamentation.

First swimming leg (Fig. 21A) shorter than other swimming legs; praecoxa unarmed, ornamented with distal row of minute spinules on anterior surface; coxa twice as wide as long, ornamented with distal row of large spinules on anterior surface and small pore on anterior surface close to inner margin, armed with slender plumose seta on inner-distal corner; basis almost pentagonal, 0.7 times as long as coxa, armed with long and slender seta outer seta and strong and short inner-distal element (latter not reaching distal margin of first endopodal segment, i.e. much shorter than in *Diacyclops ishidai* or *Diacyclops parasuoensis*;arrowed in Fig. 21A); inner margin smooth, ornamented with two posterior rows of minute spinules on anterior surface (one at base of inner seta, other at base of endopod), and one cuticular pore on anterior surface close to outer margin; exopod and endopod armed as in *Diacyclops parasuoensis*, but second segments more elongated, and second endopodal segment with inner notch and shorter middle inner seta.

Second swimming leg (Fig. 21B, F) similar to that of *Diacyclops parasuoensis*, but second endopodal segment with only three inner setae (arrowed in Fig. 21B), with or without inner notch (showing original segmentation), and third exopodal segment with all setae proportionatelly much shorter than in *Diacyclops parasuoensis*; apical spine on second endopodal segment 0.8 times as long as segment and 0.7 times as long as inner distal seta; second endopodal segment about 1.4 times as long as wide and 1.3 times as long as first endopodal segment.

Third swimming leg (Fig. 21C) similar to that of *Diacyclops parasuoensis*, but coxa without ornamentation on posterior surface, endopodal setae proportionately shorter (arrowed in Fig. 21C), and setae on third exopodal segment more obviously progressively longer from distal to proximal; apical spine on third endopodal segment 1.1 times as long as segment and 0.7 times as long as apical seta; third endopodal segment about as long as wide and 1.3 times as long as second endopodal segment.

Fourth swimming leg (Fig. 21D, G) generally similar to that of *Diacyclops parasuoensis*, but intercoxal sclerite unornamented, coxa without proximal row of spinules on posterior surface, proximal seta on third endopodal segment much shorter (arrowed in Fig. 21D), and proximal seta on third exopodal segment much longer (arrowed in Fig. 21D); third endopodal segment only about 0.9 times as long as wide, and only as long as second endopodal segment; inner apical spine on third endopodal segment 1.4 times as long as outer apical spine, about as long as segment, and less than half as long as distal inner seta; apical spines parallel.

Fifth leg (Fig. 20A, B) inserted ventrally, relatively small, two-segmented, with same armature as in previous four species, but with very different shape and proportions. Protopod relatively wide (although not as wide as in *Diacyclops ishidai*), almost rhomboidal, about 0.65 times as long as greatest width, unornamented, armed with single distally unipinnate and slender outer seta inserted on short setophore. Exopod much narrower than protopod, almost cylindrical, 1.1 times as long as protopod and twice as long as wide, unornamented, armed with long apical seta and subapical inner spine; apical seta bipinnate distally, as long as basal seta, 2.8 times as long as exopod, and 2.7 times as long as subapical spine, reaching midlength of genital double-somite; subapical exopodal spine strong, bipinnate, 0.9 times as long as exopod and almost twice as long as exopod’s greatest width.

Sixth leg (Fig. 21H) small semicircular cuticular plate, unornamented, armed with two short, smooth spines and, external to these, one longer and distally bipinnate seta; inner spine fused to plate, outer one articulated basally; outermost seta directed postero-dorsally.

**Figure 22.**
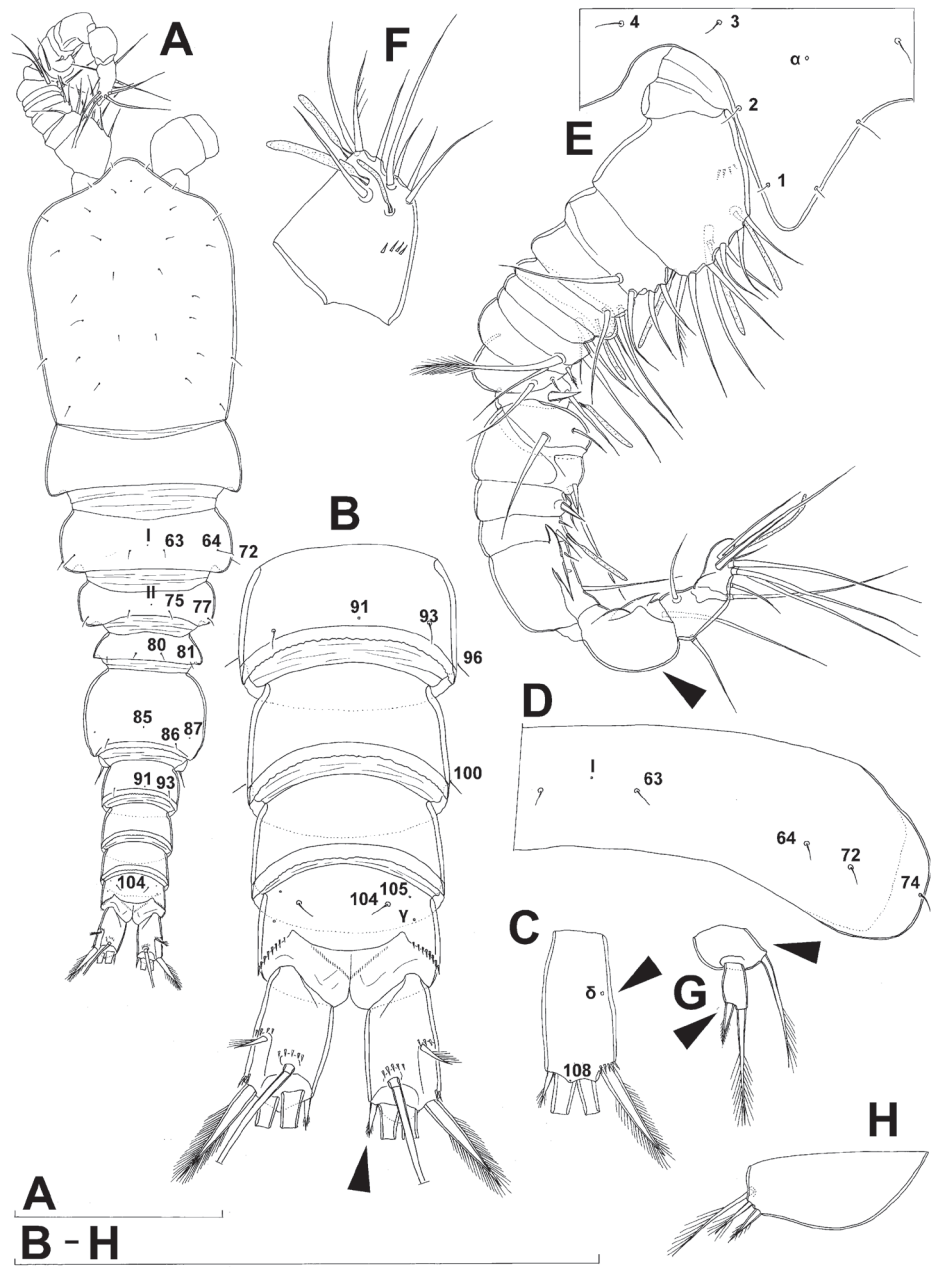
*Diacyclops hanguk* sp. n., allotype male: **A** habitus, dorsal view **B** last four urosomites and caudal rami, dorsal view **C** left caudal ramus, ventral view **D** pleuron of second free prosomite (third pedigerous somite), flattened **E** rostrum and antennula, flattened, dorsal view **F** first antennular segment, ventral view **G** fifth leg, anterior view **H** sixth leg, ventro-lateral view. Arabic numerals indicating sensilla and pores presumably homologous to those in *Diacyclops ishidai* sp. n. Roman numerals indicating pores homologous to those in *Diacyclops parasuoensis* sp. n. Greek letters indicating unique pores and sensilla. Arrows pointing most prominent specific features. Scale bars 100 μm.

Male (based on allotype and five paratypes from type locality). Total body length from 380 to 405 µm (383 µm in allotype). Urosome with free genital somite. Habitus (Fig. 22A) more slender than in female, with prosome/urosome length ratio about 1.5 and greatest width in dorsal view at first third of cephalothorax. Body length/width ratio 3.9; cephalothorax about 1.7 times as wide as genital somite. Cephalothorax 1.3 times as long as wide and slightly tapering towards posterior margin in dorsal view; representing 34% of total body length; dorsal sensilla pair no. 39 symmetrical. Ornamentation of cephalothorax (Fig. 22A), free prosomites (Fig. 22D), and first and last three urosomites (Fig. 22A, B) with same number and distribution of sensilla and pores as in female.

Genital somite (Fig. 22A) 1.5 times as wide as long in dorsal view, with bluntly serrated hyaline fringe dorsally, ornamented with one unpaired dorsal pore (no. 85), one pair of dorsal sensilla (nos. 86), and one pair of dorsolateral pores (no. 87; N.B., these absent in female); no spermatophores visible inside. Third urosomite (Fig. 22B) homologous to posterior part of female genital double-somite, ornamented with ventral pair of posterior pores (no. 97) and lateral pair of sensilla (no. 96) as in female, but additionally with dorsal unpaired pore (no. 91) and dorsal pair of sensilla (no. 93).

Caudal rami (Fig. 22B, C) slightly more divergent than in female, but equally short, with minute innermost terminal seta (arrowed in Fig. 22B) and with anterior ventral pore (δ; arrowed in Fig. 22C).

Antennula (Fig. 22E, F) strongly prehensile and digeniculate, 17-segmented (but with sixteenth and seventeenth segments partly fused), ornamented with spinules only on first segment (as in female), with anvil-shaped cuticular ridges on anterior margin of fourteenth and fifteenth segments (distal geniculation), with extremely short sixteenth segment (arrowed in Fig. 22E). Armature formula as follows: 8+3ae.4.2.2+ae.2.2.2.2.2+ae.2.2.2.2 + ae.2.1.4.7+ae. All aesthetascs linguiform, slender and short, apical one on sixteenth segment fused basally to one seta.

Antenna, labrum, mandibula, maxillula, maxilla, swimming legs, and fifth leg as in female.

Sixth leg (Fig. 22H) large cuticular plate, unornamented, armed with small inner spine and two bipinnate setae on outer distal corner; outermost seta 2.2 times as long as inner bipinnate seta, as well as 2.4 times as long as innermost spine.

##### Remarks.

**Figure 23.**
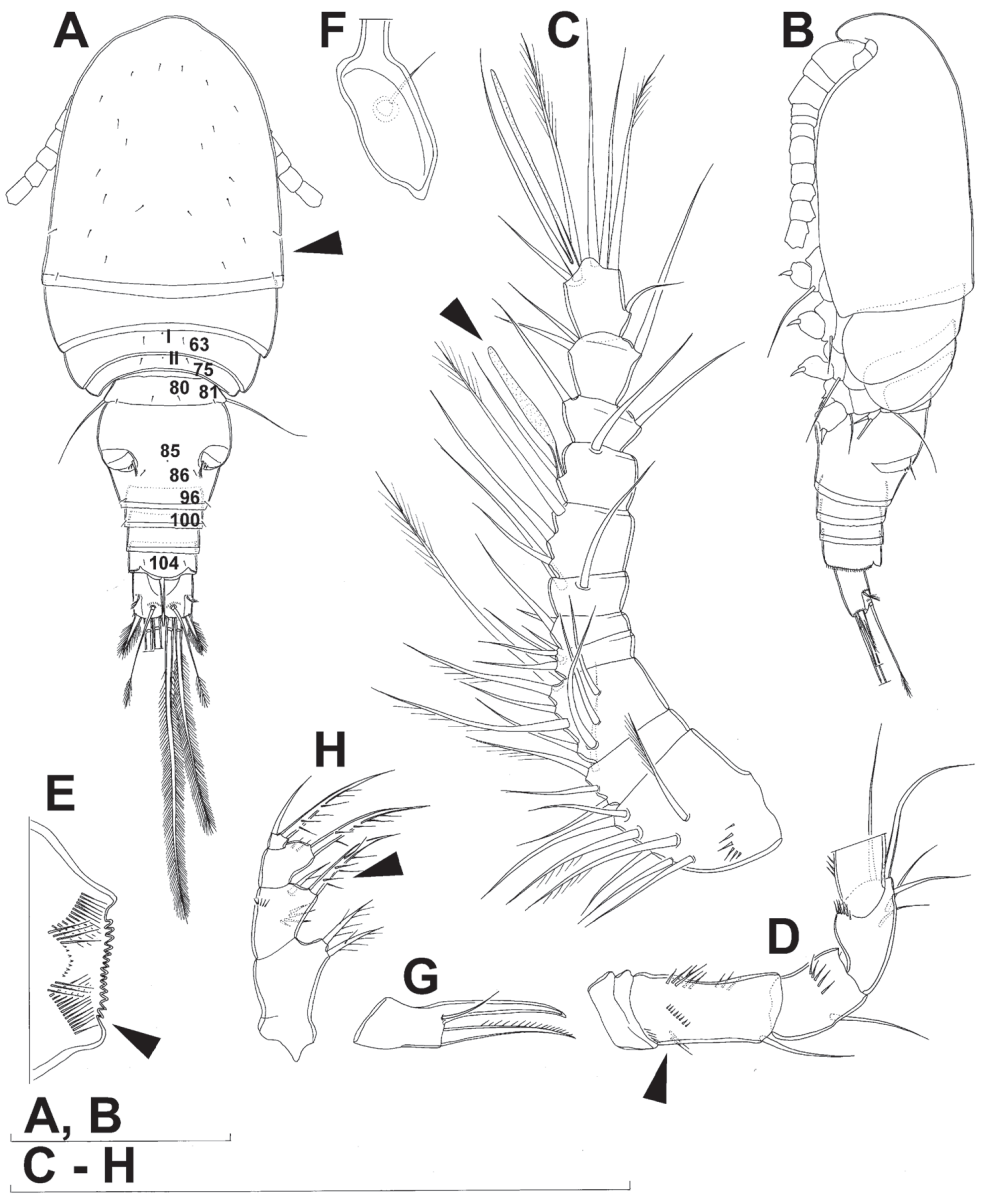
*Diacyclops parahanguk* sp. n., holotype female: **A** habitus, dorsal view **B** habitus, lateral view **C** antennula, ventral view **D** antenna, ventral view **E** labrum, anterior view **F** mandibula without cutting edge, dorsal view **G** basis of maxilla, posterior view **H** maxilliped, posterior vew. Arabic numerals indicating sensilla and pores presumably homologous to those in *Diacyclops ishidai* sp. n. Roman numerals indicating pores homologous to those in *Diacyclops parasuoensis* sp. n. Arrows pointing most prominent specific features. Scale bars 100 μm.

**Figure 24. F24:**
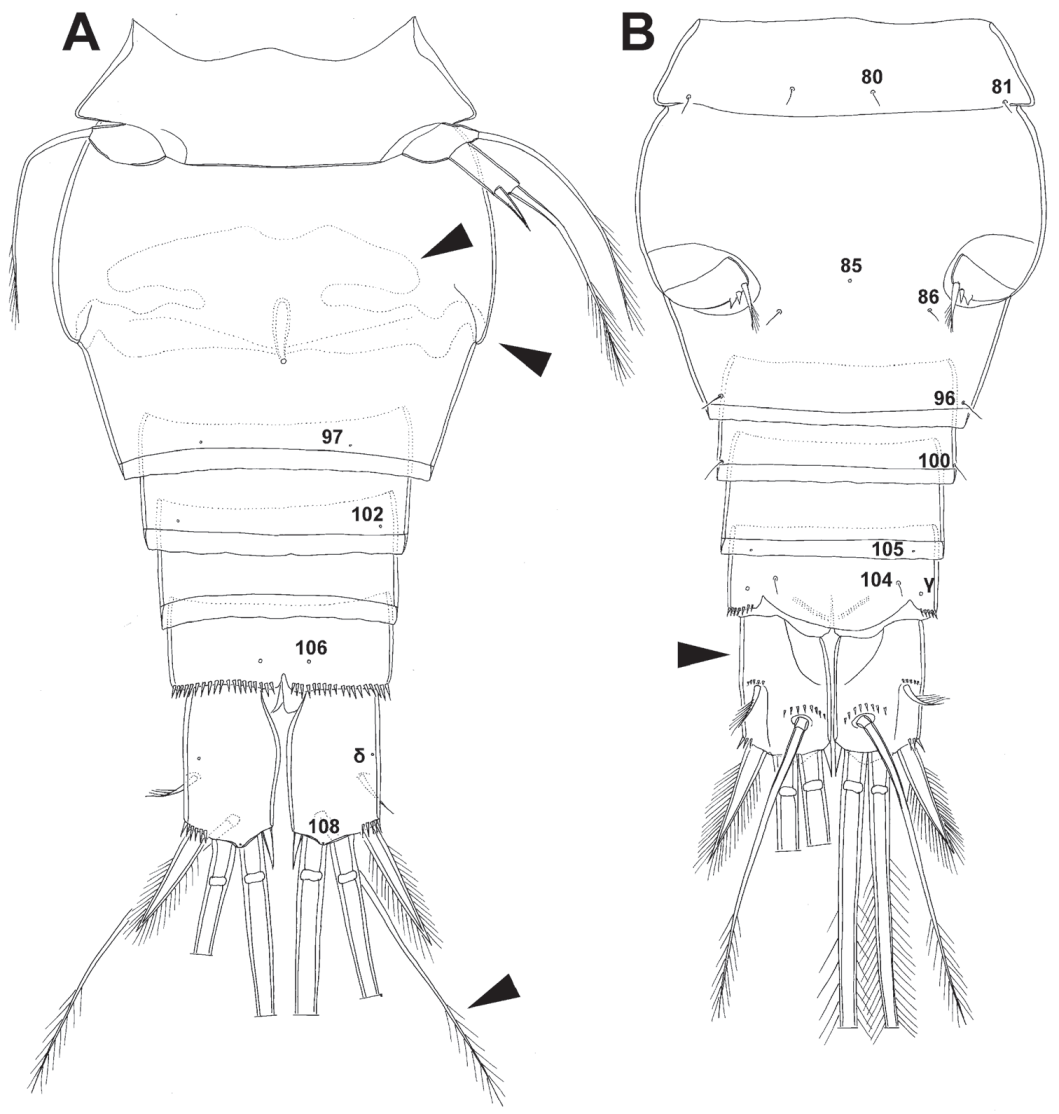
*Diacyclops parahanguk* sp. n., holotype female: **A** urosome, ventral view **B** urosome, dorsal view. Arabic numerals indicating sensilla and pores presumably homologous to those in *Diacyclops ishidai* sp. n. Greek letters indicating pores and sensilla homologous to those in *Diacyclops hanguk* sp. n. Arrows pointing most prominent specific features. Scale bar 100 μm.

**Figure 25.**
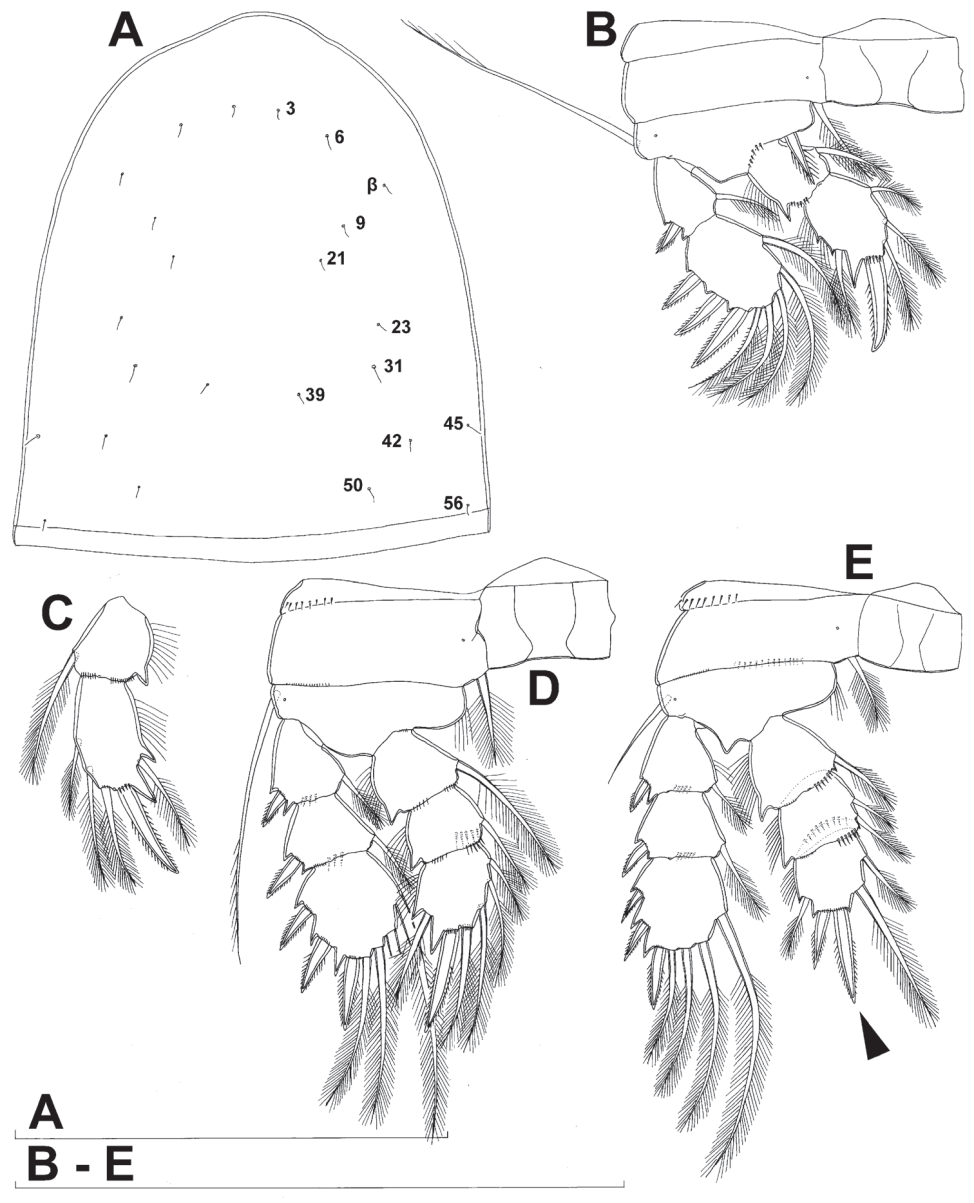
*Diacyclops parahanguk* sp. n., holotype female: **A** cephalothorax, dorsal view **B** first swimming leg, anterior view **C** endopod of second swimming leg, anterior view **D** third swimming leg, anterior view **E** fourth swimming leg, anterior view. Arabic numerals indicating sensilla and pores presumably homologous to those in *Diacyclops ishidai* sp. n. Greek letters indicating pores and sensilla homologous to those in *Diacyclops hanguk* sp. n. Arrows pointing most prominent specific features. Scale bars 100 μm.

*Diacyclops hanguk* sp. n. belongs to the *languidoides*-group based on its 11-segmented antennula and the swimming leg segmentation formula (exopod/endopod) 2/2, 3/2, 3/3, 3/3. Detailed examination of the pattern of pores and sensilla on the somites and the armature of the mouth appendages show that this species is only distantly related to all other East Asian species of this species-group except for *Diacyclops parahanguk* sp. n. from Japan (see below). It it quite clear that the two form a sibling species pair, with their extremely short caudal rami and genital double somite, extremely wide anal operculum, almost identical pattern of pores and sensilla on all somites, minute innermost terminal caudal seta, long dorsal caudal seta, wider than long eighth segment of the antennula, extremely reduced armature formula of the antenna (1.1.5.7), single seta on the mandibular palp, identical armature formula of the swimming legs (with only three inner setae on the second endopodal segment of the first leg), extremely short third endopodal segment of the fourth leg, and progressively longer setae from the outer side on the third exopodal segment of the fourth leg. The two species can be distinguished by their habitus, armature of the maxillipedal basis, ornamentation of the labrum and antennal basis, relative length of the aesthetasc on the eighth antennular segment, small differences in the shape of the seminal receptacle, genital double-somite, and caudal rami, and relative lengths of the dorsal caudal seta and inner apical spine on the third endopodal segment of the fourth leg (all these arrowed in Figs 23–25). Additionally, small differences were observed in the pattern of pores and sensilla, with sensilla no. 21 less widely spaced in *Diacyclops hanguk*.

No other species of the *languidoides-*group, nor any other species of *Diacyclops*, has such short caudal rami and minute innermost terminal caudal setae. These characters are traditionally only found in much more reduced cyclopoids (usually with variously reduced fifth legs), such as members of the genus *Itocyclops* Reid & Ishida, 2000 from Japan and North America (see Reid and Ishida 2000). It would be easy to speculate how this monospecific genus could have originated from an acestor similar to *Diacyclops hanguk* through a series of reductions in both the swimming leg segmentation and the armature and segmentation of the fifth leg, if *Itocyclops* did not exibit a more plesiomorphic armature of the antenna and maxilliped.

The only other described *Diacyclops* with similarly short caudal rami is the Romanian *Diacyclops languidoides spelaeus* Pleşa, 1956. This species does not even belong to the *languidoides*-group, as is shown by the three-segmented exopod of its first leg (see Pleşa 1956), even though Dussart and Defaye (2006) recently still listed it as a subspecies of *Diacyclops languidoides* (Lilljeborg, 1901). The latter authors mentioned that *Diacyclops languidoides spelaeus* could be a junior synonym of *Diacyclops stygius deminutus* (Chappuis, 1925), following Monchenko (1974), but this is just a speculation until both taxa are properly redescribed. Both differ from *Diacyclops hanguk* by the segmentation of the swimming legs, and by their much longer innermost terminal caudal seta.

#### Diacyclops parahanguk sp. n.

urn:lsid:zoobank.org:act:17419867-DAB4-433C-A66D-203E554CFA2E

http://species-id.net/wiki/Diacyclops_parahanguk

Figs 23 [Fig F24] 25

##### Type locality.

Japan, Shiga prefecture, Otsu city, Lake Biwa, Matsunoura Beach, lake beach next to inflow of small but fast-flowing irrigation runoff canal, 35°12.319'N, 135°55.768'E, interstitial water from medium to coarse sand.

##### Type material.

Holotype female dissected on one slide (LBM1430005391), collected at type locality, 4 October 2009, leg. T. Karanovic.

##### Etymology.

The species name is composed of the existing specific name *hanguk* proposed herein above (Korean for “Korea”) and the Greek prefix *para* (= near, beside), and refers to the close relationship between these two congeners.

##### Description.

Female (based on holotype). Total body length, measured from tip of rostrum to posterior margin of caudal rami (excluding caudal setae), 274 µm. Preserved specimen colourless; no live specimen observed. Integument weakly sclerotised, smooth, without cuticular pits or cuticular windows. Surface ornamentation of somites consisting of 44 pairs of sensilla and pores and four unpaired (mid-dorsal) pores as in *Diacyclops hanguk*; no spinules except on anal somite, caudal rami, and appendages. Habitus (Fig. 23A, B) relatively robust (arrowed in Fig. 23A), strongly dorso-ventrally compressed, with all somites strongly telescopically contracted; prosome/urosome length ratio 1.5 and greatest width in dorsal view at posterior end of cephalothorax, not arched backwards between prosome and urosome. Body length/width ratio about 2.5 (dorsal view); cephalothorax 1.8 times as wide as genital double-somite. Free pedigerous somites without lateral or dorsal expansions, but with narrow and smooth hyaline fringes. Pleural areas of cephalothorax and free pedigerous somites better developed than in *Diacyclops hanguk*, covering insertions of cephalic appendages and praecoxae and partly coxae of swimming legs in lateral view.

Rostrum (Fig. 23B) well developed, membranous, not demarcated at base, broadly rounded and ornamented, as in *Diacyclops hanguk*, with one pair of frontal sensilla (no. 1).

Cephalothorax (Figs 23A, B, 25A) large, 1.15 times as long as its greatest width (dorsal view), widest at posterior end and oval; representing 47% of total body length (together with rostrum). Surface of cephalic shield ornamented as in *Diacyclops hanguk*, with one unpaired dorsal pore and 25 pairs of long sensilla; sensilla pair no. 39 not asymmetrical, and pair no. 21 more widely spaced than in *Diacyclops hanguk*; sensilla and pores 39-58 belonging to first pedigerous somite, incorporated into cephalothorax.

Second pedigerous somite (Figs 23A, B) well developed, only slightly narrower than cephalothorax and tapering posteriorly, unornamented.

Third pedigerous somite (Fig. 23A, B) shorter and narrower than second in dorsal view, widest at anterior end and tapering posteriorly, ornamented as in *Diacyclops hanguk*, with one unpaired dorsal pore (no. I) and four pairs of large sensilla (nos. 63, 64, 72, 74).

Fourth pedigerous somite (Fig. 23A, B) significantly shorter and narrower than third, also tapering posteriorly, ornamented as in *Diacyclops hanguk* with one unpaired dorsal pore (no. II) and two pairs of large sensilla (nos. 75, 77).

Fifth pedigerous somite (Fig. 24A, B) significantly narrower than fourth pedigerous somite or genital double-somite in dorsal view, with flared latero-posterior corners, ornamented with two pairs of large dorsal sensilla (nos. 80, 81).

Genital double-somite (Figs 24A, B) even larger and wider (arrowed in Fig. 24A) than in *Diacyclops hanguk*, 0.75 times as long as its greatest width (dorsal view), but with same ornamentation an similarly shaped seminal receptacle, anterior expansion of seminal receptacle with median saddle and shorter lateral arms (arrowed in Fig. 24A) than in *Diacyclops hanguk*, hyaline fringe wavy.

Third and fourth urosomites (Fig. 24A, B) as in *Diacyclops hanguk*.

Anal somite (Fig. 24A, B) also very similar to that of *Diacyclops hanguk*, but with wider and more posteriorly produced, convex anal operculum, this reaching posterior margin of anal somite and representing 70% of anal somite’s width.

Caudal rami (Fig. 24A, B) very short (arrowed in Fig. 24B), approximately 1.6 times as long as wide (ventral view) and 1.7 times as long as anal somite, almost cylindrical and parallel, inserted very close to each other (with hardly any space between), with deep dorso-median anterior depression (as continuation of anal sinus), with narrower base than rest of ramus in ventral view; armed and ornamented as in *Diacyclops hanguk* except for presence of arc of spinules at base of dorsal seta and proportionately longer dorsal seta (arrowed in Fig. 24A).

Antennula (Fig. 23C) with aesthetasc on eighth segment broad and reaching posterior margin of tenth segment (arrowed in Fig. 23C); other armature, as well as segmentation, ornamentation, and size proportions of segments as in *Diacyclops hanguk*.

Antenna (Fig. 23D) with segmentation and armature as in *Diacyclops hanguk*; basis twice as long as wide and ornamanted with two additional rows of spinules (arrowed in Fig. 23D); setae on second endopodal segment proportionatelly somewhat longer than in *Diacyclops hanguk*.

Labrum (Fig. 23E) ornamented with two diagonal rows of 16 long and slender spinules each on anterior surface (arrowed in Fig. 23E), and with short central row of minute spinules in between; cutting edge almost straight, with 14 teeth between produced and rounded lateral corners.

Mandibula (Fig. 23F), maxillula, and maxilla (Fig. 23G) as in *Diacyclops hanguk*.

Maxilliped (Fig. 23H) with two setae on basis (arrowed in Fig. 23H) and with fewer spinules on first endopodal segment; armature formula: 2.2.1.2.

All swimming legs (Fig. 25B, C, D, E) with same segmentation, armature, and ornamentation as in *Diacyclops hanguk*, as well as proportions of most segments and armature elements; third endopodal segment of fourth leg about 0.9 times as long as wide, and 1.1 times as long as second endopodal segment; inner apical spine on third endopodal segment 1.6 times as long as outer apical spine (arrowed in Fig. 25E), about 1.3 times as long as segment, and 0.6 times as long as distal inner seta; apical spines diverging at approximately 20° angle.

Fifth leg (Fig. 24A) with slightly smaller protopod and longer exopod than in *Diacyclops hanguk*; protopod rhomboidal in shape, about 0.65 times as long as greatest width and unornamented, but basal seta inserted on longer setophore; exopod 1.8 times as long as protopod and 2.5 times as long as wide; apical exopodal seta bipinnate distally, as long as basal seta, 3.2 times as long as exopod, and 4.2 times as long as subapical spine, reaching 2/3 length of genital double-somite; subapical exopodal spine strong, bipinnate, 0.8 times as long as exopod and twice as long as exopod’s greatest width.

Sixth leg (Fig. 24B) as in *Diacyclops hanguk*.

Male. Not collected.

##### Remarks.

As mentioned above, *Diacyclops parahanguk* sp. n. forms a sibling species pair with the Korean *Diacyclops hanguk* sp. n.The two species can be distinguished by their habitus (wider in *Diacyclops parahanguk*), armature of the maxillipedal basis (one additional seta in *Diacyclops parahanguk*), ornamentation of the labrum (more spinules in *Diacyclops parahanguk*) and the basis of the antenna (more spinules in *Diacyclops parahanguk*), relative length of the aesthetasc on the eighth antennular segment (longer in *Diacyclops parahanguk*), and small differences in the shape of the seminal receptacle (shorter lateral arms in *Diacyclops parahanguk*), genital double-somite (wider in *Diacyclops parahanguk*), and caudal rami (shorter in *Diacyclops parahanguk*), and the relative lengths of the dorsal caudal seta (longer in *Diacyclops parahanguk*) and inner apical spine on the third endopodal segment of the fourth leg (longer in *Diacyclops parahanguk*). All these differences are marked with arrows in Figs 23–25. Also, sensilla pair no. 21 on the cephalothorax is more widely spaced in *Diacyclops parahanguk*.

**Figure 26.**
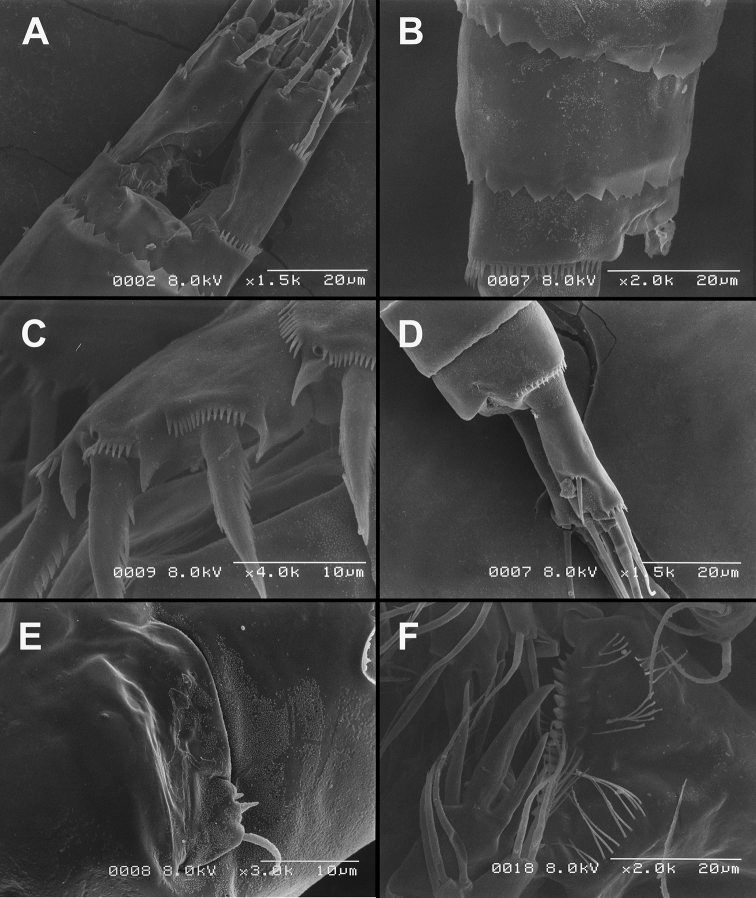
Scanning electron micrographs, **A–C** *Diacyclops ishidai* sp. n. **D–E** *Diacyclops parasuoensis* sp. n. **F** *Diacyclops suoensis* Ito, 1954: **A** anal somite and caudal rami, dorsal view, paratype female 1 **B** preanal and anal somites, lateral view, paratype female 2 **C** last two exopodal segments of second swimming legs, lateral view, paratype female 2 **D** anal somite and caudal rami, lateral view, paratype female **E** sixth leg, lateral view, paratype female **F** labrum and maxillulae, ventral view. Scale bars 20 μm (**A, B, D, F**) and 10 μm (**C, E**).

**Figure 27.**
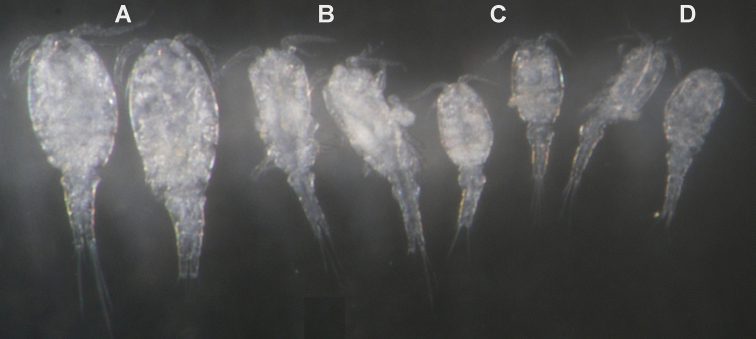
Light photograph of four sympatric Korean species of the *Diacyclops/Acanthocyclops* complex from Seomjin River: **A** *Acanthocyclops sensitivus* (Graeter & Chappuis, 1914), two adult females **B** *Diacyclops languidoides s.l*. (Lilljeborg, 1901), two adult females **C** *Diacyclops parasuoensis* sp. n., two adult females **D** *Diacyclops hanguk* sp. n., two adult females.

**Figure 28.**
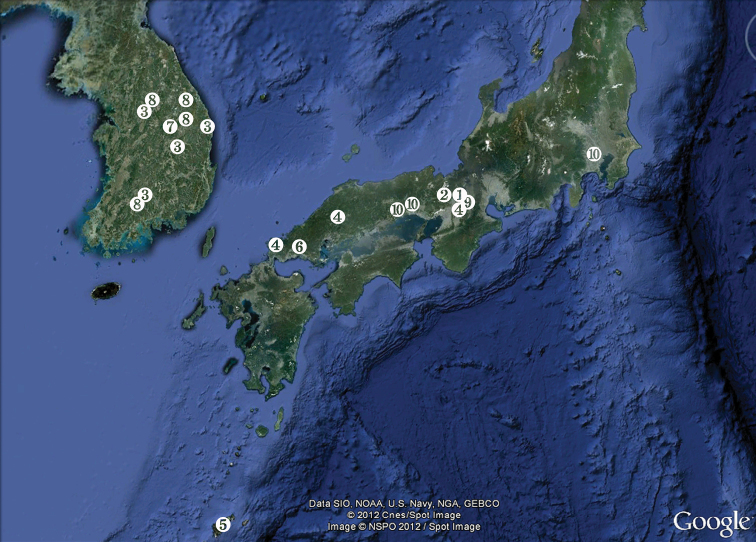
Distribution of East Asian endemic species from the *languidoides*-group of the genus *Diacyclops* Kiefer, 1927: **1** *Diacyclops ishidai* sp. n. **2** *Diacyclops brevifurcus* Ishida, 2006 **3** *Diacyclops parasuoensis* sp. n. **4** *Diacyclops suoensis* Ito, 1954 **5** *Diacyclops pseudosuoensis* sp. n. **6** *Diacyclops hisuta* sp. n. **7** *Diacyclops leeae* sp. n. **8** *Diacyclops hanguk* sp. n. **9** *Diacyclops parahanguk* sp. n. **10** *Diacyclops japonicus* Ito, 1952. Note that some numbers represent more than one locality. Map from Google Earth.

## Discussion

Morphological study of microcharacters, many of which are used here in cyclopoid taxonomy for the first time, has revealed a high diversity of the *languidoides*-group of the genus *Diacyclops* both in Korea and Japan. No subterranean species was found in common, although some may belong to the same or similar lineages, indicating relatively recent speciation. We have described seven new species of stygobionts, and discovered four sibling species pairs (two in Japan, and two in Korea and Japan). Three new species were described from Korea (*Diacyclops hanguk* sp. n., *Diacyclops leeae* sp. n., and *Diacyclops parasuoensis* sp. n.), and four from Japan (*Diacyclops hisuta* sp. n., *Diacyclops ishidai* sp. n., *Diacyclops parahanguk* sp. n., and *Diacyclops pseudosuoensis* sp. n.). *Diacyclops hanguk*, *Diacyclops parasuoensis*, *Diacyclops ishidai*, and *Diacyclops parahanguk* were described from newly collected material, while the other three new names were erected for populations of previously reported specimens identified by their discoverers as some of the more widely distributed species. *Diacyclops brevifurcus* Ishida, 2006 was redescribed from the holotype female, while *Diacyclops suoensis* Ito, 1954 was redescribed from newly collected material near the ancient Lake Biwa in Japan. Our initial hypothesis about high endemism of this group of subterranean animals was confirmed, and is in accordance with recently discovered endemism of subterranean harpacticoids in this region (Karanovic and Lee 2012; Karanovic et al. 2012b). This research provides evidence for both the importance of subterranean habitats as reservoirs of biodiversity, and current inadequate morphological methods of identifying closely related species of cyclopoid copepods. The disproportionately high diversity discovered around Lake Biwa provides further evidence in support of the hypothesis about the role of ancient lakes as biodiversity pumps for subterranean habitats, which was first proposed by Karanovic and Abe (2010).

All nine species of *Diacyclops* described or redescribed in this paper belong to the *languidoides-*group, based on the segmentation of their swimming legs and female antennula. Considering that they all come from a well-defined zoogeographical region, it would be reasonable to expect that they are also closely related; however, careful examination of the pattern of pores and sensilla on their somites, as well as the armature and ornamentation of their antennae and mouth appendages, all suggest that this is not a closely related group at all. There is no question that the Korean *Diacyclops hanguk* and the Japanese *Diacyclops parahanguk* are very closely related, as are the Korean *Diacyclops parasuoensis* and the Japanese *Diacyclops hisuta*, but the relationship between the *hanguk/parahanguk* pair and the *parasuoensis/hisuta* pair is quite remote. In fact, the *hanguk/parahanguk* pair has a somewhat isolated position in the *languidoides*-group, while the *parasuoensis/hisuta* pair is probably more closely related to several European species than to any of their East Asian congeners (see Remarks sections for the respective species above). The other two sibling species pairs are all Japanese endemics: *Diacyclops brevifurcus* from surface waters in Kyoto and Lake Biwa is very closely related to the subterranean *Diacyclops ishidai* from the vicinity of Lake Biwa, while *Diacyclops suoensis* from central and western Honshu is a sibling species of *Diacyclops pseudosuoensis* from the southern island of Amami-Oshima (both subterranean; see above). These four sibling species pairs probably represent four separate colonization events in the subterranean waters of East Asia from more widely distributed (probably Palaearctic) surface water ancestors, as they are not closely related to each other, and some of them have relatives in highly disjunct locations, without direct subterranean connections or plausible mechanisms of long distance dispersal. Actually, *Diacyclops brevifurcus* still lives in surface-water habitats, as Ishida (2006) collected one female from the floating bog bed at Mizoro-ga-ike pond and four females from surface water of Lake Biwa. The Korean *Diacyclops leeae* probably represents a fifth colonization event in East Asia, as it seems to be most closely related to the widely distributed *Diacyclops languidoides* (Lilljeborg, 1901), and the Japanese *Diacyclops japonicus* Ito, 1952 probably represents a sixth. A relatively remote relationship among these six species groups of East Asian *Diacyclops* is further supported by their ability to live sympatrically, and Korean members of different groups were often found in the same sample (Fig. 27). The fact that the size differentiation between them is not pronounced probably indicates that they are not competing for the same resources, which all points to different phylogenies and repeated colonizations (Karanovic and Cooper 2012). The existence of very few differences in the pattern of pores and sensilla and the similar armature of the antenna in *Diacyclops parasuoensis* and *Diacyclops suoensis* probably indicate that these two species are not very distantly related, and may form a monophyletic group together with *Diacyclops pseudosuoensis* and *Diacyclops hisuta* (and probably some European species). On the other hand, the pattern of pores and sensilla suggests that they are all only remotely related to *Diacyclops ishidai*, *Diacyclops brevifurcus*, *Diacyclops leeae*, *Diacyclops hanguk*, and *Diacyclops parahanguk*. Future studies of this species complex in East Asia will have to include molecular tools to test this hypothesis.

The highest concentration of subterranean species of *Diacyclops* in East Asia is found around the ancient Lake Biwa in Japan. Two of the more widely distributed Japanese species (*Diacyclops suoensis* and *Diacyclops japonicus*) have ranges that include this area and extend further either southwards (*Diacyclops suoensis*) or northwards (*Diacyclops japonicus*), while three species appear to be endemic to this small area of Honshu around Lake Biwa (Fig. 28). This is probably not a coincidence or a consequence of a sampling bias, as the subterranean waters in Korea were sampled quite systematically (see Fig. 28). We tend to attribute to ancient lakes a role of biodiversity pumps for subterranean habitats (Karanovic and Abe 2010), in addition to their role as refugia (Matzinger et al. 2006; Albrecht and Wilke 2008). Deep benthic environments in ancient lakes have very little light, and are therefore ideal for the evolution of subterranean adaptations (stygomorphies) that would enable copepods to easily colonize subterranean habitats subsequently. Korea has no ancient lakes, nor any significant natural freshwater lakes at all, and we speculate that this may be one of the main reasons for fewer subterranean species of the *languidoides*-group there (three) as compared with Japan (seven) (Fig. 28).

Also interesting is the fact that two species are relatively widely distributed in Korea (*Diacyclops hanguk* and *Diacyclops parasuoensis*), and two also in Japan (*Diacyclops suoensis* and *Diacyclops japonicus*), while six other species seem to be short-range endemics (sensu Harvey 2002; Eberhard et al. 2009). This also indicates that the six East Asian groups, distinguished by morphological microcharacters, may have had different colonization histories there and are of different ages. A similar case has been observed recently in two genera of the harpacticoid family Parastenocarididae Chappuis, 1940: while the members of the genus *Proserpinicaris* Jakobi, 1972 clearly exhibit short-range endemism in Korea (Karanovic et al. 2012b), members of the genus *Parastenocaris* Kessler, 1913 seem to be more widely distributed (Karanovic and Lee 2012), although restricted to either Korea or Japan. This all suggests that the land bridges formed between Korea and Japan during the Pleistocene glacial cycles were sufficient for the dispersal of stygofauna, but the barriers that formed afterwards were sufficient to prevent significant gene flow and resulted in speciation as a rule. Our hypothesis about the isolated pockets of carbonate rocks in Korea acting as subterranean islands and harbouring short-range endemics was thus only partly supported. Renewed interest in stygofauna both in Korea and Japan will certainly result in the discovery of many endemic copepod species, and will provide further means of testing different hypothesis about the colonization history and evolution of subterranean environments.

The *languidoides*-group already contains 11 species in East Asia, and here we provide a key to aid in their identification.

### Key to East Asian species of the *languidoides*-group

**Table:** 

1	Female antennula 11-segmented, swimming legs segmentation (exopod/endopod) 2/2, 3/2, 3/3, 3/3	2
–	Female antennula of more than 11 segments	other *Diacyclops* Kiefer, 1927
2	Innermost terminal caudal seta as long as or shorter than outermost terminal caudal seta	3
–	Innermost terminal caudal seta more than twice as long as outermost terminal caudal seta, about as long as caudal rami	*Diacyclops japonicus* Ito, 1952
3	Antenna with exopod	4
–	Antenna without exopod	5
4	Caudal rami more than four times as long as wide	*Diacyclops languidoides* (Lilljeborg, 1901)
–	Caudal rami about three times as long as wide	*Diacyclops leeae* sp. n.
5	Innermost terminal caudal seta minute; caudal rami 1.6 times or less as long as wide; basis of antenna with single seta	6
–	Innermost terminal caudal seta longer than half width of caudal ramus; caudal rami at least twice as long as wide; basis of antenna with more than one seta	7
6	Habitus wide and dorsoventrally compressed; aesthetasc on eighth segment of female antennula reaching terminal segment; basis of maxilliped with two setae	*Diacyclops parahanguk* sp. n.
–	Habitus not dorsoventrally compressed; aesthetasc on eighth segment of female antennula shorter than above; basis of maxilliped with single seta	*Diacyclops hanguk* sp. n.
7	Caudal rami about twice as long as wide; dorsal caudal seta only slightly reaching beyond distal tip of outermost terminal caudal seta	8
–	Caudal rami more than twice as long as wide; dorsal caudal seta reaching significantly beyond distal tip of outermost terminal caudal seta	9
8	Innermost terminal caudal seta about as long as outermost terminal caudal seta	*Diacyclops ishidai* sp. n.
–	Innermost terminal caudal seta shorter than outermost terminal caudal seta	*Diacyclops brevifurcus* Ishida, 2006
9	Dorsal caudal seta very long, almost as long as outer principal terminal seta	10
–	Dorsal caudal seta at most half as long as outer principal terminal seta	11
10	Innermost terminal caudal seta shorter than outermost terminal caudal seta	*Diacyclops suoensis* Ito, 1954
–	Innermost terminal caudal seta longer than outermost terminal caudal seta	*Diacyclops pseudosuoensis* sp. n.
11	Habitus slender in dorsal view; lateral arms of seminal receptacle pointing laterally in ventral view	*Diacyclops parasuoensis* sp. n.
–	Habitus wide and dorso-ventrally compressed; lateral arms of seminal receptacle pointing antero-laterally	*Diacyclops hisuta* sp. n.

## Supplementary Material

XML Treatment for Diacyclops ishidai

XML Treatment for Diacyclops brevifurcus

XML Treatment for Diacyclops parasuoensis

XML Treatment for Diacyclops suoensis

XML Treatment for Diacyclops pseudosuoensis

XML Treatment for Diacyclops hisuta

XML Treatment for Diacyclops leeae

XML Treatment for Diacyclops hanguk

XML Treatment for Diacyclops parahanguk
